# Inhibitors of the Bacterioferritin Ferredoxin Complex Dysregulate Iron Homeostasis and Kill and Biofilm-Embedded Cells

**DOI:** 10.1021/acsinfecdis.5c00209

**Published:** 2025-06-09

**Authors:** Alexanndra M. Behm, Huili Yao, Emmanuel C. Eze, Suliat A. Alli, Simon D. P. Baugh, Ebenezer Ametsetor, Kendall M. Powell, Kevin P. Battaile, Steve Seibold, Scott Lovell, Richard A. Bunce, Allen B. Reitz, Mario Rivera

**Affiliations:** † Department of Chemistry, 5779 Louisiana State University, Baton Rouge, Louisiana 70803, United States; ‡ 716094 Fox Chase Therapeutics Discovery, Inc, Doylestown, Pennsylvania 18902, United States; § Department of Chemistry, 7618 Oklahoma State University, Stillwater, Oklahoma 74078, United States; ∥ 535965 New York Structural Biology Center, New York, New York 10027, United States; ⊥ Protein Structure and X-ray Crystallography Laboratory, Del Shankel Structural Biology Center, 4202 University of Kansas, Lawrence, Kansas 66047, United States

**Keywords:** antibiotic, biofilm, iron homeostasis, bacterioferritin, iron storage

## Abstract

In , the iron storage protein bacterioferritin (Bfr) contributes to buffering cytosolic free iron concentrations by oxidizing Fe^2+^ and storing the resultant Fe^3+^ in its internal cavity, and by forming a complex with a cognate ferredoxin (Bfd) to reduce the stored Fe^3+^ and mobilize Fe^2+^ to the cytosol. Small molecule derivatives of 4-aminoisoindoline-1,3-dione designed to bind Bfr (Pa Bfr) at the Bfd binding site accumulate in the cell, block the Pa Bfr–Bfd complex, inhibit iron mobilization from Pa Bfr, elicit an iron starvation response, are bacteriostatic to planktonic cells, and are bactericidal to biofilm-entrenched cells. A structural alignment of Pa Bfr and Bfr (Ab Bfr) showed strong conservation of the Bfd binding site on Ab Bfr. Accordingly, the small molecule inhibitors of the Pa Bfr–Bfd complex accumulate in the cells, elicit an iron starvation response, are bactericidal to planktonic cells, and exhibit synergy with existing antibiotics. These findings indicate that the inhibition of iron mobilization from Bfr may be an antimicrobial strategy applicable to other Gram-negative pathogens.

## Graphical abstract

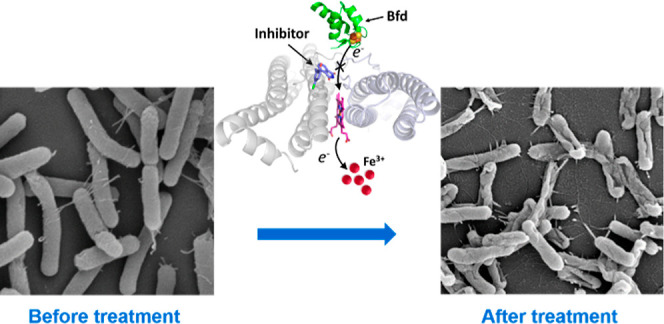

The emergence of antimicrobial-resistant bacteria poses a significant threat to public health. In 2018, the World Health Organization (WHO) released a list of priority pathogens, which includes the Gram-negative, carbapenem-resistant , and Enterobacterales as organisms for which new antibiotics are critically needed.^1^ Infections caused by these organisms are difficult to treat and are associated with high morbidity and mortality.^2^ is an opportunistic pathogen associated with outbreaks and hospital infections, which exhibits several innate mechanisms of antibiotic resistance and a marked propensity to acquire resistance to antimicrobial agents, including carbapenem. The most prevalent infections caused by , often associated with multidrug resistance, include hospital- and community-acquired pneumonia, meningitis, and urinary tract and skin wound infections. is also associated with ventilator-associated pneumonia and bloodstream infections, which have death rates of up to 35%.^3–5^ , one of the leading pathogens associated with hospital infections has a propensity to form biofilms colonizing wounds, endotracheal tubes, urinary catheters,^6^ and the lungs of cystic fibrosis patients.^7^ Resistant infections continue to rise worldwide and are associated with high mortality, morbidity, and health care costs.^8,9^

There is an urgent need to discover new antibiotics and validate new targets for the development of novel therapeutic alternatives for combating multidrug-resistance infections. In this context, bacterial iron homeostasis offers a vulnerability to exploit because invading pathogenic bacteria depend on host iron to support their metabolic needs, but the host immune defenses restrict the nutrient availability, such that levels of free iron in the host are vanishingly low (∼10^–18^ M).^10,11^ Iron restriction by the host, therefore, establishes a hostile environment for bacterial cells that require levels of free iron several orders of magnitude higher (10^–5^ to 10^–7^ M).^12,13^ Consequently, the survival of invading pathogens depends on well-regulated iron homeostasis, which can be succinctly thought of as a combination of strategies that involve sensing and responding to intracellular iron levels.^14^ Low environmental iron levels, such as those encountered in the host, lead to low iron levels in bacterial cells, which stimulate the transcription of genes encoding proteins for the biosynthesis of iron-capture and iron-uptake systems, the mobilization of iron from iron storage proteins, and the subsequent incorporation of iron in proteins participating in bacterial cell metabolism.^14^

Our laboratories have been investigating iron storage proteins^15–19^ and the consequences of inhibiting the mobilization of iron from iron storage molecules^14^ in the opportunistic pathogen . The iron storage protein bacterioferritin (Bfr) is central to iron metabolism because it functions by catalyzing the oxidation of Fe^2+^ and encapsulating the resultant Fe^3+^ in its interior cavity, which not only minimizes the participation of Fe^2+^ in oxidative Fenton processes but also enables the intracellular accumulation (storage) of iron to concentrations much higher than is allowed by the low solubility of Fe^3+^ at physiological pH.^14,15^ Bfr is a nearly spherical protein (∼120 Å diameter) harboring a hollow cavity (∼80 Å diameter) where approximately 2000 Fe^3+^ ions can be stored.^14,15^ Bfr (Pa Bfr) is a 24-mer heteropolymer assembled from two types of homosubunit dimers, FtnA and the heme binding BfrB.^16^ The mobilization of Fe^3+^ stored in Pa Bfr requires binding a Bfr associated ferredoxin (Pa Bfd).^20^ Pa Bfd binds Pa Bfr at a site formed at the interface of each Pa BfrB subunit dimer such that the [2Fe–2S] cluster in Pa Bfd is placed ∼22 Å from the heme iron in the BfrB subunit dimer (Figure 1A). The Pa Bfd binding site on each Pa BfrB subunit dimer is a shallow depression on the surface demarcated by residues L68, P69, N70, and Q72 from one subunit and L78, L79, G80, and E81 from the accompanying subunit, where Pa Bfd residues M1, Y2, and L5 bind (Figure 1B).^20^ Pa Bfd binds to Pa Bfr with *K*_d_ = 4.7 μM,^21,22^ and electron transfer from the [2Fe–2S] cluster in Pa Bfd to the Fe^3+^ stored in Pa Bfr via the heme in the Bfr subunit dimer enables the mobilization of Fe^2+^ to the cytosol.^20,22–24^ Deletion of the *bfd* gene in (Δ*bfd*) causes an irreversible accumulation of iron in Pa Bfr, which results in intracellular iron limitation,^23^ inability to mature biofilms,^25^ and metabolic dysregulation.^26^

**Figure 1:**
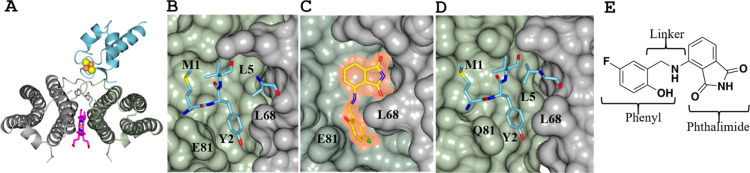
Inhibitors of the Pa Bfr–Bfd complex. (A) Pa Bfd binds at the interface of a Pa Bfr subunit dimer, placing the [2Fe–2S] cluster of Bfd above the heme in Bfr. (B) Close up view of the Pa Bfr–Bfd interface depicting the Pa Bfr residues in surface rendering and the Pa Bfd residues anchoring on the surface in sticks. (C) Crystal structure of 4-aminoisoindoline-1,3-dione derivative (JAG-5–7) bound to the Bfd binding site on the Pa Bfr surface illustrates how the 4-aminoisoindoline-1,3-dione (phthalimide) bicycle and the phenyl ring bind at the pocket that would be occupied by L5 and Y2 in Bfd, respectively. (D) The crystal structure of Ab Bfr revealed structural conservation of the Bfd binding site, where inhibitors of the Bfr–Bfd complex are expected to bind in a manner akin to that observed with Pa Bfr. (E) The structure of JAG-5–7 illustrates the conceptual dissection of the pharmacophore.

These observations encouraged our laboratories to develop small molecule inhibitors of the Pa Bfr–Pa Bfd complex. This objective was initiated by screening a fragment library and followed by iterative structure-guided design of hit fragments that bind at the Bfd site on Pa Bfr.^27^ The studies led to the discovery of 4-aminoisoindoline-1,3-dione derivatives (phthalimide derivatives), such as analogue 11 (Table S1), which binds Pa Bfr at the Pa Bfd binding site.^27^ The X-ray crystal structures of Pa Bfr bound to several analogues (e.g., JAG-5–7 in Figure 1C, PDB 7K5E) demonstrated that the phthalimide moiety binds Pa Bfr with a unique pose and in the same pocket where L5 from Pa Bfd binds, while the linker and phenyl ring extend the analogues to interact with the cleft formed by the side chains of L68 and E81.^27,28^ The 4-aminoisoindoline-1,3-dione derivatives are bacteriostatic against planktonic and bactericidal against biofilm-entrenched cells.^28^ Amino acid sequence alignments of the Bfr or Bfd proteins in with the corresponding proteins in , and revealed high conservation of residues critical to the stability of the Pa Bfr–Pa Bfd complex, suggesting that compounds capable of inhibiting the Pa Bfr–Pa Bfd complex in may also inhibit the Bfr–Bfd complex in these organisms.^21^ Moreover, the isolation of Bfr from cells (Ab Bfr) and its subsequent structural characterization showed that Ab Bfr is also a heteropolymer assembled from Ftn and Bfr subunit dimers.^21^ Its structure (PDB 9BTS) revealed that the Bfd binding site on Ab Bfr is identical to that on Pa Bfr, where the conserved M1, Y2, and L5 residues of Ab Bfd are expected to bind in a manner like that observed in the Pa Bfd–Pa BfrB complex^21^ (Figure 1D). Investigations in solution showed that Ab Bfd binds Ab Bfr with *K*_d_ = 5.6 μM,^21^ a value similar to that measured for the binding of Pa Bfd to Pa Bfr (4.7 μM).^16^ Taken together, these observations suggest that inhibitors developed for the Pa Bfr–Pa Bfd complex can be expected to bind Ab Bfr, block the binding of Ab Bfd and disrupt iron homeostasis in . The work presented here, which was aimed at testing this idea, demonstrates that the 4-aminoisoindoline-1,3-dione derivatives that are bacteriostatic against planktonic are bactericidal against biofilms and bactericidal against planktonic .

## Results and Discussion

To enable a systematic description of how modifications to the pharmacophore affect target binding and antimicrobial efficacy, the structure of the pharmacophore has been conceptually dissected into three sections: the 4-aminoisoindoline-1,3-dione (phthalimide) bicycle, the linker, and the phenyl ring, as illustrated in Figure 1E, with the structure of compound JAG-5–7. The structures of the compounds used in this study, grouped according to modifications made to distinct sections of the pharmacophore, are summarized in Tables S1–S4. While the tables summarize a significant portion of the information gathered, the experimental approach, the corresponding observations, and studies aimed at probing synergy with other antibiotics, target engagement, and structure of the pharmacophore bound to Pa Bfr are illustrated below with compounds KM-5–25 and KM-5–35, whose structures can be found in Table S1.

## 4-Aminoisoindoline-1,3-Dione Derivatives Are Bacteriostatic against Planktonic and Bactericidal against 

The structural conservation of the Bfd binding site on the structures of Pa Bfr and Ab Bfr suggests that the 4-aminoisoindoline-1,3-dione derivatives developed with the guidance of the Pa Bfr–Bfd complex structure can be expected to bind Ab Bfr, block the binding of Ab Bfd and disrupt iron homeostasis in by irreversibly trapping iron in Ab Bfr. To test this hypothesis, we challenged and three distinct strains with 4-aminoisoindoline-1,3-dione derivatives. For example, Figures 2 and S1, respectively, show the results of challenging bacteria with compounds KM-5–35 and KM-5–25 and monitoring cell growth following the optical density at 600 nm (OD_600_). In the case of , the compounds inhibit growth in a dose-dependent manner (Figures 2A and S1A), but even at the highest concentration used (dictated by aqueous solubility), growth is not completely arrested. Enumeration of viable cells (CFU/mL) at the end of growth for each of the compound concentrations was used to calculate the concentration that inhibits growth by 50% (IC_50_) (Figures 2B and S1B). In contrast, KM-5–35 or KM-5–25, completely inhibit the growth of cells (Figures 2C and S1C), thus allowing the determination of minimum inhibitory concentrations (MIC). Similar observations have been made with other 4-aminoisoindoline-1,3-dione derivatives (Tables S1–S4) indicating that current inhibitors of the Bfr–Bfd complex are more active against planktonic than against planktonic .

**Figure 2:**
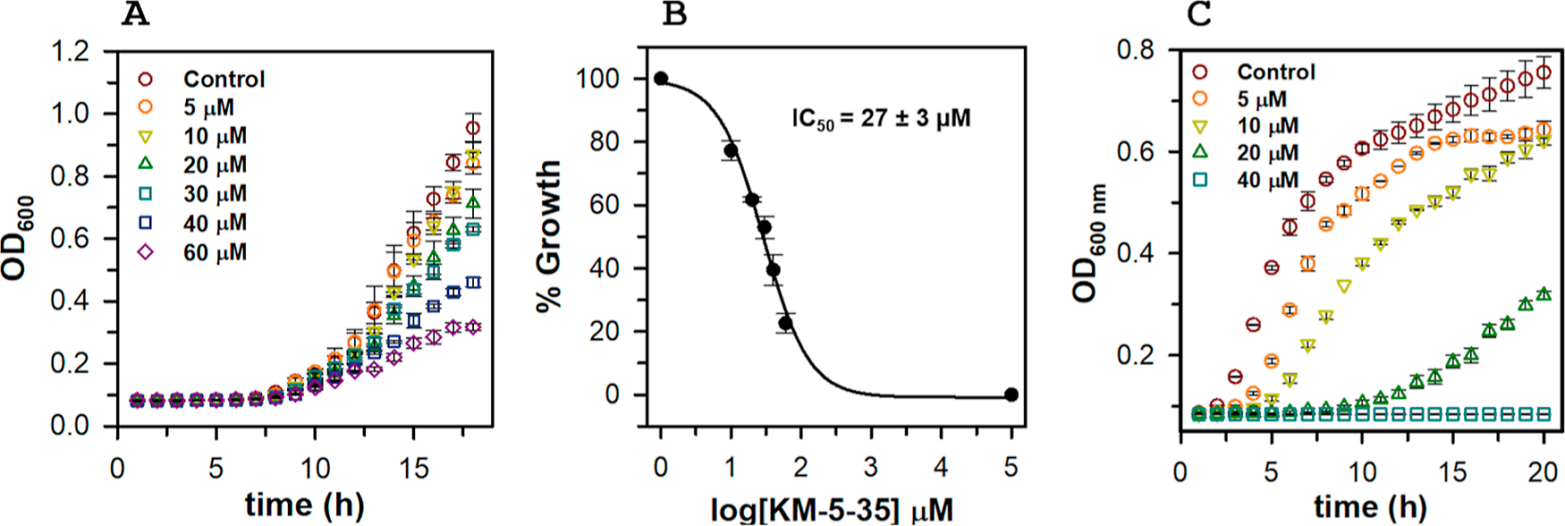
4-Aminoisoindoline-1,3-dione derivatives elicit a growth defect in planktonic and . (A) Concentration-dependent growth retardation of PAO1 treated with KM-5–35 and (B) associated IC_50_ value obtained from fitting the %growth of (CFU/mL) as a function of inhibitor concentration to eq S1 in Supporting Information; the log­[KM-5–35] = 5 value is included to define the minimum asymptote for the fit. (C) Concentration-dependent growth retardation of 5075 treated with KM-5–35. Each of the growth curves was constructed from the average and standard deviation of 3 replicate wells. The IC_50_ and MIC values are the average from three independent experiments.

To ascertain whether the 4-aminoisoindoline-1,3-dione derivatives are bactericidal against , we conducted time kill experiments in which 5075, a multidrug resistant highly virulent isolate,^29^ was challenged with KM-5–25 or KM-5–35. The results obtained with KM-5–35 (Figure 3A) show that the presence of the compound at the MIC results in a >3log_10_ reduction of viable cells (CFU/mL) relative to the inoculum, indicating that KM-5–35 exerts a bactericidal action on the cells.^30^ The time-dependent data show that KM-5–35 kills 5075 faster than meropenem but slower than colistin when each of the antibiotics is utilized at the corresponding MIC (Table 1). The results obtained with KM-5–25 (Figure 3B) show that when the compound is present at the MIC, the rate of killing is slower than that observed with KM-5–35, and although the number of viable cells is reduced by approximately 3log_10_ relative to the inoculum in approximately 3 h, cell growth is observed at later hours. When KM-5–25 is present at 1.5 × MIC (limit of aqueous solubility), the compound is bactericidal, killing the cells at a rate similar to that observed with KM-5–35.

**Figure 3:**
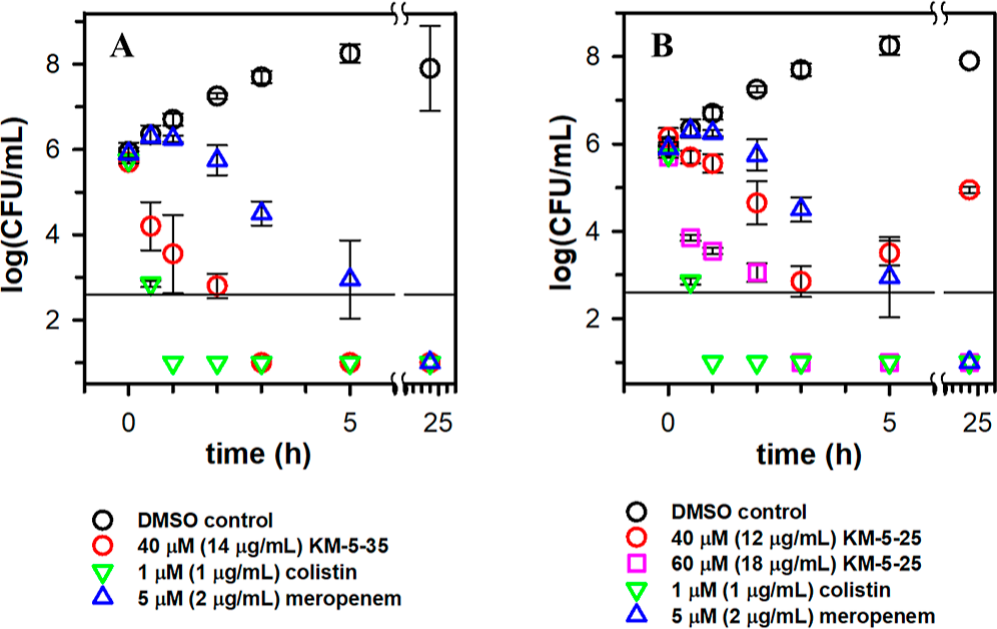
4-Aminoisoindoline derivatives are bactericidal against planktonic 5075. Time-kill assays comparing the bactericidal action of colistin and meropenem used at 1 × MIC with (A) KM-5–35 at 1 × MIC and (B) KM-5–25 at 1 × MIC and 1.5 × MIC. The horizontal line indicates the lower limit of detection. The plots were constructed from the average and standard deviation of three independent experiments.

**Table 1:** Checkerboard Assay Results

MIC (μg/mL)				
alone		combination		FIC
colistin	KM-5–35^a^	colistin	KM-5–35^a^	
1	14	0.0625	3.5	0.31
		0.0312	3.5	0.28
		0.25	1.8	0.38
		0.125	1.8	0.25
imipenem	KM-5–35^a^	imipenem	KM-5–35^a^	
1	14	0.25	3.5	0.50
		0.125	3.5	0.38
ciprofloxacin	KM-5–35^a^	ciprofloxacin	KM-5–35^a^	
128	14	4	7	0.53
ceftazidime	KM-5–35^a^	ceftazidime	KM-5–35^a^	
256	14	2	7	0.50
		1	7	0.50
		0.5	7	0.50
		0.25	7	0.50
meropenem	KM-5–35^a^	meropenem	KM-5–35^a^	
2	14	0.5	3.5	0.50
		0.002	7	0.50
		0.004	7	0.50
				
tobramycin	KM-5–35^a^	tobramycin	KM-5–35^a^	
512	14	4	7	0.50
		2	7	0.50
		1	7	0.50
		0.5	7	0.50

a 14, 7, 3.5, and 1.8 μg/mL = 40, 20, 10, and 5 μM, respectively.

Checkerboard assays with were carried out to evaluate the possible interactions that are present when KM-5–35 is used in combination with antibiotics representative of four distinct classes: aminoglycosides (tobramycin), carbapenems (imipenem and meropenem), fluoroquinolones (ciprofloxacin), and polymyxins (colistin). Checkerboard plots of the distinct combinations are presented in Figure 4, and Table 1 lists the MIC of the antimicrobial agents used alone and in combination as well as the fractional inhibitory concentration index (FIC) for each combination. The data show synergism (FIC < 0.5) between KM-5–35 and either colistin or imipenem, while combinations of KM-5–35 and either ceftazidime, meropenem, ciprofloxacin, or tobramycin are additive (0.5 < FIC < 2). Note that 5075 is resistant to ceftazidime, tobramycin, and ciprofloxacin. In the case of ciprofloxacin (MIC = 128 μg/mL), when KM-5–35 is present at 7 μg/mL, ciprofloxacin present at 4 μg/mL is sufficient to inhibit growth (FIC = 0.53). In the presence of 7 μg/mL KM-5–35, ceftazidime (MIC = 256 μg/mL) present at 0.25 μg/mL, or tobramycin (MIC = 512 μg/mL) present at 0.5 μg/mL is sufficient to inhibit the growth of the resistant strain. We also carried out checkerboard assays with KM-5–25 and the two antibiotics that show synergism with KM-5–35, colistin, and imipenem. The results (Figure S3) indicate that KM-5–25 exhibits synergy with colistin (FIC = 0.36, 0.38) and is additive with imipenem (FIC = 0.63).

**Figure 4:**
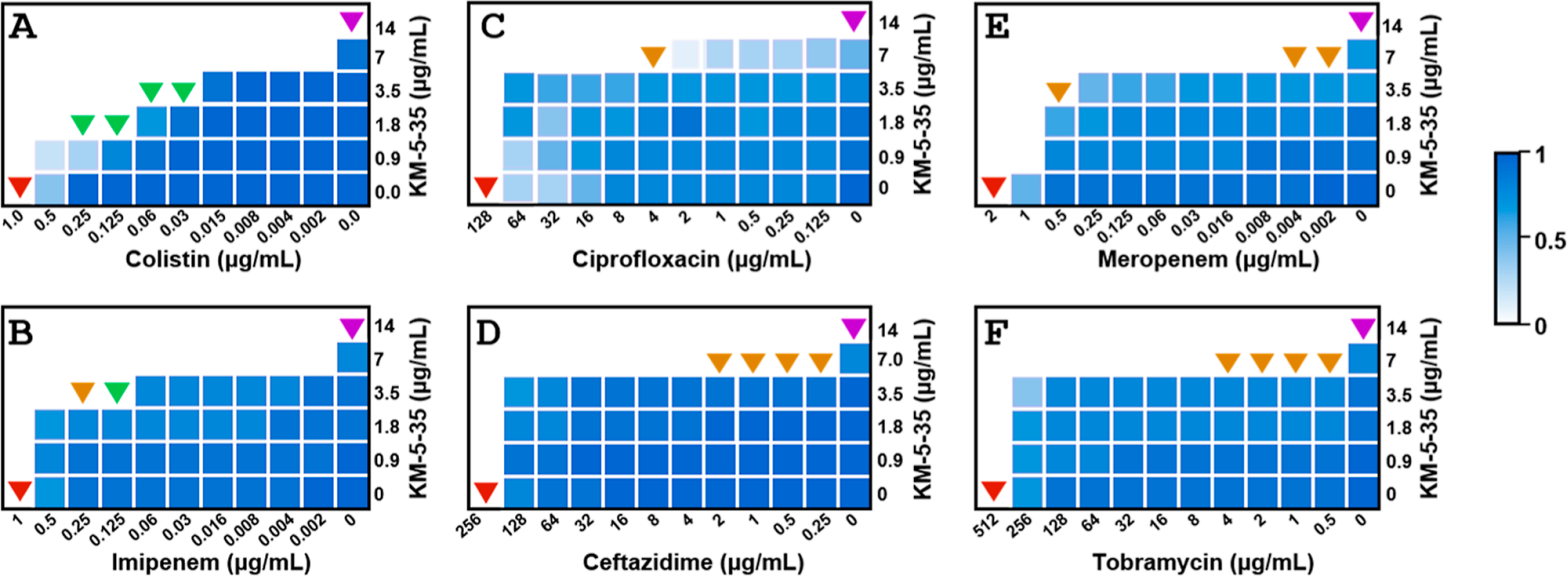
Checkerboard microdilution assay between KM-5–35 and (A) colistin, (B) imipenem, (C) ciprofloxacin, (D) ceftazidime, (E) meropenem, and (F) tobramycin against 5075. Bacterial growth monitored by OD_600_ is represented as a heat map from white (no growth) to blue. The red and purple triangles, respectively, indicate MIC values for antibiotic and KM-5–35; the green and orange triangles, respectively, indicate concentrations, where antibiotic and KM-5–35 exhibit synergistic and additive interaction. The data are representative of three biological replicates.

### 4-Aminoisoindoline-1,3-Dione Derivatives Kill Cells in Mature Biofilms

We studied the susceptibility of mature biofilms to treatment with analogues of 4-aminoisoindoline-1,3-dione using biofilms cultured at the solid–liquid interface (pellicles) using methodology reported previously.^28^ To determine the susceptibility of pellicle biofilms to inhibitors of the Pa Bfr–Bfd complex, we cultured 27 h old biofilms in PI media supplemented with 20 μM Fe, as presented in Experimental Methods. The mature biofilms were transferred onto glass coverslips by touching the pellicle with the surface of the coverslips. The coverslip-adhered biofilms were then exposed to treatment solution consisting of AB media supplemented with 20 μM Fe, 1.5% DMSO, 0.025% HPMC, and 4-aminoisoindoline-1,3-dione derivative or commercial antibiotic for 24 h. The biofilms were then harvested and washed, and the cells were dispersed into sterile PBS by vortexing in the presence of zirconia beads prior to plating and enumerating viable cells (CFU/mL) to calculate % cell survival. As can be observed in Figure 5, treatment of the pellicles with KM-5–25 or KM-5–35 elicits concentration-dependent biofilm cell death.

**Figure 5:**
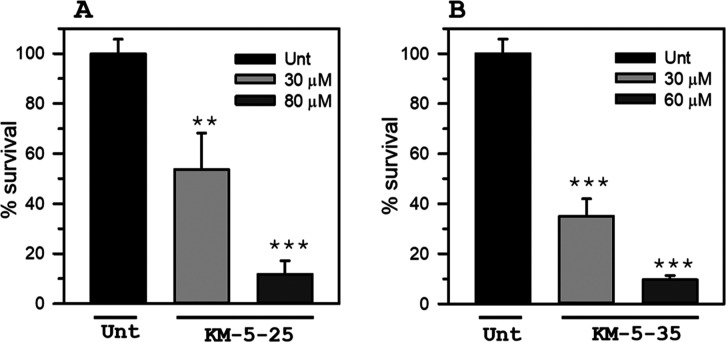
(PAO1) cells in mature biofilms are susceptible to 4-aminoisoindoline-1,3-dione analogues. cells embedded in 27 h old biofilms treated for 24 h with (A) KM-5–25 or (B) KM-5–35 were dispersed for enumeration of viable cells. The % survival is expressed as the ratio CFU/mL_(compound treated)_/CFU/mL_(untreated control)_. p < 0.01 denoted by **, p < 0.001 denoted by *** relative to untreated.

The coverslip-adhered biofilms treated with KM-5–25 or KM-5–35 were also investigated by scanning electron microscopy (SEM). As expected for mature biofilms, the images of the untreated control (Figure 6A) show a well-defined biofilm exhibiting the characteristic embedding of rod-shaped cells in a matrix of extracellular polymeric substances attached to the glass substratum. In stark contrast, images of the biofilm treated with KM-5–35 revealed mostly dead cells embedded within the extracellular polymeric matrix (Figure 6B). These observations indicate that the 4-aminoisoindoline-1,3-dione derivative killed the biofilm-embedded cells rather than dispersed them. The morphology of the dead cells, which indicates cell envelope lysis and the release of intracellular contents, is similar to the morphology observed when biofilms are treated with H_2_O_2_.^31^ The treatment of mature biofilms with KM-5–25 produced similar observations (Figure S2). Attempts to grow mature biofilms using the three strains used in these investigations were unsuccessful.

**Figure 6:**
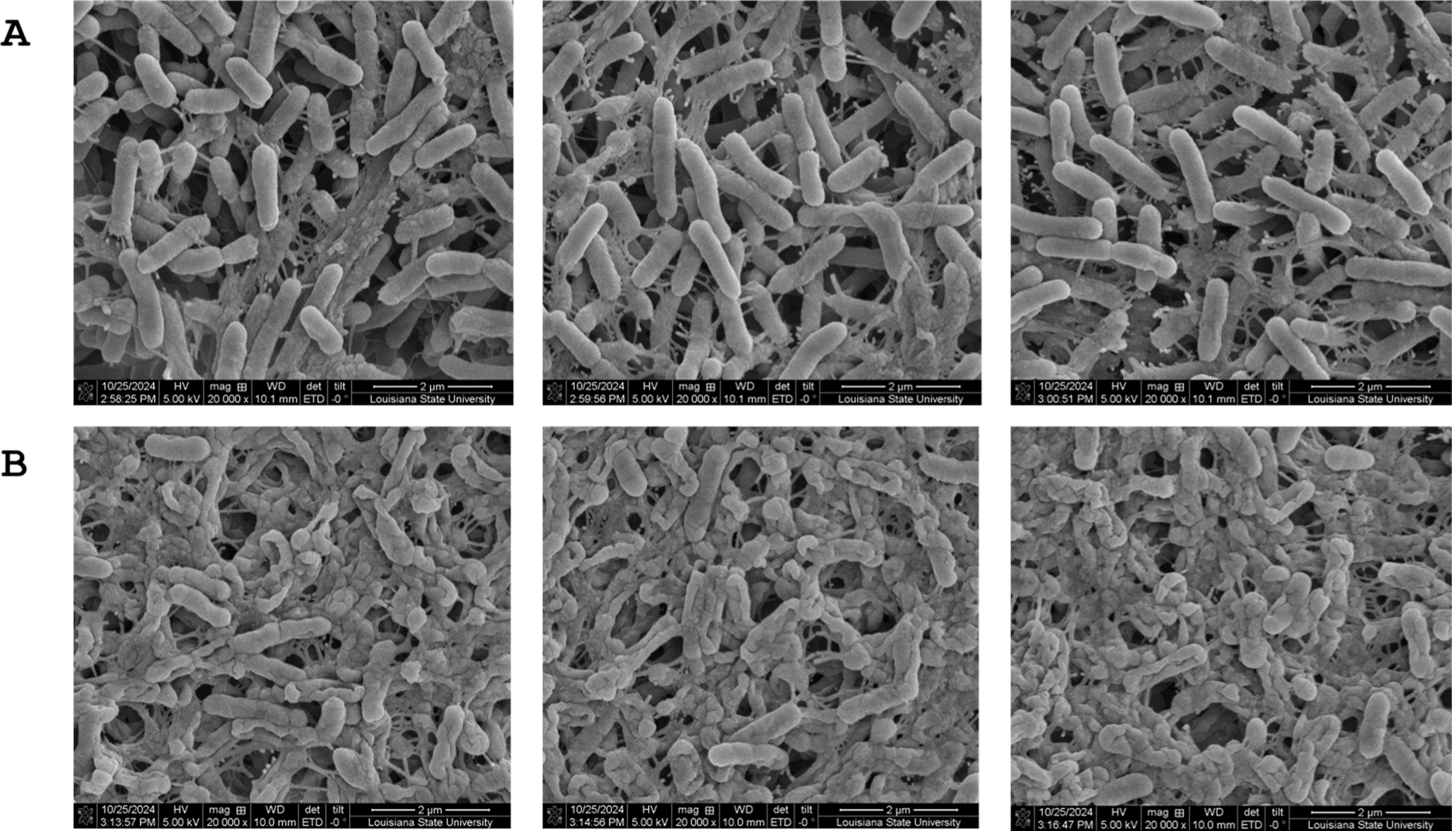
(PAO1) cells in mature biofilms are susceptible to 4-aminoisoindoline-1,3-dione analogues. Scanning electron microscopy (SEM) images showing (A) untreated PAO1 biofilm and (B) biofilm treated with 50 μM KM-5–35 for 24 h. Three representative images are shown for each condition.

### Structures of KM-5–25 and KM-5–35 Bound to Pa Bfr

We conducted ligand soaking experiments directed at obtaining cocrystals of inhibitors bound to recombinant Pa Bfr. These experiments allowed us to obtain X-ray crystal structures of KM-5–25 and KM-5–35 binding to Pa Bfr (Figure 7 and Table S5). As has been observed in previous structures of similar analogues bound to Pa Bfr,^27,28^ the 4-aminoisoindoline-1,3-dione (phthalimide) moiety binds at the Bfd binding site on Bfr with a conserved pose, establishing hydrogen bonds with the carbonyl O atom of P69 and the amide NH of L71. The linker and phenyl ring of both compounds interact with the cleft formed by the side chains of L68 and E81 in Bfr. The structure of KM-5–25 bound to Pa Bfr (Figure 7A) shows strong electron density consistent with KM-5–25 in several of the subunits, and although the phenyl ring exhibited conformational disorder in a few of the subunits, the subunits showing prominent electron density indicate that the Cl substituent of the phenyl ring is directed toward the floor of the cleft, while the hydroxyl group points toward the protein surface. Similar observations can be made in the structure of KM-5–35 bound to Pa Bfr, where a strong electron density consistent with KM-5–35 (Figure 7C) can be observed in many of the subunits. The phthalimide moiety is defined by strong electron density in all subunits, where it adopts the same pose as that observed by the phthalimide moiety in the KM-5–25 structure and in the structures of all 4-aminoisoindoline-1,3-dione derivatives solved so far.^27,28^ In most of the subunits displaying prominent KM-5–35 electron density, the Br atom in the phenyl ring is directed toward the interior of the cleft formed by the side chains of L68 and E81. In the few instances, where the phenyl ring of the inhibitor displayed conformational disorder, the Br atom was modeled toward the surface. It should be pointed out, however, that this situation probably arises from artificial stabilization of the phenyl conformation by crystal contacts.

**Figure 7:**
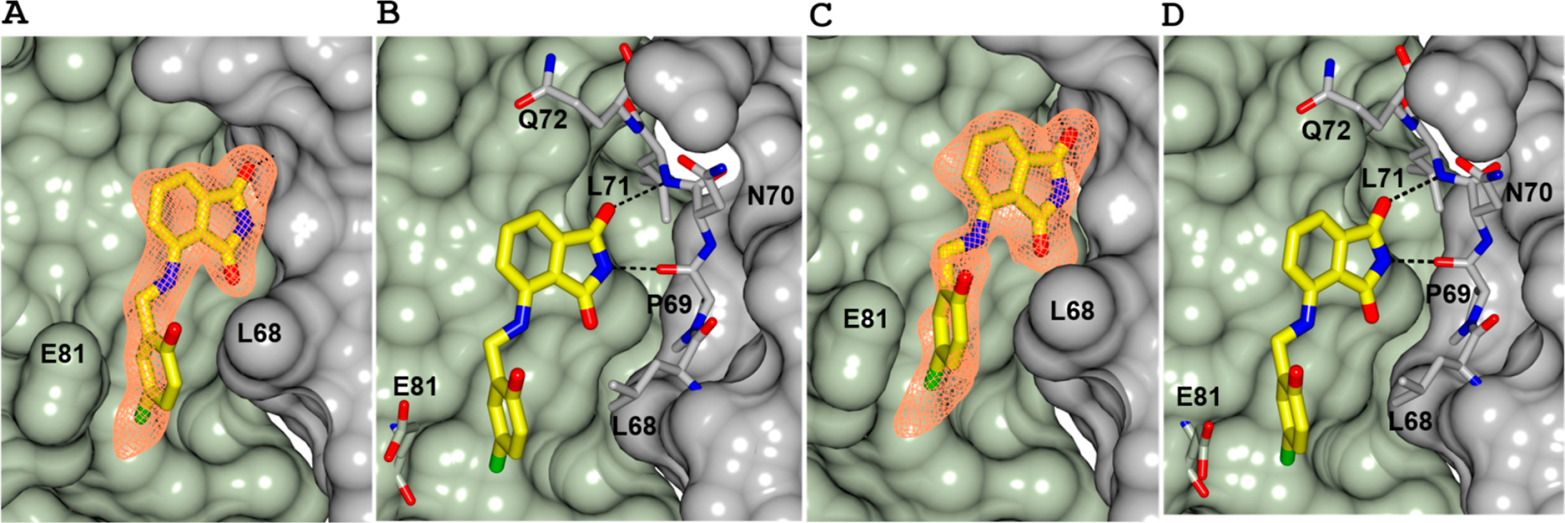
4-Aminoisoindoline-1–3-dione analogues bind at the Bfd binding site of Pa Bfr with a conserved pose. Subunits A and B of a Pa Bfr subunit dimer are rendered as surface and colored gray and green, respectively. Electron density (Fo–Fc) polder omit map (coral mesh) of KM-5–25 (A) and KM-5–35 (C) contoured at 3σ. Hydrogen bond interactions between Pa Bfr and KM-5–25 (B) or KM-5–35 (D) are shown as dashed lines. The Cl atom of KM-5–25 and Br atom of KM-5–35 are depicted in green.

###  and Cells Challenged with 4-Aminoisoindoline-1,3-Dione Derivatives Overproduce Siderophores

Given the strong sequence and structural conservation of the Bfd binding site on the and Bfr structures (see above), it is reasonable to conclude that 4-aminoisoindoline-1,3-dione derivatives bind Ab Bfr and inhibit the binding of Ab Bfd. It has been previously demonstrated that blocking the Bfr–Bfd complex in cells leads to an irreversible accumulation of iron in Bfr and concomitant iron depletion in the cytosol, which triggers an iron starvation response that manifests as a siderophore overproduction phenotype.^23,25,28^ Siderophores are molecules secreted by bacteria to bind environmental Fe^3+^ with a very large binding affinity and to internalize the nutrient to the bacterial cell for its subsequent incorporation in iron metabolism.^32^ Therefore, if the 4-aminoisoindoline-1,3-dione derivatives penetrate the cell, bind Ab Bfr, and inhibit the Ab Bfr–Bfd complex, then the ensuing irreversible accumulation of iron in Ab Bfr is expected to elicit an iron starvation response, leading to a siderophore overproduction phenotype. Previous work has shown that planktonic cells either lacking the *bfd* gene (Δ*bfd*) or treated with the 4-aminoisoindoline-1,3-dione derivatives secrete the pyoverdine siderophore approximately ∼3-fold more than the wild type strain or untreated control.^25–28^ In agreement with these prior observations, treatment of planktonic cells with KM-5–25 or KM-5–35 elicits the overproduction of pyoverdine (Figure 8A), which can be detected in the culture supernatant by its characteristic fluorescence. In comparison, cells treated with inactive compounds EB-5–73 (Table S1) or SB-17–140 (Table S4) did not secrete more pyoverdine than the untreated control cultures.

**Figure 8:**
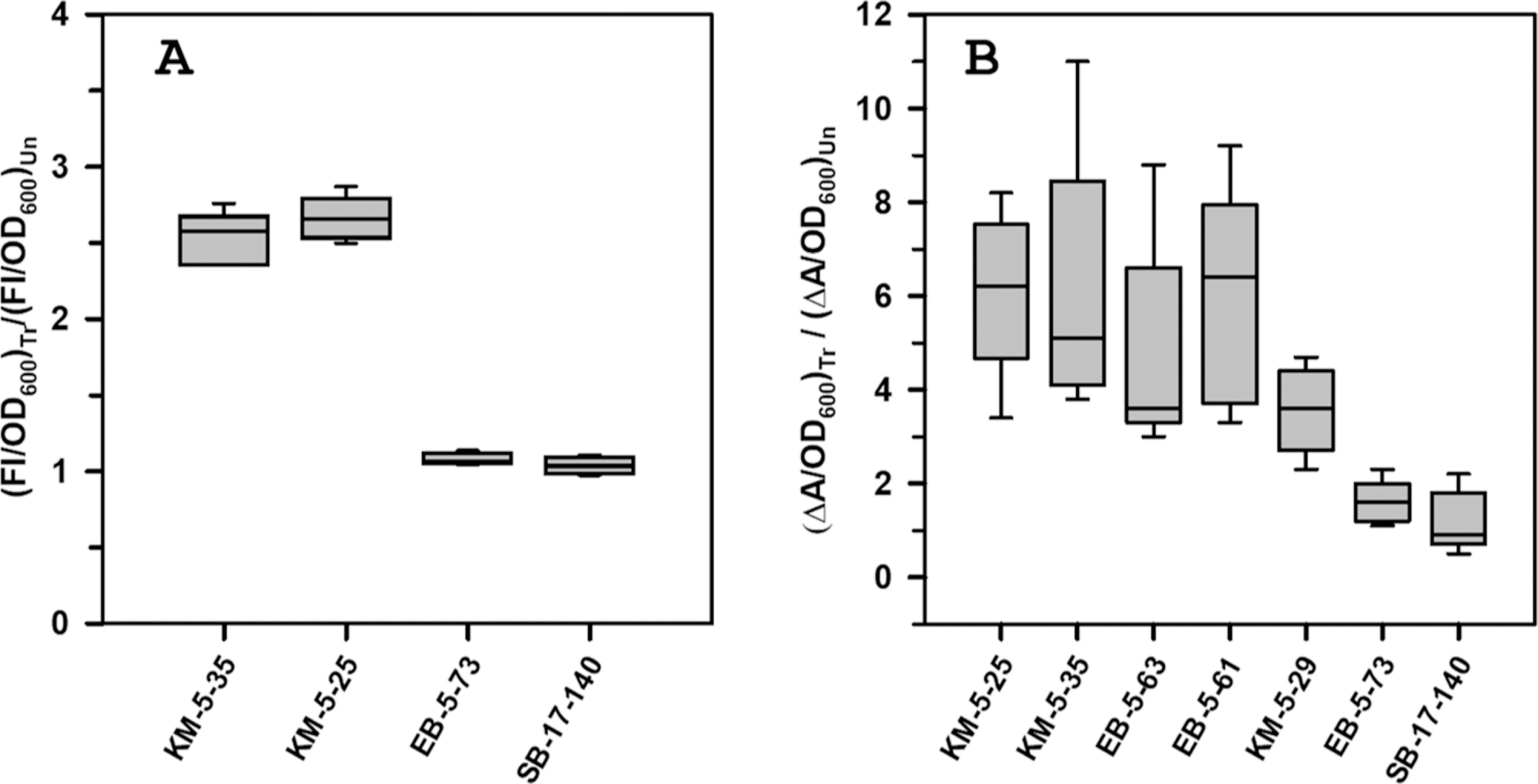
and planktonic cells treated with active 4-aminoisoindoline-1,3-dione derivatives overproduce siderophores. (A) The ratio of fluorescence intensity at 430 nm (FI) normalized to OD_600_ for treated (Tr) and untreated (Un) cells shows that cells treated with inactive compounds EB-5–73 (40 μM) or SB-17–140 (80 μM) secrete the same amount of pyoverdine as the untreated control, whereas treatment with active compound KM-5–25 (80 μM) and KM-5–35 (60 μM) elicits a pyoverdine overproduction phenotype. (B) Difference in the absorbance at 630 nm (ΔA) normalized to OD_600_ indicates that cells treated with active compounds KM-5–25 (40 μM), KM-5–35 (40 μM), EB-5–63 (40 μM), EB-5–61 (40 μM), and KM-5–29 (40 μM), secrete significantly higher levels of siderophore than cells treated with the inactive EB-5–73 (40 μM) or SB-17–140 (80 μM). The whisker plots were created with data from at least 5 biological replicates.

Unlike pyoverdine, the siderophores secreted by are not fluorescent. Consequently, to measure siderophore secretion from planktonic cultures of , we decided to utilize the Chrome azurol S (CAS) assay, a well-known universal colorimetric method used to detect siderophores.^33,34^ To this end, planktonic cells of 5075 were cultured for 20 h in the presence of sub-MICs of a representative number of inhibitors in M63 media not containing citrate, followed by analysis of the cell-free spent media by the CAS assay. Note that removal of the citrate from the M63 media, which was necessary to avoid interference of the citrate with the CAS assay, decreased the susceptibility of to the compounds such that growth was observed at concentrations equivalent to the MIC when citrate is present. Normalizing the signal to OD_600_ shows that, as expected, cells treated with the inhibitors of the Bfr–Bfd complex secrete approximately 4- to 5-fold more siderophores than the untreated controls (Figure 8B), while inactive compounds (EB-5–73 or SB-17–140) do not elicit a siderophore overproduction phenotype. These observations support the hypothesis that active compounds engage their target (Ab Bfr) and block the Ab Bfr–Bfd complex in , trapping iron in Ab Bfr and causing intracellular iron limitation, which in turn elicits a siderophore overproduction phenotype.

### Structure–Activity Relationships of the 4-Aminoisoindoline-1,3-Dione Derivatives

To understand how compound structure correlates with activity, a library of compounds was prepared and evaluated against the following metrics: (i) antimicrobial activities, (ii) aqueous solubility, (iii) binding affinity for Bfr (Pa Bfr) in vitro, and (iv) intracellular accumulation in PAO1 and in 5075 cells. To facilitate a systematic exploration of the pharmacophore, as indicated above, the structure of the 4-aminoisoindoline-1,3-dione derivatives has been conceptually dissected into three sections: the phthalimide bicycle, the linker, and the phenyl ring (Figure 1E). The relative efficacy of compounds against planktonic PAO1 cells was compared by measuring the IC_50_, and the relative activity against biofilms was compared by measuring % survival in the biofilm. The relative efficacy of compounds against planktonic cells was compared by measuring the MIC using three distinct strains, ATCC 17978 (AB 17978), ATCC 19606 (AB 19606), and the multidrug-resistant strain 5075 (AB 5075). Figure 9 depicts a schematic summary of the synthesis of six representative compounds, utilizing either reductive amination or *N*-alkylation for the *N*-benzylation of the 4-amino-isoindole-1,3-dione, in some cases followed by *O*-demethylation or Boc-deprotection, while the synthesis of each compound is presented in the Supporting Information, and the results are summarized in Tables S1–S4.

**Figure 9:**
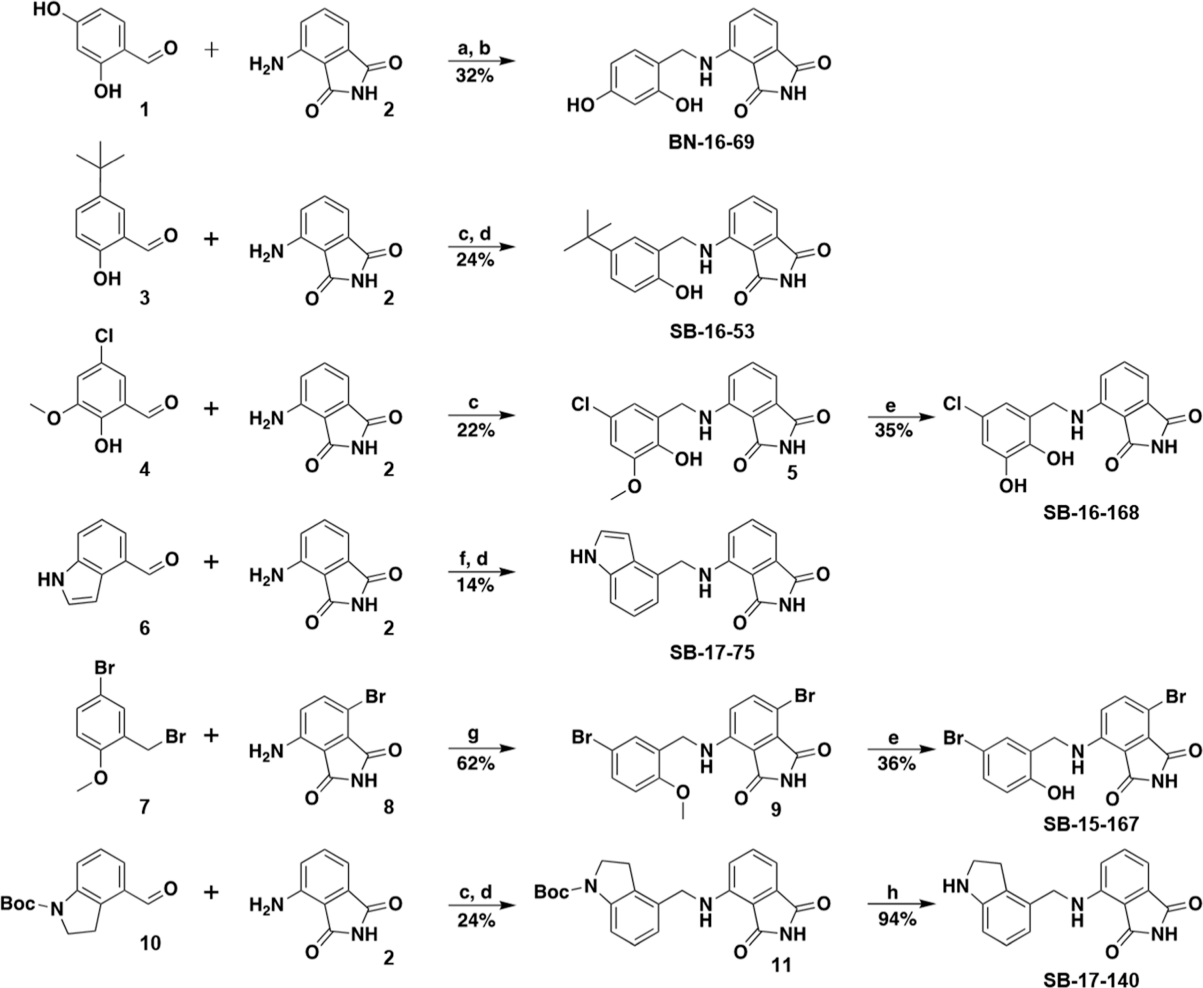
Schematic of the synthesis of six representative 4-aminoisoindoline-1,3-dione derivatives. (a) AcOH, DMF, 23 °C, 1 h; (b) NaBH­(OAc)_3_, DMF, 10 °C–23 °C, 18 h; (c) Ti­(OiPr)_4_, DMF, 20 °C, 5 h; (d) NaBH_4_, DMF, 0 °C–20 °C, 16 h; (e) BBr_3_, CH_2_Cl_2_, −78 °C–0 °C, 16 h; (f) Ti­(OiPr)_4_, DMF, 80 °C, 5 h; (g) DMF, 100 °C, 20 h; and (h) TFA, CH_2_Cl_2_, 20 °C, 16 h.

### Modifications to the Phenyl Ring of the Pharmacophore

Previous work showed that analogue 11 (Table S1), which has a hydroxyl substituent at position 2 of the phenyl ring is more active than equivalent structures with a hydroxyl substituent at position 3 or 4 in the phenyl ring.^27,28^ Consequently, the derivatives synthesized to explore changes in the phenyl ring can be categorized into three types: compounds harboring a phenyl ring with an invariant hydroxyl at position 2 and (i) one or (ii) two additional substituents and (iii) compounds with a heterocycle in place of the phenyl ring.

### Compounds with an Invariant 2-OH and One Additional Substituent on the Aryl Ring

The results from these studies are summarized in Table S1. Comparisons within each group with the same substitution pattern show a trend where the binding affinity (*K*_d_), intracellular accumulation, IC_50_, biofilm survival, and MIC are more favorable in the order *t*-Bu > Br ≈ Cl > F > CH_3_ > H > OH. Comparison across groups with distinct substitution patterns indicates that compounds with substituents at positions 2,4, 2,5, or 2,6 of the phenyl ring are similarly active (IC_50_ and MIC), whereas compounds with substituents at positions 2,3 of the phenyl ring are inactive or poorly active. In this context, it is noteworthy that the poorly active/inactive 2,3-phenyl-substituted compounds exhibit *K*_d_ values comparable and, in some cases, more favorable than compounds with 2,4-, 2,5-, or 2,6-phenyl substitution. The 2,3-substituted compounds, however, exhibit significantly lower intracellular accumulation in and cells than in their active isomeric counterparts. These observations underscore the importance of determining intracellular accumulation as a variable to understand relationships between structure and antimicrobial activity. In this context, it is interesting to note that almost invariably, the intracellular accumulation of the studied compounds in is several-fold larger than the intracellular accumulation in . It is therefore possible that the higher intracellular accumulation in may be a significant contributor to the bactericidal activity of the inhibitors against .

In attempts to increase the aqueous solubility of the Bfr–Bfd inhibitors, we prepared compounds bearing two hydroxyl substituents in the phenyl ring. Evaluation of these compounds showed that analogues with hydroxyl groups at positions 2,4- (BN-16–69), 2,5- (EB-5–93), and 2,6- (BN-16–70) of the phenyl ring are unstable in aqueous solution. The compound bearing hydroxyl groups at positions 2,3- of the phenyl ring (BN-4–74) is stable in aqueous solution but exhibits a high *K*_d_ value, does not accumulate intracellularly, and is not active.

### Compounds with an Invariant 2-OH and Two Additional Substituents in the Phenyl Ring

The results obtained with these compounds are presented in Table S2. In general, the installation of a third substituent (Br, Cl, or F) in the phenyl ring resulted in lower aqueous solubility relative to the disubstituted parent compounds and promoted improved binding affinity relative to that of the disubstituted parent molecules. In some cases, e.g., SB-16–112, the presence of the third substituent resulted in lower IC_50_ and MIC values relative to the corresponding disubstituted compounds, as well as improved activity against biofilms, as can be observed by comparing the % cell survival when biofilms are treated with 30 μM SB-16–112 relative to KM-5–25 or KM-5–35 (see Figure 5).

The observations made with compounds having two hydroxyl and one halogen substituent in the phenyl ring are interesting. With the exception of SB-16–186, these compounds are stable in aqueous solution. Compounds harboring a 2,4-dihydroxyl-5-Cl (or 5-Br) substitution (SB-16–160 and SB-16–137) exhibit low intracellular accumulation in and and poor antibacterial activity against both types of strains. Compounds with a 2,3-dihydroxyl-5-Cl (or 5-Br) substitution (SB-16–168 and SB-16–136) are active and exhibit IC_50_ values similar to those from parent compounds KM-5–25 and KM-5–35 (Table S1). The antibiofilm activity of SB-16–168 and SB-16–136, on the other hand, is significantly lower than that observed with KM-5–25 and KM-5–35. The reasons for the poor performance of SB-16–168 and SB-16–136 against biofilms are not fully understood, but we observed that biofilms treated with these compounds do not acquire the yellow tint observed when biofilms are treated with biofilm active compounds. Since the yellow tint in treated biofilms is caused by the presence of an inhibitor (yellow) in the biofilm matrix, where it can be taken by matrix-embedded cells, the lack of yellow color in biofilms treated with SB-16–168 and SB-16–136 suggests that these compounds cannot penetrate the biofilm matrix.

### Compounds with a Heterocycle in Place of the Phenyl Ring

Replacement of the phenyl ring in the pharmacophore with distinct heterocyclic rings (Table S4) produced mainly inactive compounds exhibiting low or nondetectable intracellular accumulation in and cells. There is only one exception, compound SB-17–75 where the phenyl ring has been replaced by an indole ring, exhibits *K*_d_, IC_50_, MIC, and antibiofilm activity values similar to those of KM-5–25 (see Table S1); it is known that an indole ring can be a bioisostere for a phenol.^35^ Although attempts to determine the intracellular accumulation of SB-17–75 in and cells were stymied by the very low fluorescence yield of the compound, its activity indicates that it is capable of accumulating intracellularly.

### Modifications to the Phthalimide Bicycle of the Pharmacophore

Installation of a halogen at carbon 7 of the phthalimide bicycle produced the compounds listed in Table S3. Although the aqueous solubility of most compounds decreased, those of compounds SB-16–67 and SB-16–122 were less affected. Both compounds exhibit *K*_d_ values in the single digit μM regime, accumulate intracellularly, and exhibit similar activity against planktonic and biofilm-embedded cells and against the three strains of *A. baumannii* utilized in this study.

## Conclusions

The current pipeline of antimicrobials used in the clinic consists mainly of improved versions of existing antibiotics that act on the traditional targets.^36^ Hence, the development of novel compounds capable of acting on previously unexploited targets offers the opportunity for innovative breakthroughs to enrich the repertoire of existing therapeutic options. Strategies directed at dysregulating bacterial iron homeostasis aim at exploiting this nontraditional target by capitalizing on the essentiality of iron as a bacterial nutrient in the context of the hostile environment imposed by immune withholding of iron in the host.^14,36,37^ Development of the strategy aimed at dysregulating iron homeostasis by inhibiting the mobilization of iron from Bfr was enabled by the crystal structure of the Bfr–Bfd complex^20^ and the elucidation of hot spot residues stabilizing the complex.^22^ Subsequent studies conducted with mutants either lacking the *bfd* gene (Δ*bfd*) or harboring a *bfr* mutant allele coding for a Bfr incapable of binding Bfd, demonstrated that blocking formation of the Bfr–Bfd complex in causes an irreversible accumulation of iron in Bfr and concomitant cytosolic iron starvation.^23^ Proteomics profiling showed that in addition to a pronounced iron starvation response, the Δ*bfd* mutant cells experience sulfur limitation, phenazine-mediated oxidative stress, altered carbon metabolism, and diminished amino acid biosynthesis,^26^ and studies conducted with biofilms demonstrated that the Δ*bfd* cells cannot form mature biofilms irrespective of environmental iron availability.^25^ A search for small molecule inhibitors of the Pa Bfr–Bfd interaction uncovered derivatives of 4-aminoisoindoline-1,3-dione, which can penetrate the cell, bind Pa Bfr, inhibit the mobilization of iron from Pa Bfr and elicit a pyoverdine overproduction phenotype similar to that seen with the Δ*bfd* cells.^27,28^

The more recent elucidation of the Bfr structure revealed near-complete conservation of the Bfd binding site relative to Pa Bfr,^21^ therefore suggesting that the 4-aminoisoindoline-1,3-dione derivatives can be expected to bind Ab Bfr and inhibit the Ab Bfr–Bfd complex. The work reported herein demonstrates that the 4-aminoisoindoline-1,3-dione derivatives elicit a siderophore overproduction phenotype in cells, indicating that as predicted, the 4-aminoisoindoline-1,3-dione derivatives can bind Ab Bfr and inhibit the Ab Bfr–Bfd complex, which results in an irreversible accumulation of iron in Ab Bfr and an iron deficiency in the cytosol. These ideas are supported by observations showing that inactive 4-aminoisoindoline-1,3-dione derivatives, which exhibit low to negligible intracellular accumulation, do not elicit a siderophore overproduction phenotype (see Figure 8).

The exploration of SAR reported here indicates that compounds such as KM-5–35 (Table S1) bearing a hydroxyl group at position 2 and a bulky halogen at position 4 or 5 of the phenyl ring exhibit a blend of desirable intracellular accumulation, potency against and , and aqueous solubility. Additional substituents on the phenyl ring can modestly increase potency but lower aqueous solubility (Table S2). It is also of note that although replacing the phenyl ring with a heterocycle (Table S4) resulted mostly in inactive compounds with negligible intracellular accumulation, compound SB-17–75 is active, which indicates that additional exploration of this area of the pharmacophore is likely to be fruitful. Similarly, the modifications made to the phthalimide ring of the pharmacophore have so far been limited (Table S3), but additional, more extensive exploration of this area of the pharmacophore may be productive. We will continue to work on exploring the compound series to find optimal lead compounds, with a view to finding the optimal balance of potency, solubility, and intracellular accumulation.

Given that the 4-aminoisoindoline-1,3-dione derivatives cause iron homeostasis dysregulation in and cells, it is not yet clear why the compounds are bactericidal against planktonic and bacteriostatic against planktonic cells. It is probable that the explanation is at least partially related to the 5- to 20-fold higher intracellular accumulation of the 4-aminoisoindoline-1,3-dione derivatives in cells relative to cells. The intracellular accumulation of xenobiotics in Gram-negative bacteria is dictated by permeability barriers imposed by the inner and outer membranes (OMs) and efflux pumps acting across both membranes. Despite similarities in OM architecture, and OM bilayers differ in thickness, charge distribution, dynamics, and proteins that support the structure of the OM and facilitate the selective uptake of nutrients, which together establish distinct permeabilities.^38^ It is therefore possible that differences in OM permeability, efflux, or both are at play in the higher intracellular accumulation of the 4-aminoisoindoline-1,3-dione derivatives in cells. It is also possible to consider that the bactericidal effect of the inhibitors on planktonic cells stems from a stronger binding affinity for Ab Bfr relative to that for Pa Bfr. Although the strong structural conservation of the Bfd binding site on Pa and Ab Bfr makes this scenario less probable, the binding affinity of the 4-aminoisoindoline-1,3-dione derivatives will be studied with recombinant Ab Bfr when the recombinant protein is expressed, purified, and characterized.

It is also not yet understood why the 4-aminoisoindoline-1,3-dione derivatives are bactericidal against biofilm-entrenched cells. One possibility is that the greater demand for iron in biofilms^25,39^ and other physiological differences between planktonic and sessile lifestyles sensitize biofilm-associated to iron homeostasis dysregulation, and subsequent cascading metabolic perturbations make the biofilm-associated cells more susceptible. In this context, it is interesting to note that the SEM images revealed that the morphology of dead cells treated with KM-5–35 and KM-5–25 compounds is similar to that observed when biofilms are treated with H_2_O_2_ because it suggests that although iron dysregulation is the primary target of the 4-aminoisoindoline-1,3-dione derivatives, ensuing oxidative stress may contribute to the bactericidal activity. Finally, it is also noteworthy that the 4-aminoisoindoline-1,3-dione derivatives exhibit synergy or additive interactions with commercial antibiotics against 5075, a highly virulent multidrug-resistant strain.

## Supplementary Material



## Notes

Concentration-dependent growth retardation of and and SEM of biofilm treated with KM-5–25; summarizing physicochemical and susceptibility data from compounds; aqueous solubility, *K*_d_, intracellular accumulation, IC_50_, MIC, and % biofilm survival; X-ray crystallography statistics; and compound characterization data (NMR and HRMS) (PDF)
