# Supplementary material for: Inhibitors of the Bacterioferritin Ferredoxin Complex Dysregulate Iron Homeostasis and Kill and Biofilm-Embedded Cells
Source: ACS Infect Dis. 2025 Jun 9;11(7):1983–93. doi: 10.1021/acsinfecdis.5c00209 (PMC12261321; doi:10.1021/acsinfecdis.5c00209)
Supplement: Supplementary file 1 [file id5c00209_si_001.pdf]

Supporting Information

Inhibitors of the Bacterioferritin Ferredoxin Complex Dysregulate Iron Homeostasis and Kill Acinetobacter baumannii and Biofilm-Embedded Pseudomonas aeruginosa cells

Alexanndra M. Behm[1,§]**,** *Huili Yao*[1,§], *Emmanuel C. Eze*[1], *Suliat A. Alli*[1], *Simon D. P. Baugh*[2], *Ebenezer Ametsetor*[3], *Kendall M. Powell*[1], *Kevin P. Battaile*[4], *Steve Seibold*[5], *Scott Lovell*[5], *Richard A. Bunce*[3], *Allen B. Reitz*[2], *Mario Rivera*[1]*

[1]Department of Chemistry, Louisiana State University, Baton Rouge, LA, 70803, USA

[2]Fox Chase Therapeutics Discovery, Inc., Doylestown, PA 18902, USA

[3]Department of Chemistry, Oklahoma State University, Stillwater, OK, 74078, USA

[4]New York Structural Biology Center, New York, NY 10027, USA

[5]Protein Structure and X-ray Crystallography Laboratory, Del Shankel Structural Biology Center, University of Kansas, Lawrence, KS 66047, USA

[§]These authors contributed equally

*Corresponding author. Email: mrivera@lsu.edu

ORCID: 0000-0002-5692-5497

Table of Contents

EXPERIMENTAL METHODS **S4**

Chemicals, Growth Media, and Bacterial Strains. S4

Crystallization and Data Collection. S4

Structure Solution and Refinement. S5

Determination of Compound Aqueous Solubility S5

Measurement of Half Maximal Inhibitory Concentration (IC_{50}). S6

Measurement of Minimum Inhibitory Concentrations (MIC). S6

Checkerboard Assay. S7

Time Kill Assays. S7

Determination of Compound Intracellular Accumulation. S8

Determination of pyoverdin secreted by *P. aeruginosa* PAO1 .... S9

Determination of Siderophores Secreted by *A. baumannii*. S9

Measurement of Dissociation Constant (K_d) S10

Biofilm Assays. S11

Scanning Electron Microscopy. S11

Synthesis and Characterization of Inhibitors. S12
General Synthetic Methods. S12
Method A: General Procedure for Reductive Amination using $NaBH(OAc)_3$. S13
Compounds in Table S1. .... S13
Method B: General Procedure Reductive Amination Mediated by $Ti(OiPr)_4$.[20] S14
Method C: General Procedure Reductive Amination Mediated by Ti(OiPr)4.[20] S15
Compounds in Table S1 .... S15
Compounds in Table S2. .... S23
Compounds in Table S3. .... S38
Compounds in Table S4. .... S51
^1H and ^{13}C spectra for 4-((5-Chloro-2-hydroxybenzyl)amino)isoindoline-1,3-dione (KM-5-25) S60
^1H and ^{13}C spectra for 4-((5-Bromo-2-hydroxybenzyl)amino)isoindoline-1,3-dione (KM-5-35) S61

SUPPLEMENTARY FIGURES **S62**

Figure S1. 4-Aminoisoindoline derivatives elicit a growth defect in planktonic *P. aeruginosa* and *A. baumannii* S62

Figure S2. *P. aeruginosa* (PAO1) cells in mature biofilms are susceptible to 4-amino-isoindole-1,3-dione analogues. S63

Figure S3. Checkerboard microdilution assay between KM-5-25 and (A) colistin, (B) imipenem against *A. baumannii* 5075. .... S63

SUPPLEMENTARY TABLES **S64**

Table S1. Analogues with an invariant 2-OH and one additional substituent in the aryl ring of the pharmacophore S64

Table S2. Analogues with an invariant 2-OH and two additional substituents in the phenyl ring of the pharmacophore S66

Table S3. Analogues with modification in the phthalimide bicycle of the pharmacophore S67

Table S4. Analogues with a heterocycle in place of the phenyl ring of the pharmacophore S68

Table S5. Crystallographic data for the Pa Bfr inhibitor complexes S69

REFERENCES S70

EXPERIMENTAL METHODS

Chemicals, Growth Media, and Bacterial Strains. Chemicals were purchased from Fisher Scientific unless otherwise specified. *P. aeruginosa* PAO1 was obtained from the University of Washington Genome center.[1] The PAO1 strain expressing enhanced yellow fluorescent protein (EYFP) was prepared previously.[2] *Acinetobacter baumannii* strains ATCC 17978 and ATCC 19606 were purchased from The American Type Culture Collection (ATCC) and *A. baumannii* strain 5075 was purchased from the University of Washington. MIC, IC_{50}, and checkerboard experiments were conducted in M63 media (2 g/L $(NH_4)SO_4$, 13.6 g/L KH_2PO_4, 2 g/L glucose (Acros Organics), 4 g/L citric acid, 0.24 g/L $MgSO_4$ (Sigma-Aldrich), 10% (v/v) non-essential amino acids (Gibco), 4% (v/v) essential amino acids (Gibco), 4 μM $(NH_4)_2Fe(SO_4)_2$ for *P. aeruginosa*, or 2.5 μM $(NH_4)_2Fe(SO_4)_2$ for *A. baumannii*, and 0.025% (w/v) hypromellose (HPMC, Sigma Aldrich), pH 7.0. The media was filter-sterilized by passing through a 0.2 μm cellulose acetate membrane syringe filter (VWR). AB minimal media: 2 g/L $(NH_4)_2SO_4$, 6 g/L Na_2HPO_4, 3 g/L KH_2PO_4, 3 g/L NaCl, 0.01 g/L Na_2SO_4, 0.4 g/L $MgCl_2$, 0.01 g/L $CaCl_2$. The media was prepared and autoclaved as previously reported[3] and prior to use was supplemented with filtered sterilized glucose (3 mM final concentration), $(NH_4)_2Fe(SO_4)_2$ (20 μM final concentration), and trace metals 3 μM $CuSO_4$, 7.6 μM $ZnSO_4$, 2 μM $Co(NO_3)_2$, 0.15 μM $(NH_4)_2MoO_4$, 9.4 μM $Na_2B_4O_7$ (all are final concentrations from individual autoclaved stocks).

Starter cultures of *P. aeruginosa* or *A. baumannii* strains in 5 mL of LB media were grown for 13 h and 17 h, respectively, in 50 mL conical tubes at 37 °C (*P. aeruginosa*), or 35 °C (*A. baumannii*) and 220 rpm. For biofilm experiments, the EYFP expressing *P. aeruginosa* strain was grown in *Pseudomonas* isolation (PI) media (20 g/ L peptone, 0.3 g/L $MgCl_2 \cdot 6H_2O$, 10 g/L K_2SO_4, 25 mg/L irgasan, and 20 mL/L glycerol, pH 7.0). Pellicle biofilms of *P. aeruginosa* PAO1 were cultured for 27 h at 30 °C in PI media supplemented with 20 μM Fe. Iron supplementation of media was carried out by addition of a small volume of filter-sterilized 10 mM $(NH_4)_2Fe(SO_4)_2$ (pH ~2.0) solution.

Crystallization and Data Collection. Crystallization screening of WT holo BfrB was conducted in Compact 300 (Rigaku Reagents) sitting drop vapor diffusion plates at 18 °C using equal volumes of crystallization solution

equilibrated against 75 µL of the latter. Crystals displaying a prismatic morphology were obtained in 1-2 days from the Cryo 1-2 HT screen (Rigaku Reagents) condition H6 (30% (v/v) PEG 200, 100 mM Na acetate pH 4.5, 100 mM NaCl). To prepare the ligand complexes, a multicomponent mixture[4] composed of 25% (v/v) dioxane, 25% (v/v) glycerol, 25% (v/v) diethylene glycol and 25% (v/v) ethylene glycol (SM3) was used to improve the ligand solubility. The ligand stock solution (100 mM in DMSO) was mixed in a 1:1 (v/v) ratio with SM3, and 4 µL of the ligand in SM3, 5 µL of 2 x crystallant and 1 µL of buffer were mixed to give a 20 mM ligand solution. Crystals were transferred to the soaking solution and incubated for 3 h before harvesting directly from the drop and storing in liquid nitrogen. X-ray diffraction data for the **KM-5-25** and **KM-5-35** complexes were collected at the Advanced Photon Source beamline 17-ID (IMCA-CAT) and the National Synchrotron Light Source-II (NSLS-II) NYX beamline 19-ID, respectively.

Structure Solution and Refinement. Intensities were integrated using XDS[5] via Autoproc[6] and the Laue class analysis and data scaling were performed with Aimless.[7] Structure solution was conducted by molecular replacement using the previously determined structure of *P. aeruginosa* BfrB (PDB: 5D8O)[8] as the search model with Phaser.[9] Structure refinement and manual model building were conducted with Phenix[10] and Coot respectively.[11] Structure validation was conducted with Molprobity.[12] Crystallographic data are provided in Table S5.

Determination of Compound Aqueous Solubility. Compound stocks (100 mM in DMSO) were prepared from powder and then further diluted with DMSO to prepare 20 mM working solutions, which were diluted as needed in DMSO. 15 µL of the DMSO solution was then diluted to a final volume of 1.0 mL in M63 media. Six solutions were prepared for each compound, each with a distinct concentration and 1.5% DMSO (v/v). 700 µL of each compound solution was transferred to a polystyrene 48-well plate (VWR), followed by acquisition of UV-vis spectra (300 to 700 nm) with the aid of an Epoch 2 microplate reader (BioTek). The plate was covered with an aeraseal air permeable membrane (Excel Scientific), placed in a shaker incubator (220 rpm) equilibrated at 37 °C for 4 h, and then transferred to the microplate reader to collect UV-vis spectra after removal of the aeraseal membrane. Following the acquisition of UV-vis spectra, each of the wells in the plate was inspected under a

microscope for evidence of precipitation. Evidence of precipitation at any given concentration was determined by observing a shift in the baseline of the UV-vis spectra relative to the blank (M63 media) and/or evidence of visual precipitation with the aid of a microscope.

Measurement of Half Maximal Inhibitory Concentration (IC_{50}). IC_{50} values were determined as reported earlier[13] with some modifications. Compound stock solutions in DMSO (100 mM and 10 mM) were prepared from powder. To prepare the cell suspensions utilized in the measurements, starter cultures of *P. aeruginosa* were diluted to an optical density at 600 nm (OD_{600}) = 0.005 in fresh LB media (5 mL) and then cultured at 37 °C and 220 rpm for 4 h prior to diluting to an OD_{600} = 0.0001 in M63. A small volume of compound stock solution was further diluted in DMSO to 30 μL and then diluted in M63 cell suspension to a final volume of 2.0 mL. The resultant solution was placed in a well of a 96-well plate and then serially 2-fold diluted in M63 cell suspension to produce 6 distinct compound concentrations, each with 1.5% DMSO. The plate was covered and incubated for 18 h at 35 °C in a Synergy H1 plate reader (Biotek). The resultant cell cultures were serially diluted in PBS and then plated on PI Agar plates (PIA; BD biosciences) for counting viable cells (CFU/mL). The % growth was calculated from the ratio $\text{CFU/mL}_{(\text{treated})}/\text{CFU/mL}_{(\text{untreated control})}$. To calculate the IC_{50} values, the % growth was plotted as a function of compound concentration expressed as *log*[compound] (μM), and fitted to equation S1 describing the sigmoid-shaped response pattern,[14] where *b* is the slope factor, *max* is the upper asymptote (plateau), and *min* is the lower asymptote. IC_{50} values are the average and standard deviation from three independent experiments, each conducted with three technical replicates.

$$\%\, growth = min + \frac{max - min}{1 + 10^{(log\mathrm{IC}_{50} - x)\cdot b}} \qquad \text{(S1)}$$

Measurement of Minimum Inhibitory Concentrations (MIC). The MIC of compounds and selected antibiotics against three strains of *A. baumannii* (5075, ATCC 17988, and ATCC 19606) were determined via the broth microdilution method according to Clinical and Laboratory Standard Institute (CLSI) guidelines,[15] but culturing the cells in M63 media. In brief, a starter culture was diluted in M63 media to ~ 5 x 10^5 CFU/mL for the MIC test. To establish a 2-fold dilution series of compound, 985 μL of diluted cells was mixed with 15 μL of compound

dissolved in DMSO, such that the resultant solution had the highest concentration of compound or antibiotic tested and 1.5% DMSO. To carry out the serial dilution from the highest compound concentration, a 150 µL solution of higher concentration compound or antibiotic was mixed with 150 µL of cell suspension containing 1.5% DMSO in the wells of a 96 well plate. Controls were included where the cell suspension in the wells did not include inhibitor or antibiotic, or did not include bacteria. The 96 well plate was incubated for 20 h at 35 °C with continuous orbital shaking (205 cpm) in an Epoch2 microplate reader (BioTek), monitoring the growth by following the OD_{600}. The MIC values were defined as the lowest concentration of compound or antibiotic that showed no visible growth. All assays were performed in three biological replicates, each with three technical replicates.

Checkerboard Assay. A 17 h starter culture of *A. baumannii* 5075 was diluted in M63 media to ~ 5 x 10^5 CFU/mL. Concentration gradients of **KM-5-35** or **KM-5-25** and select antibiotics in M63 media were prepared in the vertical and horizontal direction, respectively. Bacterial suspensions were prepared as described above for the MIC determination and added to the plates. Controls were included where the solution in the wells did not contain inhibitors or antibiotics, or did not include bacteria. The 96-well plates were incubated for 20 h at 35 °C with continuous orbital shaking (205 cpm) in an Epoch2 microplate reader (BioTek), monitoring the growth by following the OD_{600}. The MIC values of inhibitor and antibiotic were obtained from the corresponding row or column which contained only one component, whereas the remaining rows and columns were utilized for calculating the fractional inhibitory concentration index (FIC) with the aid of equation S2.

$$FIC = \frac{MIC_{A\ in\ combination}}{MIC_A} + \frac{MIC_{B\ in\ combination}}{MIC_B} \qquad \text{S2}$$

Time Kill Assays. Time-kill assays were performed with *A. baumannii* 5075 by the broth macro-dilution method in M63-medium containing 1.5% DMSO. An overnight culture was diluted to approximately 5 x10^5 CFU/mL in fresh M63 media. The test solutions were prepared by adding compound (**KM-5-35**, or **KM-5-25**) dissolved in DMSO to 5 mL of diluted cell suspension to yield a solution of the desired compound concentration and 1.5% DMSO, or by the addition of antibiotic dissolved in water and a small volume of DMSO to produce the desired

antibiotic concentration and 1.5% DMSO. Controls containing the diluted cell suspension, 1.5% DMSO but no test compounds were also prepared. The cell suspensions were incubated in an orbital shaker (200 rpm, 35 °C) and 100 µL aliquots were removed from the culture at 0, 0.5, 1, 2, 3, 5 and 24 h post inoculation, and subjected to serial dilution and plating (50 µL) on LB agar plates for CFU/mL determination after incubation at 35 °C for 24 h.

Determination of Compound Intracellular Accumulation. These experiments were carried out as reported previously,[16] with modifications. Starter cultures of *P. aeruginosa* PAO1 or *A. baumannii* 5075 were centrifuged for 10 min at 3148 x g and 4 °C, resuspended in 5 mL of fresh LB, diluted to OD_{600} = 0.03 in fresh LB media (250 mL), and then cultured at 35 °C and 220 rpm for 4 h. The resultant culture was diluted to OD_{600} = 0.4 in 250 mL LB and centrifuged at 4 °C and 3470 x g for 10 min. The cell pellet was resuspended in 20 mL of PBS and transferred to a 50 mL conical tube, where it was centrifuged at 4 °C and 3148 x g for 20 min. The cell pellet was resuspended in 15 mL of PBS supplemented with 0.05% (w/v) HPMC and the suspension was divided equally into two new conical tubes. One of the tubes was treated with 150 µL of DMSO (control), while the second tube was treated with 150 µL of compound in DMSO solution to produce a compound final concentration of 30 µM or 15 µM. The resultant mixtures were incubated at 37 °C and 220 rpm for 15 min, placed on ice for 1 min, and then centrifuged at 3148 x g rpm and 4 °C for 10 min. The cell pellet was resuspended in 7.5 mL of ice-cold PBS and vortexed (10 s, five times). 200 µL of the cell suspension was used to plate and enumerate viable cells, and 800 µL of the suspension was layered onto 700 µL of chilled silicon oil contained in 1.5 mL Eppendorf tubes and then centrifuged at 4 °C (900 x g) for 10 min, then again at 16,200 x g for 5 min to allow passage of the cell suspension through the silicon oil layer. The aqueous and oil layers were removed, and the cell pellet was washed twice in PBS (800 µL), each time transferring the cell suspension to a new Eppendorf tube and then centrifuging at 16,200 x g. The cell pellet was lysed in 800 µL methanol by vortexing followed by 10 min incubation at room temperature. The lysate suspension was pelleted, the supernatant was transferred to a new Eppendorf tube and then placed into a vacuum concentrator (Eppendorf 5301 Vacufuge) to evaporate the methanol (4 h). The dried

cell lysate was resuspended in 600 μL of DMSO and centrifuged at 16,200 x g rpm for 5 min at room temperature. The clarified supernatant was titrated with small volumes of 250 μM compound stock, measuring the fluorescence after the addition of each aliquot with the aid of a Cary Eclipse Fluorescence Spectrometer (Agilent). The compound concentration was determined utilizing the standard addition method,[17] and compound accumulation is reported as nmol compound/10^{12} CFU/mL.

Determination of pyoverdin secreted by *P. aeruginosa* PAO1. These experiments were conducted as previously reported[13] with some modifications. Cells were cultured in modified M63 media (2 g/L $(NH_4)_2SO_4$, 13.6 g/L KH_2PO_4 (Sigma Aldrich), 2 g/L glucose, 4 g/L citric acid, 5 g/L technical grade casamino acids (BD scientific), 0.24 g/L $MgSO_4$ (Alfa Aesar), and 0.025% (w/v) HPMC, pH 7.0 adjusted with KOH). Starter cultures in LB were diluted to OD_{600} = 0.0001 and cultured in a shaker incubator (35 °C and 205 rpm) for 24 h, in the absence (control) and in the presence of **KM-5-25** (80 μM), **KM-5-35** (60 μM), **EB-5-73** (40 μM), and **SB-17-140** (80 μM); all cell suspensions have 1.5% DMSO. 700 μL of the culture were clarified by centrifugation (16,200 x g, 10 min, 4 °C), diluted 10-fold in PBS and then analyzed by acquiring fluorescence emission spectra (410-600 nm) with excitation at 400 nm (10 nm slit width) and emission at λ_{max}= 430 nm (10 nm slit width) using an Agilent (Cary Eclipse) fluorescence spectrophotometer. The fluorescence intensity of the treated sample $(FI)_{Tr}$ and that of the untreated control $(FI)_{Un}$ was normalized to the corresponding OD_{600} and the ratio $(FI/OD_{600})_{Tr}/(FI/\ OD_{600})_{Un}$ is reported in Figure 8A. The determination for each of the compounds was carried out in 5 biological replicates.

Determination of Siderophores Secreted by *A. baumannii.* The chromo azurol S (CAS) solution was prepared as previously reported[18] with some modifications. The blue dye solution (0.6 g/L CAS (ACROS Organics), 100 μM $FeCl_3 \cdot 6H_2O$, and 0.73 g/L hexadecyltrimethylammonium bromide (HDTMA, ACROS Organics) was autoclaved and stored in the dark. The MM9-PIPES buffer solution (3 g/L KH_2PO_4, 5 g/L NaCl, 10 g/L NH_4Cl, 32.24 g/L piperazine-1,4-bis-2-ethanesulfonic acid (PIPES, Sigma Aldrich), pH 6.75) was sterilized using a 0.22 μm syringe filter (VWR). Prior to every experiment, blue dye solution (0.5 mL) was mixed with 4.5 mL of MM9-PIPES solution. An overnight culture of *A. baumannii* 5075 was diluted to OD_{600} = 0.0001 with modified M63 media (no citric acid) supplemented with 4 μM Fe and transferred to a 96-well plate, where it was cultured for 20

h at 35 °C and 205 cpm in a plate reader (Synergy H1, BioTek). The treated cell cultures contained compound at the concentrations indicated in the caption of Figure 8B and 1.0% DMSO, whereas the untreated control contained 0.1% DMSO. 700 µL was centrifuged at 16,200 x g for 10 min. 200 µL of the clarified supernatant was mixed with the 200 µL of CAS solution and allowed to incubate at room temperature in the dark for 2 h prior to recording a UV-Vis spectrum (200 nm to 800 nm) in a 1.0 cm quartz cuvette with the aid of a Cary 60 UV-Vis spectrophotometer (Agilent Technologies). The fold-change in siderophore secreted by the treated vs. untreated cells was calculated using equation S3, where Tr indicates treated cells, Un indicates DMSO control cells, $(\Delta A_{630})_{Tr} = (A_{630})_{Tr} - (A_{630})$, $(\Delta A_{630})_{Un} = (A_{630})_{Un} - (A_{630})$, and A_{630} indicates the absorbance at 630 nm from a solution of fresh modified M63 media. The experiment was carried out in at least five biological replicates for each of the compounds reported in Figure 8B.

$$Fold\ change = \frac{\frac{(\Delta A_{630})Tr}{(OD_{600})Tr}}{\frac{(\Delta A_{630})Un}{(OD_{600})Un}} \qquad \text{(S3)}$$

Measurement of Dissociation Constant (K_d) Recombinant Pa BfrB was expressed in *E. coli* and purified as previously reported.[19] Apo-BfrB (devoid of heme) was utilized for the measurements to prevent heme-driven fluorescence quenching. A 0.5 mL solution containing 0.01 µM apo Pa-BfrB in PBS (pH = 7.4) containing 0.5% DMSO was placed into a 1 cm quartz cuvette where it was titrated with aliquots of compound dissolved in a 50% (v/v) PBS/DMSO solution. The fluorescence quenching brought by the addition of each aliquot was monitored with the aid of an Agilent Cary Fluorometer, with excitation at 280 nm, emission at 320-470 nm, 10 x10 nm slit width, and an average of 5 scans per spectrum. The binding assay included at least 8 aliquots spanning from ~ 0.1 to 4-8 times the K_d, depending on compound solubility. A control titration was carried out by titrating compound into PBS buffer. The K_d was calculated by fitting the data to equation S4, where F_b is the fraction bound and F_{max} is the fraction bound at at saturating compound concentration.

$$F_b = F_{max}\left[\frac{[compound]}{K_d + [compound]}\right] \qquad \text{(S4)}$$

Biofilm Assays. Pellicle biofilms formed by EYFP-expressing *P. aeruginosa* PAO1 were carried out as reported previously,[13] with some modifications. Starter cultures were diluted to OD_{600} = 0.001 in 4 mL PI media supplemented with 20 μM Fe, transferred to petri dishes (35 x 10 mm) and incubated statically for 27 h at 30 °C. The pellicles were transferred onto circular microscope glass coverslips (12 mm diameter) by gently allowing the surface of a coverslip to contact the pellicle. The biofilm-attached coverslip was washed (3 x) by floating it on top of PBS with the biofilm exposed to the liquid and then placed on top of 1.5 mL AB challenge media in a well of a 12-well microplate, with the biofilm exposed to the media at 30 °C for 24 h. AB challenge media is AB minimal media supplemented with 1.5% DMSO, 0.025% HPMC, 20 μM Fe, and 4-aminoisoindoline-1,3-dione derivative used at the concentrations indicated in Tables S1-S5. The 4-aminoisoindoline-1,3-dione derivative solutions were prepared from powder as 10 mM stock solutions in DMSO and then diluted in AB challenge media to the appropriate concentrations. Following the 24 h challenge, planktonic and loosely attached cells were washed 3 times by floating the coverslip-attached biofilms with the biofilm facing down into a petri dish (60 x 15 mm) containing 15 mL PBS, and incubating for 5 min. The biofilm was removed from the coverslip and the cells released from the extracellular matrix by placing the coverslip-attached biofilm in a 50 mL conical tube containing a 2-mL supension of zirconia beads (0.1 mm diameter, BiosSpec Products), 10 mL PBS, 1 mM $MgSO_4$, 20 μg/mL alginate lyase and 40 μg/mL DNAse. The mixture was incubated for three min at room temperature, and then vigorously vortexed for four min. Following sedimentation of the zirconia beads, a 100 μL aliquot was utilized for serial dilution in PBS and plating on PIA plates for viable cell enumeration (CFU/mL).

Scanning Electron Microscopy. Pellicle biofilms formed by EYFP-expressing *P. aeruginosa* PAO1 were cultured, harvested, and treated with 50 μM **KM-5-35**, or **KM-5-25**, as described in Biofilm Assays. The resultant biofilms were immersed in a solution containing 2.5% glutaraldehyde, 2% paraformaldehyde in 0.1 M phosphate buffer (pH 7.2) at 4 °C, rinsed by immersion in distilled water for 10 min (3 times), then fixed in a solution containing 1% OsO_4 in 0.1 M phosphate buffer (pH 7.2) for 30 min at room temperature, and rinsed by immersion in distilled water for 10 min (3 times), followed by dehydration in a graded ethanol series (10 min per change). The ethanol was replaced with a graded hexamethyldisilazane (HDMS; 50%, 70% and 100%, 10 min each

change), the HMDS was removed, and the samples were air-dried overnight. The samples were mounted on aluminum specimen stubs, coated with platinum in an EMS550X Sputter Coater and imaged with the aid of FEO Quanta 3D FEG Ga FIB/SEM.

Synthesis and Characterization of Inhibitors.

General Synthetic Methods. All chemicals and anhydrous solvents were purchased from commercially available sources and used without additional purification. General solvents and reagents were purchased from Fisher Scientific or Combi Blocks. ^{1}H NMR spectra were obtained on a Varian Mercury 400-MHz NMR, Bruker Avance 400-MHz NMR or 300 Varian Mercury 300-MHz NMR. Chemical shifts were reported in parts per million (ppm, δ) using various solvents as internal standards ($CDCl_3$, δ 7.26 ppm; DMSO-d_6, δ 2.49 ppm). ^{1}H-NMR splitting patterns were designated as singlet (s), doublet (d), triplet (t), or quartet (q). Splitting patterns that could not be interpreted or easily visualized were recorded as multiplets (m) or broad (br). Coupling constants (*J*) are reported in Hertz (Hz).

High resolution mass spectrometry (HRMS) measurements were conducted on a Waters Synapt XS ESI-Q-IMS-TOF mass spectrometer coupled to a Waters Acquity Premiere UPLC system. Samples were run with a capillary voltage of 1.5 kV in positive mode. The mass range monitored was 50-1200 m/z, while lockmass calibration was acquired with a Leu-Enk solution sprayed at 10 s intervals with a secondary sprayer orthogonal to the main sample sprayer. The system was operated in MS mode, with an acquisition time of 0.3 s per mass scan event. A Waters Premier BEH C18 column (50 x 2.7 mm, 1.7 µm particle size, 130 Å pore size) was used for chromatographic separation with a gradient program using a binary mixture of mobile phases at a fixed flow rate of 400 µL/min. Mobile phases were as follows: A= H_2O with 0.1% formic acid, and B = acetonitrile with 0.1% formic acid. The gradient program was as follows: 0-1 min = 5% B, 1-5 min 95% B, 5-6.5 min 95% B, 6.5-7 min 5% B, 7-10 min, 5% B. Compounds dissolved in DMSO (10 mg/mL) were diluted 10-fold in DMSO and further diluted in mobile phase (80:20 acetonitrile:0.1% aqueous formic acid) to 15 µg/mL. The injection volume was 1 µL. Data analysis was carried out with Waters MassLynx (Ver. 4.2). Blank runs were used to exclude peaks from integration. HRMS

data is reported for compounds for which biological data (IC_{50}, MIC, % biofilm survival, and intracellular accumulation) is available in Tables 2-4 and Table S1. These compounds are >95% pure.

Method A: General Procedure for Reductive Amination using $NaBH(OAc)_3$.

To a solution of the aldehyde (2.0 equivalents) in DMF (5 mL) was added AcOH (3 mL) and the solution was stirred for 10 min. To this reaction mixture, 4-amino-isoindole-1,3-dione (1.0 equivalent) was added and stirring was continued at 23 °C for 1 h. The reaction was cooled to 10 °C and sodium triacetoxyborane ($NaBH(OAc)_3$, 3.0 equivalents) was added portion-wise to the reaction, and the mixture was stirred for 30 min. The reaction was gradually warmed to 23 °C and stirred for 18 h or until TLC indicated the reaction was complete. The crude reaction mixture was poured into de-ionized water, extracted with EtOAc (3 × 75 mL) and the combined organic layers were washed with aq $NaHCO_3$ (2 × 50 mL) and aq NaCl (50 mL). The organic layer was dried (Na_2SO_4) and concentrated under vacuum. The crude amine was purified by column chromatography on silica gel (pre-treated with 3-hydroxy-2-methyl-4-pyrone, 100 mg/2 kg of silica in 4 L of methanol, filtered, washed with methanol, and dried), or crystallization from ethanol, or both, to afford the pure product.

Compounds in Table S1.

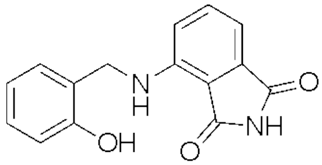

Analog 11.

4-((2-Hydroxybenzyl)amino)isoindoline-1,3-dione (**Analog 11**). Previously described in Punchi-Hewage, A.; Yao, H.; Nammalwar, B.; Gnanasekaran, K. K.; Lovell, S.; Bunce, R. A.; Eshelman, K.; Phaniraj, S. M.; Peterson, B. R.; Battaile, K. P.; Reitz, A. B.; Rivera, M. Small Molecule Inhibitors of the BfrB:Bfd Interaction Decrease *P. aeroginosa* Fitness and Potentiate Fluoroquinolone Activity. *J. Am. Chem. Soc.*, **2019**, *141*, 8171-8184. https://doi.org/10.1021/jacs.9b00394

HRMS $[M+H]^+$ calculated for $C_{15}H_{12}N_2O_3$: 269.0926; found: 269.0930, err. = 1.5 ppm.

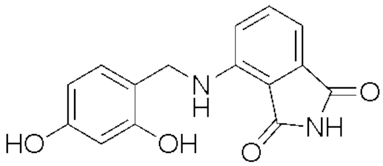

BN-16-69

4-((2,4-Dihydroxybenzyl)amino)isoindoline-1,3-dione (**BN-16-69**). Purified by silica gel chromatography using increasing concentrations of ethyl acetate (5-25%) in hexanes and recrystallization from ethanol to give a yellow solid, 32% yield; ^{1}H NMR (400 MHz, DMSO-d_6): δ 10.92 (s, 1H), 9.55 (s, 1H), 9.18 (s, 1H), 7.49 (t, *J* = 7.8 Hz, 1H), 7.05 (d, *J* = 8.5 Hz, 1H), 6.99 (d, *J* = 8.2 Hz, 1H), 6.91 (d, *J* = 7.1 Hz, 1H), 6.77 (t, *J* = 6.0 Hz, 1H), 6.32 (br s, 1H), 6.16 (br d, *J* = 8.0 Hz,1H), 4.29 (d, *J* = 6.0 Hz, 2H).

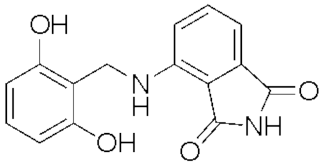

BN-16-70

4-((2,6-Dihydroxybenzyl)amino)isoindoline-1,3-dione (**BN-16-70**). Purified by silica gel chromatography using increasing concentrations of ethyl acetate (5-25%) in hexanes and recrystallization from ethanol to give a yellow solid, 28% yield; ^{1}H NMR (400 MHz, DMSO-d_6): δ 10.90 (s, 1H), 9.62 (s, 2H), 7.52 (t, *J* = 7.9 Hz, 1H), 7.29 (d, *J* = 8.5 Hz, 1H), 6.91-6.84 (complex, 2H), 6.77 (t, *J* = 6.3 Hz, 1H), 6.31 (d, *J* = 8.1 Hz, 2H), 4.37 (d, *J* = 6.3 Hz, 2H).

Method B: General Procedure Reductive Amination Mediated by $Ti(OiPr)_4$.[20]

To a mixture of 4-amino-isoindole-1,3-dione (1.0 equivalent) and aldehyde (2.0 equivalents) in DMF was added titanium(IV) isopropoxide ($Ti(OiPr)_4$, 2.0 equivalents). The reaction was stirred at 20 °C for 5 hours, then was cooled to 0 °C. To the reaction was added sodium borohydride ($NaBH_4$, 1.0 equivalent), and the reaction was allowed to warm to 20 °C. The reaction was stirred for the stated time or until TLC indicated reaction was complete, and then an aqueous work-up was carried out, followed by purification, to give the *N*-alkylated 4-amino-isoindole-1,3-dione. Unless otherwise stated, aqueous workup was carried out by pouring the crude reaction mixture into de-ionized water and then extracting with EtOAc (3 × 75 mL). The combined organic layers

were washed with aq $NaHCO_3$ (2 × 50 mL) and saturated aqueous NaCl (brine) (50 mL). The organic layer was dried (Na_2SO_4) and concentrated under vacuum. Unless otherwise stated, the crude amine was purified by column chromatography on silica gel (pre-treated with 3-hydroxy-2-methyl-4-pyrone, 100 mg/2 kg of silica in 4 L of methanol, filtered, washed with methanol, and dried) or recrystallization from ethanol or both to afford the pure products.

Method C: General Procedure Reductive Amination Mediated by Ti(OiPr)4.[20]

Similar to Method B, except the imine formation was conducted at 80 °C.

Compounds in Table S1

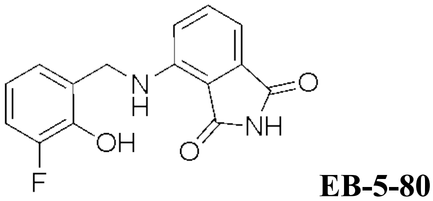

EB-5-80

4-((3-Fluoro-2-hydroxybenzyl)amino)isoindoline-1,3-dione (**EB-5-80**). Purified by silica gel chromatography using increasing concentrations of ethyl acetate (5-25%) in hexanes to give a yellow solid, 72% yield; ^{1}H NMR (400 MHz, DMSO-d_6): δ 11.0 (s, 1H), 9.85 (s, 1H), 7.48 (t, J = 8.4 Hz, 1H), 7.10-6.99 (complex, 3H), 6.96 (d, J = 8.4 Hz, 1H), 6.93 (d, J = 7.0 Hz, 1H), 6.76 (dt, J = 5.0 7.9 Hz, 1H), 4.50 (d, J = 6.3 Hz, 2H); HRMS $[M+H]^+$ calculated for $C_{15}H_{11}FN_2O_3$: 287.0832; found: 287.0827, err. = -1.7 ppm.

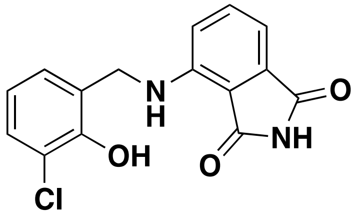

KM-5-30

4-((3-Chloro-2-hydroxybenzyl)amino)isoindoline-1,3-dione (**KM-5-30**). Previously described in: Soldano, A.; Yao, H.; Punchi Hewage, A. N. D.; Meraz, K.; Annor-Gyamfi, J. K.; Bunce, R. A.; Battaile, K. P.; Lovell, S.; Rivera, M. Small Molecule Inhibitors of the Bacterioferritin (BfrB)–Ferredoxin (Bfd) Complex Kill Biofilm-Embedded *Pseudomonas aeruginosa* Cells. *ACS Infect. Dis.* **2021**, *7*, 123-140; PMID: 33269912.

https://doi.org/10.1021/acsinfecdis.0c00669 HRMS $[M+H]^+$ calculated for $C_{15}H_{11}ClN_2O_3$: 303.0536; found: 303.0531, err. = -1.6 ppm.

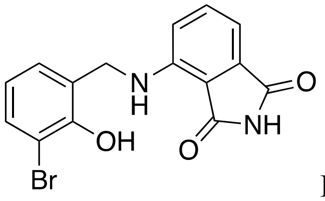

EB-5-73

4-((3-Bromo-2-hydroxybenzyl)amino)isoindoline-1,3-dione (**EB-5-73**). Purified by silica gel chromatography using increasing concentrations of ethyl acetate (5-25%) in hexanes to give a yellow solid, 67% yield; ^{1}H NMR (400 MHz, $CDCl_3$): δ 10.98 (br s, 1H), 9.43 (s, 1H), 7.48 (dd, J = 7.1, 8.5 Hz, 1H), 7.42 (dd, J = 1.6, 8.0 Hz, 1H), 7.23 (dd, J = 1.6, 7.7 Hz, 1H), 7.09 (br t, J = 6.3 Hz, 1H), 6.94 (d, J = 7.1 Hz, 1H), 6.92 (d, J = 8.5 Hz, 1H), 6.76 (t, J = 7.7 Hz, 1H), 4.53 (d, J = 6.3 Hz, 2H); HRMS $[M+H]^+$ calculated for $C_{15}H_{11}BrN_2O_3$: 347.0031; found: 347.0040, err. = 2.6 ppm.

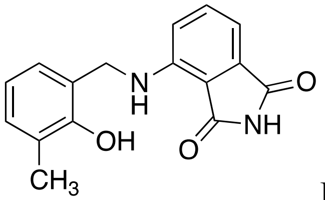

EB-5-70

4-((2-Hydroxy-3-methylbenzyl)amino)isoindoline-1,3-dione (**EB-5-70**). Purified by silica gel chromatography using increasing concentrations of ethyl acetate (5-25%) in hexanes to give a yellow solid, 75% yield; ^{1}H NMR (400 MHz, $CDCl_3$): δ 10.95 (br s, 1H), 8.64 (s, 1H), 7.48 (dd, J = 7.1, 8.5, Hz, 1H), 7.06 (dd, J = 1.7, 7.7 Hz, 1H), 7.02-6.96 (complex, 3H), 6.91 (d, J = 7.1 Hz, 1H), 6.69 (t, J = 7.5 Hz, 1H), 4.47 (d, J = 6.2 Hz, 2H), 2.19 (s, 3H); HRMS $[M+H]^+$ calculated for $C_{16}H_{14}N_2O_3$: 283.1083; found: 283.1079, err. = -1.4 ppm.

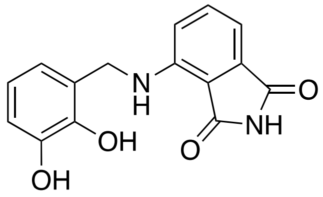

BN-4-74

4-((2,3-Dihydroxybenzyl)amino)isoindoline-1,3-dione (**BN-14-74**). Purified by silica gel chromatography using increasing concentrations of ethyl acetate (5-25%) in hexanes top give a yellow solid, 30% yield; ^{1}H NMR (400 MHz, DMSO-d_6): δ 10.94 (s, 1H), 9.34 (s, 1H), 8.60 (s, 1H), 7.48 (t, J = 7.8 Hz, 1H), 6.99 (d, J = 8.5 Hz, 1 H), 6.91 (m, 2H), 6.69 (t, J = 7.2 Hz, 2H), 6.57 (t, J = 7.8 Hz, 1H), 4.42 (d, J = 6.1 Hz, 2H); HRMS [M+H]$^+$ calculated for $C_{15}H_{12}N_2O_4$: 285.0875; found: 285.0880, err. = 1.8 ppm.

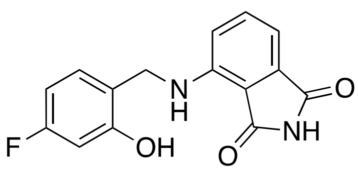

EB-5-62

4-((4-Fluoro-2-hydroxybenzyl)amino)isoindoline-1,3-dione (**EB-05-62**). Purified by silica gel chromatography using increasing concentrations of ethyl acetate (5-25%) in hexanes to give a yellow solid, 66% yield; ^{1}H NMR (400 MHz, DMSO-d_6): δ 10.93 (s, 1H), 10.25 (s, 1H), 7.49 (t, J = 7.8 Hz, 1H), 7.23 (t, J = 7.8 Hz, 1H), 6.99 (d, J = 8.7 Hz, 1H), 6.95 (obscured, 1H), 6.92 (d, J = 7.1 Hz, 1H), 6.62 (dd, J = 2.6, 10.7 Hz, 1H), 6.57 (m, 1H), 4.40 (d, J = 6.3 Hz, 2H); HRMS [M+H]$^+$ calculated for $C_{15}H_{11}FN_2O_3$: 287.0832; found: 287.0832, err. = 0.0 ppm.

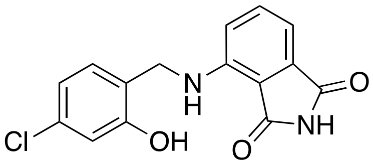

EB-5-63

4-((4-Chloro-2-hydroxybenzyl)amino)isoindoline-1,3-dione (**EB-05-63**). Purified by silica gel chromatography using increasing concentrations of ethyl acetate (5-25%) in hexanes to give a yellow solid, 69% yield; ^{1}H NMR (400 MHz, DMSO-d_6): δ 10.96 (s, 1H), 10.27 (s, 1H), 7.48 (t, J = 7.8 Hz, 1H), 7.20 (d, J = 8.1 Hz, 1H), 7.01 (t, J = 6.3 Hz, 1H), 6.96-6.91 (complex, 2H), 6.87 (d, J = 2.1 Hz, 1H), 6.80 (dd, J = 2.1, 8.2 Hz, 1H), 4.42 (d, J = 6.3 Hz, 2H); HRMS [M+H]$^+$ calculated for $C_{15}H_{11}ClN_2O_3$: 303.0536; found: 303.0541, err. = 1.6 ppm.

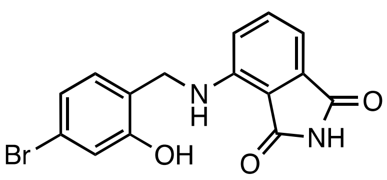

EB-5-61

4-((4-Bromo-2-hydroxybenzyl)amino)isoindoline-1,3-dione (**EB-5-61**). Purified by silica gel chromatography using increasing concentrations of ethyl acetate (5-25%) in hexanes to give a yellow solid, 62% yield; ^{1}H NMR (400 MHz, DMSO-d_6): δ 10.96 (s, 1H), 10.25 (s, 1H), 7.48 (dd, *J* = 7.1, 8.5 Hz, 1H), 7.14 (d, *J* = 8.1 Hz, 1H), 7.01 (t, *J* = 6.3 Hz, 1H), 7.00 (d, *J* = 2.0 Hz, 1H), 6.94 (m, 1H), 6.94-6.91 (complex, 3H), 4.40 (d, *J* = 6.3 Hz, 2H); HRMS $[M+H]^+$ calculated for $C_{15}H_{11}BrN_2O_3$: 347.0031; found: 347.0038, err. = 2.0 ppm.

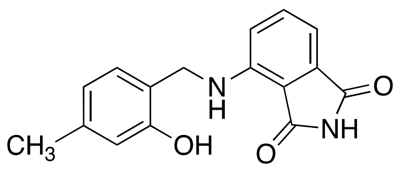

EB-5-69

4-((2-Hydroxy-4-methylbenzyl)amino)isoindoline-1,3-dione (**EB-5-69**). Purified by silica gel chromatography using increasing concentrations of ethyl acetate (5-25%) in hexanes to give a yellow solid, 71% yield; ^{1}H NMR (400 MHz, $CDCl_3$): δ 10.94 (br s, 1H), 9.59 (s, 1H), 7.48 (dd, *J* = 7.1, 8.5 Hz, 1H), 7.08 (d, *J* = 7.7 Hz, 1H), 7.00 (d, *J* = 8.5 Hz, 1H), 6.93-6.88 (complex, 2H), 6.65 (d, *J* = 1.7 Hz, 1H), 6.56 (dd, *J* = 1.7, 7.7 Hz, 1H), 4.38 (d, *J* = 6.1 Hz, 2H), 2.19 (s, 3H); HRMS $[M+H]^+$ calculated for $C_{16}H_{14}N_2O_3$: 283.1083; found: 283.1082, err. = -0.4 ppm.

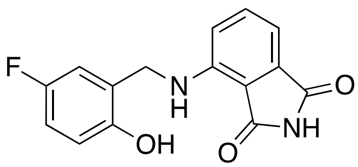

JAG-5-7

4-((5-Fluoro-2-hydroxybenzyl)amino)isoindoline-1,3-dione (**JAG-5-7**). Previously described in: Soldano, A.; Yao, H.; Punchi Hewage, A. N. D.; Meraz, K.; Annor-Gyamfi, J. K.; Bunce, R. A.; Battaile, K. P.; Lovell, S.;

Rivera, M. Small Molecule Inhibitors of the Bacterioferritin (BfrB)–Ferredoxin (Bfd) Complex Kill Biofilm-Embedded *Pseudomonas aeruginosa* Cells. *ACS Infect. Dis.* **2021**, *7*, 123-140; PMID: 33269912. https://doi.org/10.1021/acsinfecdis.0c00669 HRMS $[M+H]^+$ calculated for $C_{15}H_{11}FN_2O_3$: 287.0832; found: 287.0838, err. = 2.1 ppm.

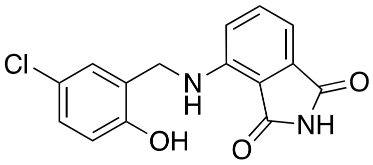

KM-5-25

4-((5-Chloro-2-hydroxybenzyl)amino)isoindoline-1,3-dione (**KM-5-25**). Previously described in: Soldano, A.; Yao, H.; Punchi Hewage, A. N. D.; Meraz, K.; Annor-Gyamfi, J. K.; Bunce, R. A.; Battaile, K. P.; Lovell, S.; Rivera, M. Small Molecule Inhibitors of the Bacterioferritin (BfrB)–Ferredoxin (Bfd) Complex Kill Biofilm-Embedded *Pseudomonas aeruginosa* Cells. *ACS Infect. Dis.* **2021**, *7*, 123-140; PMID: 33269912. https://doi.org/10.1021/acsinfecdis.0c00669. ^{13}C NMR (101 MHz, DMSO-d_6): δ 171.3, 169.3, 154.1, 145.9, 135.9, 133.6, 127.8, 127.7, 127.0, 122.4, 116.6, 111.3, 111.2, 110.2, 40.6; HRMS $[M+H]^+$ calculated for $C_{15}H_{11}ClN_2O_3$: 303.0536; found: 303.0533, err. = -1.0 ppm.

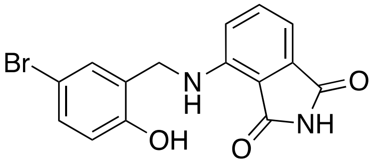

KM-5-35

4-((5-Bromo-2-hydroxybenzyl)amino)isoindoline-1,3-dione (**KM-5-35**). Purified by silica gel chromatography using increasing concentrations of ethyl acetate (5-25%) in hexanes to give a yellow solid, 65% yield; ^{1}H NMR (400 MHz, DMSO-d_6): δ 10.96 (s, 1H), 10.08 (s, 1H), 7.49 (t, J = 8.0 Hz, 1H), 7.35 (d, J = 2.5 Hz, 1H), 7.23 (dd, J = 2.5, 8.6 Hz, 1H), 7.06 (t, J = 6.4 Hz, 1H), 6.96 (d, J = 8.5 Hz, 1H), 6.93 (d, J = 7.1 Hz, 1H), 6.79 (d, J = 8.6 Hz, 1H), 4.41 (d, J = 6.4 Hz, 2H); ^{13}C NMR (101 MHz, DMSO-d_6): δ 171.8, 169.8, 155.0, 146.4, 136.3, 134.1,

131.1, 128.1, 117.7, 117.1, 111.7, 110.6, 110.5, 41.1 (one aromatic carbon unresolved); HRMS $[M+H]^+$ calculated for $C_{15}H_{11}BrN_2O_3$: 347.0031; found: 347.0031, err. = 0.0 ppm.

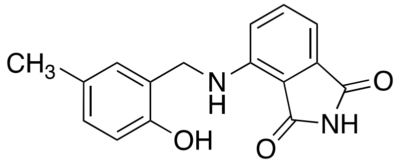

EB-5-1

4-((2-Hydroxy-5-methylbenzyl)amino)isoindoline-1,3-dione (**EB-05-1**). Purified by silica gel chromatography using increasing concentrations of ethyl acetate (5-25%) in hexanes to give a yellow solid, 70% yield; ^1H NMR (400 MHz, DMSO-d_6): δ 10.95 (s, 1H), 9.48 (s, 1H), 7.49 (t, *J* = 7.9 Hz, 1H), 7.03 (s, 1H), 7.03 (d, *J* = 8.1 Hz, 1H), 6.96-6.86 (complex, 3H), 6.74 (d, *J* = 8.1 Hz, 1H), 4.39 (d, *J* = 6.1 Hz, 2H), 2.15 (s, 3H); HRMS $[M+H]^+$ calculated for $C_{16}H_{14}N_2O_3$: 283.1083; found: 283.1085, err. = 0.7 ppm.

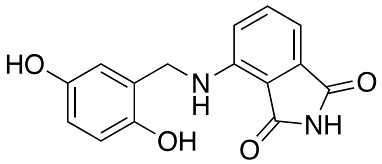

EB-5-93

4-((2,5-Dihydroxybenzyl)amino)isoindoline-1,3-dione (**EB-5-93**). Purified by silica gel chromatography using increasing concentrations of ethyl acetate (5-25%) in hexanes to give a yellow solid, 57% yield; ^1H NMR (400 MHz, DMSO-d_6): δ 10.95 (br s, 1H), 8.96 (s, 1H), 8.61 (s, 1H), 7.48 (dd, *J* = 7.0, 8.5 Hz, 1H), 6.97 (t, *J* = 6.3 Hz, 1H), 9.93 (t, *J* = 7.0 Hz, 2H), 6.63 (d, *J* = 3.0 Hz, 1H), 6.61 (d, *J* = 8.2 Hz, 1H), 6.47 (dd, *J* = 3.0, 8.5 Hz, 1H), 4.36 (d, *J* = 6.3 Hz, 2H).

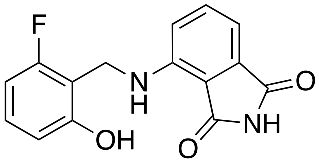

EB-5-71

4-((6-Fluoro-2-hydroxybenzyl)amino)isoindoline-1,3-dione (**EB-5-71**). Purified by silica gel chromatography using increasing concentrations of ethyl acetate (5-25%) in hexanes to give a yellow solid, 63% yield; ^{1}H NMR (400 MHz, $CDCl_3$): δ 10.96 (br s, 1H), 9.75 (br s, 1H), 7.55 (dd, J = 7.1, 8.5 Hz, 1H), 7.20 (d, J = 8.5 Hz, 1H), 7.14 (dt, J = 7.1, 8.3 Hz, 1H), 6.94 (d, J = 7.1 Hz, 1H), 6.75 (br t, J = 6.4 Hz, 1H), 6.70 (d, J = 8.3 Hz, 1H), 6.64 (ddd, J = 1.0, 8.3, 9.5 Hz, 1H), 4.46 (d, J = 6.4 Hz, 2H); HRMS $[M+H]^+$ calculated for $C_{15}H_{11}FN_2O_3$: 287.0832; found: 287.0847, err. = 5.2 ppm.

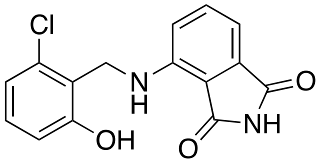

KM-5-29

4-((2-Chloro-6-hydroxybenzyl)amino)isoindoline-1,3-dione (**KM-5-29**). Previously described in: Soldano, A.; Yao, H.; Punchi Hewage, A. N. D.; Meraz, K.; Annor-Gyamfi, J. K.; Bunce, R. A.; Battaile, K. P.; Lovell, S.; Rivera, M. Small Molecule Inhibitors of the Bacterioferritin (BfrB)–Ferredoxin (Bfd) Complex Kill Biofilm-Embedded *Pseudomonas aeruginosa* Cells. *ACS Infect. Dis.* **2021**, *7*, 123-140; PMID: 33269912. https://doi.org/10.1021/acsinfecdis.0c00669

HRMS $[M+H]^+$ calculated for $C_{15}H_{11}ClN_2O_3$: 303.0536; found: 303.0538, err. = 0.7 ppm.

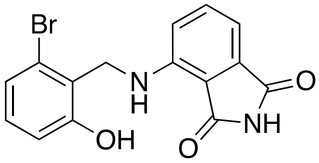

EB-5-72

4-((6-Bromo-2-hydroxybenzyl)amino)isoindoline-1,3-dione (**EB-5-72**). Purified by silica gel chromatography using increasing concentrations of ethyl acetate (5-25%) in hexanes to give a yellow solid, 60% yield; ^{1}H NMR (400 MHz, $CDCl_3$): δ 10.97 (br s, 1H), 10.45 (s, 1H), 7.56 (dd, J = 7.1, 8.5 Hz, 1H), 7.31 (d, J = 8.5 Hz, 1H), 7.14-7.06 (complex 2H), 6.96 (d, J = 7.1 Hz, 1H), 6.90 (m, 1H), 6.74 (br t, J = 6.1 Hz, 1H), 4.56 (d, J = 6.1 Hz, 2H); HRMS $[M+H]^+$ calculated for $C_{15}H_{11}BrN_2O_3$: 347.0031; found: 347.0033, err. = 0.6 ppm.

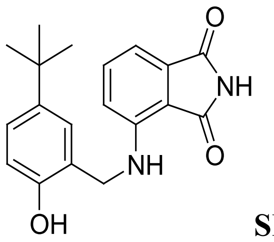

SB-16-53

4-(5-tert-butyl-2-hydroxy-benzylamino)-isoindole-1,3-dione (SB-16-53). The compound was synthesized following method B, using 5-tert-butyl-2-hydroxy-benzaldehyde (535 mg, 3.00 mmol), 4-amino-isoindole-1,3-dione (243 mg, 1.50 mmol), DMF (4.5 mL), $Ti(O^iPr)_4$ (0.93 g, 0.97 mL, 3.00 mmol), and $NaBH_4$ (117 mg, 3.13 mmol). The reaction was stirred at 20°C for 3 days. The reaction was poured in to a mixture of water (35 mL) and saturated aqueous $NaHCO_3$ (35 mL). The mixture was treated with 10% methanol in ethyl acetate (200 mL), then was shaken, and was filtered through Celite. The filtrate was separated, then the organic layer was washed with 1:1 water: brine (2 x 30 mL), and brine (20 mL), dried (Na_2SO_4), and concentrated. The crude material was purified by column on silica (1-15% (5% ammonium hydroxide in methanol)/dichloromethane, followed by preparative-HPLC, to give 4-(5-tert-butyl-2-hydroxy-benzylamino)-isoindole-1,3-dione (SB-16-53) as the trifluoroacetic acid salt (158 mg, 24%) as a yellow solid. ^1H NMR (400MHz, dmso) δ = 11.02 - 10.85 (m, 1H), 9.49 (s, 1H), 7.48 (dd, *J*=7.2, 8.4 Hz, 1H), 7.25 (d, *J*=2.3 Hz, 1H), 7.16 - 6.98 (m, 2H), 6.94 - 6.86 (m, 2H), 6.73 (d, *J*=8.6 Hz, 1H), 4.38 (d, *J*=5.9 Hz, 2H), 1.16 (s, 9H). HRMS $[M+H]^+$ calculated for $C_{19}H_{20}N_2O_3$: 325.1552; found:325.1550, err. = -0.6 ppm.

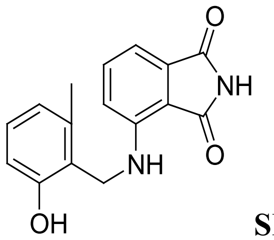

SB-15-171

4-(2-Hydroxy-6-methyl-benzylamino)-isoindole-1,3-dione (SB-15-171). The compound was synthesized following method B, using 2-hydroxy-6-methyl-benzaldehyde (287 mg, 2.11 mmol), 4-amino-isoindole-1,3-

dione (172 mg, 1.06 mmol), DMF (3.2 mL), $Ti(O^iPr)_4$ (654 mg, 682 mL, 2.11 mmol), and $NaBH_4$ (82 mg, 2.19 mmol). The reaction was stirred for 16 hours. The reaction was poured in to a mixture of water (16 mL) and saturated aqueous $NaHCO_3$ (16 mL). The mixture was treated with ethyl acetate (120 mL), then was shaken, and was filtered through Celite. The filtrate was separated, then the organic layer was washed with water (25 mL), and brine (20 mL), dried (Na_2SO_4), and concentrated. The crude material was purified by column on silica (0-7% methanol: dichloromethane), followed by preparative-TLC (5% (5% ammonium hydroxide: methanol): dichloromethane), to give 4-(2-hydroxy-6-methyl-benzylamino)-isoindole-1,3-dione (SB-15-171) (187 mg, 63%) as a yellow solid. ^{1}H NMR (300MHz, dmso) δ = 10.92 (s, 1H), 9.70 (s, 1H), 7.65 - 7.44 (m, 1H), 7.23 (d, *J*=8.2 Hz, 1H), 7.03 - 6.88 (m, 2H), 6.67 (dd, *J*=7.6, 19.9 Hz, 2H), 6.40 (t, *J*=5.6 Hz, 1H), 4.41 (d, *J*=5.3 Hz, 2H), 2.29 (s, 3H).

Compounds in Table S2.

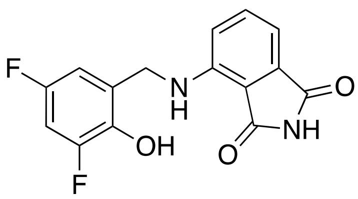

EB-5-14

4-((3,5-Difluoro-2-hydroxybenzyl)amino)isoindoline-1,3-dione (**EB-05-14**). Purified by silica gel chromatography using increasing concentrations of ethyl acetate (5-25%) in hexanes to give a yellow solid, 68% yield; ^{1}H NMR (400 MHz, DMSO-d_6): δ 10.98 (s, 1H), 9.82 (s, 1H), 7.49 (dd, *J* = 7.0, 8.4 Hz, 1H), 7.13-7.07 (complex, 2H), 6.95 (d, *J* = 7.0 Hz, 1H), 6.92 (d, *J* = 8.4 Hz, 1H), 6.92-6.88 (complex, 1H), 4.50 (d, *J* = 6.4 Hz, 2H); HRMS $[M+H]^+$ calculated for $C_{15}H_{10}F_2N_2O_3$: 305.0738; found: 305.0733, err. = -1.6 ppm.

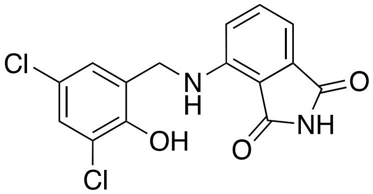

EB-5-15

4-((3,5-Dichloro-2-hydroxybenzyl)amino)isoindoline-1,3-dione (**EB-05-15**). Purified by silica gel chromatography using increasing concentrations of ethyl acetate (5-25%) in hexanes to give a yellow solid, 71% yield; ^{1}H NMR (400 MHz, DMSO-d_6): δ 10.99 (s, 1H), 9.92 (s, 1H), 7.50 (dd, J = 7.1, 8.5 Hz, 1H), 7.41 (d, J = 2.6 Hz, 1H), 7.23 (d, J = 2.6 Hz, 1H), 7.14 (t, J = 6.5 Hz, 1H), 6.95 (d, J = 7.1 Hz, 1H), 6.91 (d, J = 8.5 Hz, 1H), 4.51 (d, J = 6.5 Hz, 2H); HRMS $[M+H]^+$ calculated for $C_{15}H_{10}Cl_2N_2O_3$: 337.0147; found: 337.0148, err. = 0.3 ppm.

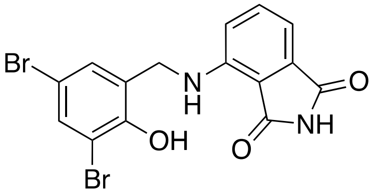

EB-5-13

4-((3,5-Dibromo-2-hydroxybenzyl)amino)isoindoline-1,3-dione (**EB-05-13**). Purified by silica gel chromatography using increasing concentrations of ethyl acetate (5-25%) in hexanes to give a yellow solid, 67% yield; ^{1}H NMR (400 MHz, DMSO-d_6): δ 10.99 (s, 1H), 9.80 (s, 1H), 7.63 (d, J = 2.4 Hz, 1H), 7.50 (dd, J = 7.0, 8.4 Hz , 1H), 7.38 (d, J = 2.4 Hz, 1H), 7.15 (t, J = 6.4 Hz, 1H), 6.96 (d, J = 7.0 Hz, 1H), 6.91 (d, J = 8.4 Hz, 1H), 4.51 (d, J = 6.5 Hz, 2H).

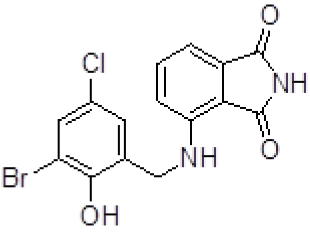

SB-16-165

4-(3-Bromo-5-chloro-2-hydroxy-benzylamino)-isoindole-1,3-dione (SB-16-165). The compound was synthesized following method B, using 3-bromo-5-chloro-2-hydroxy-benzaldehyde (589 mg, 2.50 mmol), 4-amino-isoindole-1,3-dione (203 mg, 1.25 mmol), DMF (3.75 mL), $Ti(O^{i}Pr)_4$ (0.78 g, 0.81 mL, 2.50 mmol), and $NaBH_4$ (98 mg, 2.61 mmol). The reaction was stirred for 16 hours. The reaction was poured in to a mixture of water (28 mL) and saturated aqueous $NaHCO_3$ (28 mL). The mixture was treated with 10% methanol in ethyl

acetate (200 mL), then was shaken, and was filtered through Celite. The filtrate was separated, then the organic layer was washed with 1:1 water: brine (50 mL), dried (Na_2SO_4), and concentrated. The crude material was purified by column on silica (1-10% (5% ammonium hydroxide: methanol): dichloromethane), followed by preparative-HPLC, to give 4-(3-bromo-5-chloro-2-hydroxy-benzylamino)-isoindole-1,3-dione (SB-16-165) as the trifluoroacetic acid salt (95 mg, 20%) as a yellow solid. ^{1}H NMR (300MHz, cdcl$_3$) δ = 9.79 - 9.60 (m, 1H), 7.36 - 7.00 (m, 2H), 6.93 - 6.57 (m, 4H), 3.81 (s, 4H), 3.44 - 3.21 (m, 1H), 2.86 - 2.56 (m, 2H), 1.40 - 1.27 (m, 4H).

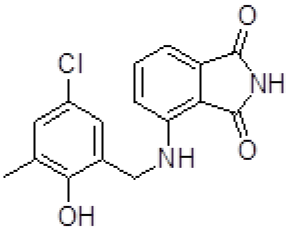

SB-16-156

4-(5-Chloro-2-hydroxy-3-methyl-benzylamino)-isoindole-1,3-dione (SB-16-156). The compound was synthesized following method B, using 5-chloro-2-hydroxy-3-methyl-benzaldehyde (427 mg, 2.50 mmol), 4-amino-isoindole-1,3-dione (203 mg, 1.25 mmol), DMF (3.75 mL), Ti(O^{i}Pr)$_4$ (0.78 g, 0.81 mL, 2.50 mmol), and $NaBH_4$ (98 mg, 2.61 mmol). The reaction was stirred for 3 days. The reaction was poured in to a mixture of water (28 mL) and saturated aqueous $NaHCO_3$ (28 mL). The mixture was treated with 10% methanol in ethyl acetate (200 mL), then was shaken, and was filtered through Celite. The filtrate was separated, then the organic layer was washed with water (2 x 25 mL), and brine (20 mL), dried (Na_2SO_4), and concentrated. The crude material was purified by column on silica (1-10% (5% ammonium hydroxide: methanol): dichloromethane), followed by preparative-HPLC, to give 4-(5-chloro-2-hydroxy-3-methyl-benzylamino)-isoindole-1,3-dione (SB-16-156) as the trifluoroacetic acid salt (207 mg, 39%) as a yellow solid. ^{1}H NMR (400MHz, dmso) δ = 7.57 - 7.36 (m, 1H), 7.15 - 7.02 (m, 3H), 6.91 (dd, *J*=2.0, 7.8 Hz, 2H), 4.43 (d, *J*=6.2 Hz, 2H), 2.16 (s, 3H). HRMS $[M+H]^+$ calculated for $C_{16}H_{13}ClN_2O_3$: 317.0693; found: 317.0696, err. = 0.9 ppm.

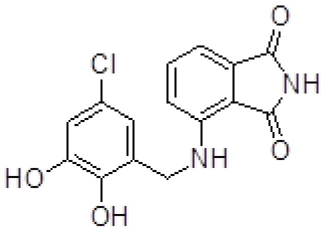

SB-16-168

4-(5-Chloro-2,3-dihydroxy-benzylamino)-isoindole-1,3-dione (SB-16-168).

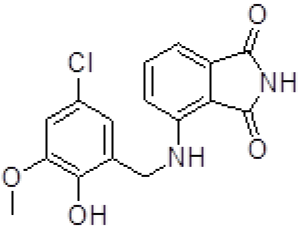

SB-16-153

4-(5-Chloro-2-hydroxy-3-methoxy-benzylamino)-isoindole-1,3-dione **(SB-16-153).** The compound was synthesized following method B, using 5-chloro-2-hydroxy-3-methoxy-benzaldehyde (467 mg, 2.50 mmol), 4-amino-isoindole-1,3-dione (203 mg, 1.25 mmol), DMF (3.75 mL), $Ti(O^iPr)_4$ (0.78 g, 0.81 mL, 2.50 mmol), and $NaBH_4$ (98 mg, 2.61 mmol). The reaction was stirred for 3 days. The reaction was poured in to a mixture of water (28 mL) and saturated aqueous $NaHCO_3$ (28 mL). The mixture was treated with 10% methanol in ethyl acetate (200 mL), then was shaken, and was filtered through Celite. The filtrate was separated, then the organic layer was washed with 1:1 water: brine (40 mL), dried (Na_2SO_4), and concentrated. The crude material was purified by column on silica (1-10% (5% ammonium hydroxide: methanol): dichloromethane), followed by preparative-HPLC, to give 4-(5-chloro-2-hydroxy-3-methoxy-benzylamino)-isoindole-1,3-dione (SB-16-153) as the trifluoroacetic acid salt (125 mg, 22%) as a yellow solid. ^{1}H NMR (400MHz, dmso) δ = 10.94 (s, 1H), 9.21 (s, 1H), 7.46 (dd, *J*=7.2, 8.4 Hz, 1H), 7.02 (t, *J*=6.2 Hz, 1H), 6.93 - 6.89 (m, 3H), 6.84 (d, *J*=2.3 Hz, 1H), 4.41 (d, *J*=6.2 Hz, 2H), 3.79 (s, 3H).

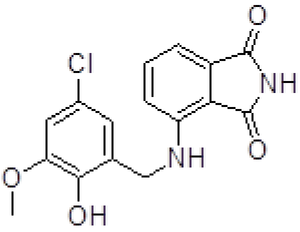

SB-16-167

Synthesis of 4-(5-chloro-2-hydroxy-3-methoxy-benzylamino)-isoindole-1,3-dione (SB-16-167). A mixture of 4-(5-chloro-2-hydroxy-3-methoxy-benzylamino)-isoindole-1,3-dione trifluoroacetic acid salt (96 mg, 0.22 mmol) in 10% methanol in ethyl acetate (40 mL) was washed with saturated aqueous $NaHCO_3$ (2 x 20 mL), and brine (15 mL), dried (Na_2SO_4), and concentrated, to give 4-(5-chloro-2-hydroxy-3-methoxy-benzylamino)-isoindole-1,3-dione (92 mg, >100%) as a yellow solid, that was used without further purification.

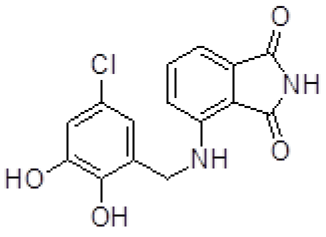

SB-16-168

Synthesis of 4-(5-chloro-2,3-dihydroxy-benzylamino)-isoindole-1,3-dione (SB-16-168).

To a -78°C mixture of 4-(5-chloro-2-hydroxy-3-methoxy-benzylamino)-isoindole-1,3-dione (92 mg, 0.28 mmol) in dichloromethane (1.4 mL) was added a 1M solution of boron tribromide in dichloromethane (0.84 mL, 0.84 mmol). The reaction was stirred at 78°C for 30 minutes, and at 0°C for 16 hours. The reaction was treated with methanol (1.3 mL), saturated aqueous $NaHCO_3$ (30 mL), and 10% methanol in ethyl acetate (40 mL). The mixture was shaken, and the layers were separated. The organic layer was washed with brine (15 mL), dried (Na_2SO_4), and concentrated. The crude material was purified by preparative-HPLC, to give 4-(5-chloro-2,3-dihydroxy-benzylamino)-isoindole-1,3-dione (SB-16-168) as the trifluoroacetic acid salt (42 mg, 35%) as a yellow solid. ^1H NMR (400MHz, dmso) δ = 10.93 (s, 1H), 9.87 (s, 1H), 8.91 (s, 1H), 7.47 (dd, *J*=7.2, 8.4 Hz, 1H), 6.99 (t, *J*=6.4 Hz, 1H), 6.91 (dd, *J*=4.7, 7.8 Hz, 2H), 6.75 - 6.59 (m, 2H), 4.39 (d, *J*=6.2 Hz, 2H). HRMS $[M+H]^+$ calculated for $C_{15}H_{11}ClN_2O_4$: 319.0486; found: 319.0491, err. = 1.6 ppm.

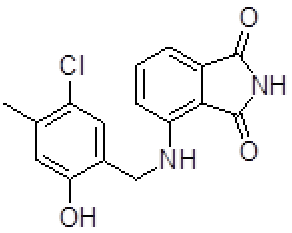

SB-16-159

4-(5-Chloro-2-hydroxy-4-methyl-benzylamino)-isoindole-1,3-dione (SB-16-159). The compound was synthesized following method B, using 5-chloro-2-hydroxy-4-methyl-benzaldehyde (427 mg, 2.50 mmol), 4-amino-isoindole-1,3-dione (203 mg, 1.25 mmol), DMF (3.75 mL), $Ti(O^iPr)_4$ (0.78 g, 0.81 mL, 2.50 mmol), and $NaBH_4$ (98 mg, 2.61 mmol). The reaction was stirred for 3 days. The reaction was poured in to a mixture of water (28 mL) and saturated aqueous $NaHCO_3$ (28 mL). The mixture was treated with 10% methanol in ethyl acetate (200 mL), then was shaken, and was filtered through Celite. The filtrate was separated, then the organic layer was washed with 1:1 water: brine (40 mL), dried (Na_2SO_4), and concentrated. The crude material was purified by column on silica (1-10% (5% ammonium hydroxide: methanol): dichloromethane), followed by preparative-HPLC, to give 4-(5-chloro-2-hydroxy-4-methyl-benzylamino)-isoindole-1,3-dione (SB-16-159) as the trifluoroacetic acid salt (162 mg, 30%) as a yellow solid. ^{1}H NMR (400MHz, dmso) δ = 10.94 (s, 1H), 9.91 (s, 1H), 7.52 - 7.42 (m, 1H), 7.27 - 7.12 (m, 1H), 7.00 (t, *J*=6.4 Hz, 1H), 6.92 (dd, *J*=7.8, 16.4 Hz, 2H), 6.76 (s, 1H), 4.36 (d, *J*=6.6 Hz, 2H), 2.18 (s, 3H).

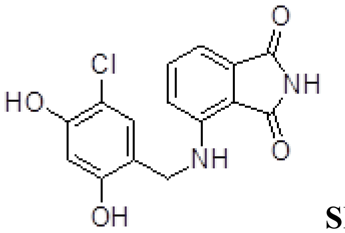

SB-16-160

4-(5-Chloro-2,4-dihydroxy-benzylamino)-isoindole-1,3-dione (SB-16-160). The compound was synthesized following method B, using 5-chloro-2,4-dihydroxy-benzaldehyde (431 mg, 2.50 mmol), 4-amino-isoindole-1,3-dione (203 mg, 1.25 mmol), DMF (3.75 mL), $Ti(O^iPr)_4$ (1.09 g, 1.13 mL, 3.50 mmol), and $NaBH_4$ (137 mg, 3.65 mmol). The reaction was stirred for 16 hours. The reaction was poured in to a mixture of water (28 mL) and saturated aqueous $NaHCO_3$ (28 mL). The mixture was treated with 10% methanol in ethyl acetate (200 mL), then was shaken, and was filtered through Celite. The filtrate was separated, then the organic layer was washed with water (2 x 25 mL), and brine (20 mL), dried (Na_2SO_4), and concentrated. The crude material was purified by column on silica (1-15% (5% ammonium hydroxide: methanol): dichloromethane). The product was treated with

5% methanol in dichloromethane (50 mL), then was filtered, and the filtrate was concentrated. The impure product was purified by preparative-HPLC, to give 4-(5-chloro-2,4-dihydroxy-benzylamino)-isoindole-1,3-dione (SB-16-160) as the trifluoroacetic acid salt (23 mg, 4%) as a yellow solid. ^{1}H NMR (400MHz, cd$_3$od) δ = 7.53 - 7.43 (m, 1H), 7.10 (s, 1H), 7.02 - 6.92 (m, 2H), 6.45 (s, 1H), 4.36 (s, 2H). HRMS $[M+H]^+$ calculated for $C_{15}H_{11}ClN_2O_4$: 319.0486; found: 319.0486, err. = 0.0 ppm.

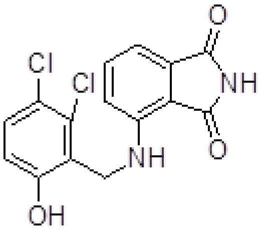

SB-16-152

4-(2,3-Dichloro-6-hydroxy-benzylamino)-isoindole-1,3-dione (SB-16-152). The compound was synthesized following method B, using 2,3-dichloro-6-hydroxy-benzaldehyde (573 mg, 3.00 mmol), 4-amino-isoindole-1,3-dione (243 mg, 1.50 mmol), DMF (4.5 mL), Ti(O^{i}Pr)$_4$ (0.93 g, 0.97 mL, 3.00 mmol). and $NaBH_4$ (117 mg, 3.13 mmol). The reaction was stirred for 2 days. The reaction was poured in to a mixture of water (35 mL) and saturated aqueous $NaHCO_3$ (35 mL). The mixture was treated with 10% methanol in ethyl acetate (250 mL), then was shaken, and was filtered through Celite. The filtrate was separated, then the organic layer was washed with water (30 mL), 1:1 water: brine (30 mL), dried (Na_2SO_4), and concentrated. The crude material was purified by column on silica (1-10% (5% ammonium hydroxide: methanol): dichloromethane), followed by preparative-HPLC, to give 4-(2,3-dichloro-6-hydroxy-benzylamino)-isoindole-1,3-dione (SB-16-152) as the trifluoroacetic acid salt (144 mg, 21%) as a yellow solid. ^{1}H NMR (400MHz, dmso) δ = 10.96 (s, 1H), 10.71 (s, 1H), 7.54 (dd, *J*=7.2, 8.4 Hz, 1H), 7.38 (d, *J*=8.6 Hz, 1H), 7.26 (d, *J*=8.6 Hz, 1H), 6.93 (d, *J*=7.0 Hz, 1H), 6.87 (d, *J*=8.6 Hz, 1H), 6.77 (t, *J*=6.2 Hz, 1H), 4.66 - 4.47 (m, 2H).

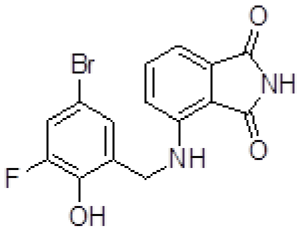

SB-16-118

4-(5-Bromo-3-fluoro-2-hydroxy-benzylamino)-isoindole-1,3-dione (SB-16-118). The compound was synthesized following method B, using 5-bromo-3-fluoro-2-hydroxy-benzaldehyde (657 mg, 3.00 mmol), 4-amino-isoindole-1,3-dione (243 mg, 1.50 mmol), DMF (4.5 mL), $Ti(O^iPr)_4$ (0.93 g, 0.97 mL, 3.00 mmol), and $NaBH_4$ (117 mg, 3.13 mmol). The reaction was stirred for 3 days. The reaction was poured in to a mixture of water (35 mL) and saturated aqueous $NaHCO_3$ (35 mL). The mixture was treated with 10% methanol in ethyl acetate (250 mL), then was shaken, and was filtered through Celite. The filtrate was separated, then the organic layer was treated with water (20 mL) and brine (20 mL). The mixture was shaken, then was filtered through Celite. The filtrate was separated, then the organic layer was dried (Na_2SO_4), and concentrated. The crude material was purified by column on silica (1-10% (5% ammonium hydroxide: methanol): dichloromethane), followed by preparative-HPLC, to give 4-(5-bromo-3-fluoro-2-hydroxy-benzylamino)-isoindole-1,3-dione (SB-16-118) as the trifluoroacetic acid salt (175 mg, 24%) as a yellow solid. ^1H NMR (400MHz, dmso) δ = 10.45 (br. s., 1H), 9.81 - 9.56 (m, 1H), 7.03 - 6.89 (m, 1H), 6.83 (br. s., 1H), 6.70 (br. s., 1H), 6.57 (br. s., 1H), 6.46 - 6.33 (m, 2H), 3.95 (br. s., 2H). HRMS $[M+H]^+$ calculated for $C_{15}H_{10}BrFN_2O_3$: 364.9937; found: 364.9937, err. = 1.1 ppm.

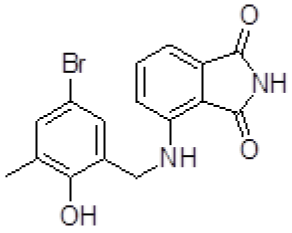

SB-16-106

4-(5-Bromo-2-hydroxy-3-methyl-benzylamino)-isoindole-1,3-dione (SB-16-106). The compound was synthesized following method B, using 5-bromo-2-hydroxy-3-methyl-benzaldehyde (645 mg, 3.00 mmol), 4-

amino-isoindole-1,3-dione (243 mg, 1.50 mmol), DMF (4.5 mL), $Ti(O^iPr)_4$ (0.93 g, 0.97 mL, 3.00 mmol), and $NaBH_4$ (117 mg, 3.13 mmol). The reaction was stirred for 2 days. The reaction was poured in to a mixture of water (35 mL) and saturated aqueous $NaHCO_3$ (35 mL). The mixture was treated with 10% methanol in ethyl acetate (250 mL), then was shaken, and was filtered through Celite. The filtrate was separated, then the organic layer was washed with water (30 mL), 1:1 water: brine (2 x 40 mL), and brine (25 mL), dried (Na_2SO_4), and concentrated. The crude material was purified by column on silica (1-10% (5% ammonium hydroxide: methanol): dichloromethane), followed by preparative-HPLC, to give 4-(5-bromo-2-hydroxy-3-methyl-benzylamino)-isoindole-1,3-dione (SB-16-106) as the trifluoroacetic acid salt (107 mg, 15%) as a yellow solid. ^{1}H NMR (400MHz, dmso) δ = 10.94 (s, 1H), 9.07 - 8.79 (m, 1H), 7.47 (dd, J=7.4, 8.2 Hz, 1H), 7.18 (dd, J=2.3, 10.2 Hz, 2H), 7.05 (t, J=6.2 Hz, 1H), 6.92 (dd, J=3.9, 7.8 Hz, 2H), 4.44 (d, J=6.2 Hz, 2H), 2.23 - 2.08 (m, 3H). HRMS $[M+H]^+$ calculated for $C_{16}H_{13}BrN_2O_3$: 361.0188; found: 361.0199, err. = 3.0 ppm.

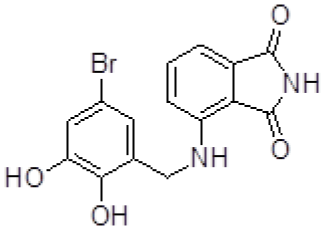

SB-16-136

4-(5-Bromo-2,3-dihydroxy-benzylamino)-isoindole-1,3-dione (SB-16-136).

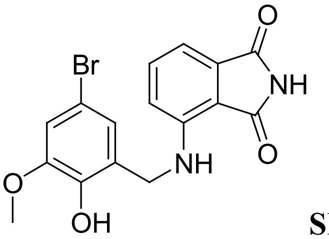

SB-16-131

4-(5-Bromo-2-hydroxy-3-methoxy-benzylamino)-isoindole-1,3-dione (SB-16-131). The compound was synthesized following method B, using 5-bromo-2-hydroxy-3-methoxy-benzaldehyde (802 mg, 3.00 mmol), 4-amino-isoindole-1,3-dione (243 mg, 1.50 mmol), DMF (4.5 mL), $Ti(O^iPr)_4$ (0.93 g, 0.97 mL, 3.00 mmol), and $NaBH_4$ (117 mg, 3.13 mmol). The reaction was stirred for 2 days. The reaction was poured in to a mixture of

water (35 mL) and saturated aqueous $NaHCO_3$ (35 mL). The mixture was treated with 10% methanol in ethyl acetate (250 mL), then was shaken, and was filtered through Celite. The filtrate was separated, then the organic layer was washed with water (2 x 25 mL), and brine (25 mL), dried (Na_2SO_4), and concentrated. The crude material was purified by column on silica (1-10% (5% ammonium hydroxide: methanol): dichloromethane). The impure product was treated with DMF (5 ml) followed by dropwise addition of 0.1% trifluoroacetic acid in water (2.5 mL). The mixture was filtered, then the filtrate was purified by preparative-HPLC. The pure product was treated with 10% methanol in ethyl acetate (50 mL), then the mixture was washed with saturated aqueous $NaHCO_3$ (2 x 20 mL), water (2 x 15 mL), and brine (15 mL), dried (Na_2SO_4), and concentrated to give 4-(5-bromo-2-hydroxy-3-methoxy-benzylamino)-isoindole-1,3-dione (SB-16-131) (305 mg, 40%) as a yellow solid. ^{1}H NMR (400MHz, dmso) δ = 10.44 (br. s., 1H), 8.74 (s, 1H), 7.04 - 6.80 (m, 1H), 6.61 - 6.18 (m, 5H), 3.90 (d, *J*=5.5 Hz, 2H), 2.05 - 1.88 (m, 2H).

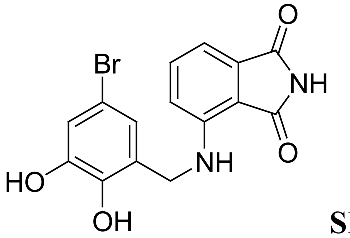

SB-16-136

4-(5-Bromo-2,3-dihydroxy-benzylamino)-isoindole-1,3-dione (SB-16-136). To a -78°C mixture of 4-(5-bromo-2-hydroxy-3-methoxy-benzylamino)-isoindole-1,3-dione (SB-16-131) (267 mg, 0.71 mmol) in dichloromethane (3.5 mL) was added a 1M solution of boron tribromide in dichloromethane (2.1 mL), 2.10 mmol). The reaction was stirred at -78°C for 30 minutes, and at 0°C for 16 hours. The reaction was treated with methanol (3 mL), saturated aqueous $NaHCO_3$ (40 mL), and 10% methanol in ethyl acetate (60 mL). The mixture was shaken, then was filtered through Celite. The filtrate was separated, then the organic layer was washed with brine (15 mL), dried (Na_2SO_4), and concentrated. The crude material was purified by preparative-HPLC, to give 4-(5-bromo-2,3-dihydroxy-benzylamino)-isoindole-1,3-dione (SB-16-136) as the trifluoroacetic acid salt (139 mg, 41%) as a yellow solid. ^{1}H NMR (400MHz, dmso) δ = 11.04 - 10.79 (m, 1H), 9.87 (s, 1H), 8.94 (s, 1H), 7.47

(dd, *J*=7.2, 8.4 Hz, 1H), 7.02 - 6.96 (m, 1H), 6.94 - 6.88 (m, 2H), 6.81 (q, *J*=2.3 Hz, 2H), 4.38 (d, *J*=6.2 Hz, 2H). HRMS $[M+H]^+$ calculated for $C_{15}H_{11}BrN_2O_4$: 362.9980; found: 362.9980, err. = 2.8 ppm.

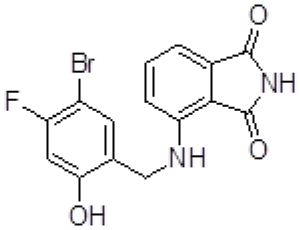

SB-16-112

4-(5-Bromo-4-fluoro-2-hydroxy-benzylamino)-isoindole-1,3-dione (SB-16-112). The compound was synthesized following method B, using 5-bromo-4-fluoro-2-hydroxy-benzaldehyde (657 mg, 3.00 mmol), 4-amino-isoindole-1,3-dione (243 mg, 1.50 mmol), DMF (4.5 mL), $Ti(O^iPr)_4$ (0.93 g, 0.97 mL, 3.00 mmol), and $NaBH_4$ (117 mg, 3.13 mmol). The reaction was stirred for 2 days. The reaction was poured in to a mixture of water (35 mL) and saturated aqueous $NaHCO_3$ (35 mL). The mixture was treated with 10% methanol in ethyl acetate (250 mL), then was shaken, and was filtered through Celite. The filtrate was separated, then the organic layer was washed with 1:1 water: brine (2 x 30 mL), and brine (20 mL), dried (Na_2SO_4), and concentrated. The crude material was purified by column on silica (1-10% (5% ammonium hydroxide: methanol): dichloromethane). The impure product was treated with DMF (5 ml) followed by dropwise addition of 0.1% trifluoroacetic acid in water (2.5 mL). The mixture was filtered, then the filtrate was purified by preparative-HPLC, to give 4-(5-bromo-4-fluoro-2-hydroxy-benzylamino)-isoindole-1,3-dione (SB-16-112) as the trifluoroacetic acid salt (105 mg, 15%) as a yellow solid. ^1H NMR (400MHz, dmso) δ = 10.95 (br. s., 1H), 10.58 (s, 1H), 7.59 - 7.31 (m, 2H), 7.10 - 6.87 (m, 3H), 6.76 (d, *J*=10.5 Hz, 1H), 4.38 (d, *J*=5.9 Hz, 2H). HRMS $[M+H]^+$ calculated for $C_{15}H_{10}BrFN_2O_3$: 364.9937; found: 364.9945, err. = 2.2 ppm.

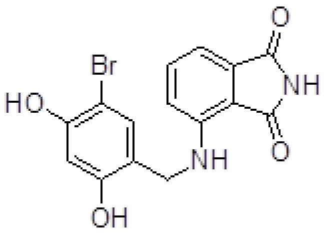

SB-16-137

4-(5-Bromo-2,4-dihydroxy-benzylamino)-isoindole-1,3-dione (SB-16-137). The compound was synthesized following method B, using 5-bromo-2,4-dihydroxy-benzaldehyde (651 mg, 3.00 mmol), 4-amino-isoindole-1,3-dione (243 mg, 1.50 mmol), DMF (4.5 mL), $Ti(O^iPr)_4$ (1.24 g, 1.29 mL, 4.00 mmol) and $NaBH_4$ (156 mg, 4.17 mmol). The reaction was stirred for 2 days. The reaction was poured in to a mixture of water (35 mL) and saturated aqueous $NaHCO_3$ (35 mL). The mixture was treated with 10% methanol in ethyl acetate (250 mL), then was shaken, and was filtered through Celite. The filtrate was separated, then the organic layer was washed with water (2 x 25 mL), and brine (20 mL), dried (Na_2SO_4), and concentrated. The crude material was purified by column on silica (1-10% (5% ammonium hydroxide: methanol): dichloromethane) followed by preparative-HPLC, to give 4-(5-bromo-2,4-dihydroxy-benzylamino)-isoindole-1,3-dione (SB-16-137) as the trifluoroacetic acid salt (65 mg, 9%) as an orange solid. ^{1}H NMR (400MHz, dmso) δ = 10.91 (s, 1H), 9.97 (s, 1H), 9.85 (s, 1H), 7.55 - 7.44 (m, 1H), 7.26 (s, 1H), 7.01 (d, *J*=8.6 Hz, 1H), 6.95 - 6.86 (m, 2H), 6.52 (s, 1H), 4.28 (d, *J*=5.9 Hz, 2H). HRMS $[M+H]^+$ calculated for $C_{15}H_{11}BrN_2O_4$: 362.9980; found: 362.9940, err. = 1.9 ppm.

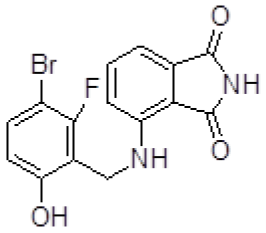

SB-16-145

4-(3-Bromo-2-fluoro-6-hydroxy-benzylamino)-isoindole-1,3-dione (SB-16-145).

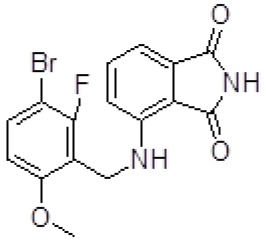

SB-16-138

4-(3-Bromo-2-fluoro-6-methoxy-benzylamino)-isoindole-1,3-dione (SB-16-138). The compound was synthesized following method B, using -bromo-2-fluoro-6-methoxy-benzaldehyde (1.00 g, 4.29 mmol), 4-amino-isoindole-1,3-dione (349 mg, 2.15 mmol), DMF (6.5 mL), $Ti(O^iPr)_4$ (1.33 g, 1.38 mL, 4.29 mmol), and $NaBH_4$

(167 mg, 4.47 mmol). The reaction was stirred for 16 hours. The reaction was poured in to a mixture of water (50 mL) and saturated aqueous $NaHCO_3$ (50 mL). The mixture was treated with 10% methanol in ethyl acetate (250 mL), then was shaken, and the layers were separated. The organic layer was washed with water (2 x 25 mL), and brine (25 mL), dried (Na_2SO_4), and concentrated. The crude material was purified by column on silica (1-10% (5% ammonium hydroxide: methanol): dichloromethane) followed by preparative-HPLC. The product was treated with 10% methanol in ethyl acetate (70 mL), then the mixture was washed with saturated aqueous $NaHCO_3$ (2 x 20 mL) and brine (15 mL), dried (Na_2SO_4), and concentrated, to give 4-(3-bromo-2-fluoro-6-methoxy-benzylamino)-isoindole-1,3-dione (SB-16-138) (82 mg, 10%) as a yellow solid. ^1H NMR (400MHz, cd_3od) δ = 7.51 (td, *J*=7.6, 15.2 Hz, 1H), 7.17 (d, *J*=9.0 Hz, 1H), 6.99 (d, *J*=7.4 Hz, 1H), 6.82 (d, *J*=9.0 Hz, 1H), 4.58 (br. s., 1H), 3.93 (s, 1H).

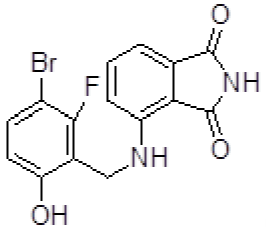

SB-16-145

4-(3-Bromo-2-fluoro-6-hydroxy-benzylamino)-isoindole-1,3-dione (SB-16-145). To a -78°C mixture of 4-(3-bromo-2-fluoro-6-methoxy-benzylamino)-isoindole-1,3-dione (82 mg, 0.22 mmol) in dichloromethane (1.1 mL) was added a 1M solution of boron tribromide in dichloromethane (0.66 mL, 0.66 mmol). The reaction was stirred at -78°C for 30 minutes, and at 0°C for 20 hours. The reaction was treated with methanol (1 mL), saturated aqueous $NaHCO_3$ (25 mL), and 10% methanol in ethyl acetate (40 mL). The mixture was shaken, then the organic layer was washed with brine (15 mL), dried (Na_2SO_4), and concentrated. The crude material was purified by preparative-HPLC, to give 4-(3-bromo-2-fluoro-6-hydroxy-benzylamino)-isoindole-1,3-dione (SB-16-145) as the trifluoroacetic acid salt (43 mg, 41%) as a yellow solid. ^1H NMR (400MHz, dmso) δ = 10.96 (s, 1H), 10.68 (d, *J*=1.6 Hz, 1H), 7.54 (dd, *J*=7.2, 8.4 Hz, 1H), 7.39 (t, *J*=8.6 Hz, 1H), 7.16 (d, *J*=8.6 Hz, 1H), 6.93 (d, *J*=7.0 Hz,

1H), 6.78 (t, *J*=6.4 Hz, 1H), 6.67 (dd, *J*=1.2, 9.0 Hz, 1H), 4.49 (d, *J*=6.2 Hz, 2H). HRMS $[M+H]^+$ calculated for $C_{15}H_{10}BrFN_2O_3$: 364.9937; found: 364.9958, err. = 5.8 ppm.

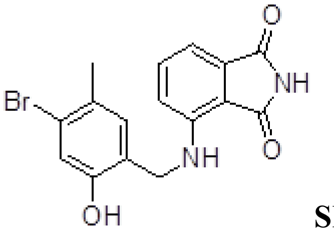

SB-16-183

4-(4-Bromo-2-hydroxy-5-methyl-benzylamino)-isoindole-1,3-dione (SB-16-183). The compound was synthesized following method B, using 4-bromo-2-hydroxy-5-methyl-benzaldehyde (538 mg, 2.50 mmol), 4-amino-isoindole-1,3-dione (203 mg, 1.25 mmol), DMF (3.75 mL), $Ti(O^iPr)_4$ (0.78 g, 0.81 mL, 2.50 mmol) and $NaBH_4$ (98 mg, 2.61 mmol). The reaction was stirred for 3 days. The reaction was poured in to a mixture of water (28 mL) and saturated aqueous $NaHCO_3$ (28 mL). The mixture was treated with 10% methanol in ethyl acetate (200 mL), then was shaken, and was filtered through Celite. The filtrate was separated, then the organic layer was washed with water (2 x 25 mL), and brine (20 mL), dried (Na_2SO_4), and concentrated. The crude material was purified by column on silica (1-10% (5% ammonium hydroxide: methanol): dichloromethane), followed by preparative-HPLC, to give 4-(4-bromo-2-hydroxy-5-methyl-benzylamino)-isoindole-1,3-dione (SB-16-183) as the trifluoroacetic acid salt (152 mg, 26%) as a yellow solid. ^1H NMR (400MHz, dmso) δ = 10.93 (s, 1H), 10.11 - 9.84 (m, 1H), 7.54 - 7.37 (m, 1H), 7.23 - 7.11 (m, 1H), 7.00 (s, 1H), 6.97 - 6.88 (m, 3H), 4.34 (d, *J*=6.2 Hz, 2H), 2.15 (s, 3H). HRMS $[M+H]^+$ calculated for $C_{16}H_{13}BrN_2O_3$: 361.0188; found: 361.0194, err. = 1.7 ppm.

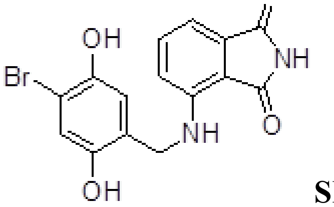

SB-16-186

4-(4-Bromo-2,5-dihydroxy-benzylamino)-isoindole-1,3-dione (SB-16-186).

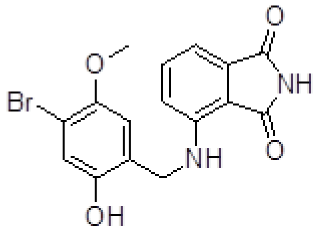

SB-16-173

4-(4-Bromo-2-hydroxy-5-methoxy-benzylamino)-isoindole-1,3-dione (SB-16-173). The compound was synthesized following method B, using 4-bromo-2-hydroxy-5-methoxy-benzaldehyde (578 mg, 2.50 mmol), 4-amino-isoindole-1,3-dione (203 mg, 1.25 mmol), DMF (3.75 mL), $Ti(O^iPr)_4$ (0.78 g, 0.81 mL, 2.50 mmol), and $NaBH_4$ (98 mg, 2.61 mmol). The reaction was stirred for 16 hours. The reaction was poured in to a mixture of water (28 mL) and saturated aqueous $NaHCO_3$ (28 mL). The mixture was treated with 10% methanol in ethyl acetate (200 mL), then was shaken, and was filtered through Celite. The filtrate was separated, then the organic layer was washed with water (2 x 25 mL), and brine (20 mL), dried (Na_2SO_4), and concentrated. The crude material was purified by column on silica (1-10% (5% ammonium hydroxide: methanol): dichloromethane), followed by preparative-HPLC. The product was treated with 10% methanol in ethyl acetate (50 mL), then the mixture was washed with saturated aqueous $NaHCO_3$ (2 x 20 mL), and brine (15 mL), dried (Na_2SO_4), and concentrated, to give 4-(4-bromo-2-hydroxy-5-methoxy-benzylamino)-isoindole-1,3-dione (SB-16-173) (149 mg, 32%) as a yellow solid. ^1H NMR (400MHz, dmso) δ = 10.93 (s, 1H), 9.70 (s, 1H), 7.48 (dd, *J*=7.2, 8.4 Hz, 1H), 7.19 - 6.81 (m, 5H), 4.37 (d, *J*=6.2 Hz, 2H), 3.66 (s, 3H).

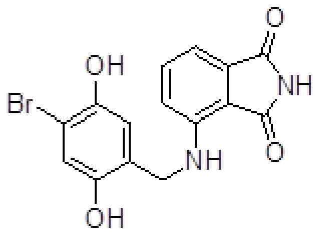

SB-16-186

4-(4-Bromo-2,5-dihydroxy-benzylamino)-isoindole-1,3-dione (SB-16-186). To a -78°C mixture of 4-(4-bromo-2-hydroxy-5-methoxy-benzylamino)-isoindole-1,3-dione (132 mg, 0.35 mmol) in dichloromethane (1.8 mL) was added a 1M solution of boron tribromide in dichloromethane (1.05 mL, 1.05 mmol). The reaction was stirred at -78°C for 30 minutes, and at 0°C for 20 hours. The reaction was treated with methanol (1.6 mL), saturated

aqueous $NaHCO_3$ (35 mL), and 10% methanol in ethyl acetate (45 mL). The mixture was shaken, then the organic layer was washed with brine (15 mL), dried (Na_2SO_4), and concentrated. The crude material was purified by preparative-HPLC, to give 4-(4-bromo-2,5-dihydroxy-benzylamino)-isoindole-1,3-dione (SB-16-186) as the trifluoroacetic acid salt (12 mg, 7%) as a red solid. ^{1}H NMR (400MHz, dmso) δ = 10.98 (s, 1H), 7.58 (s, 1H), 7.52 - 7.39 (m, 1H), 7.00 - 6.83 (m, 3H), 6.60 (s, 1H), 4.31 (d, *J*=4.3 Hz, 2H).

Compounds in Table S3.

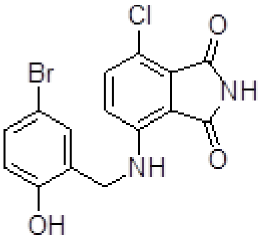

SB-16-124

4-(5-Bromo-2-hydroxy-benzylamino)-7-chloro-isoindole-1,3-dione (SB-16-124).

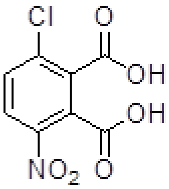

SB-16-50

3-Chloro-6-nitro-phthalic acid (SB-16-50). To a 0ºC mixture of nitric acid (2.8 mL) and sulfuric acid (7 mL) was added 4-chloro-isobenzofuran-1,3-dione (4.00 g, 21.91 mmol). The reaction was allowed to warm to 20ºC, and was stirred for 20 hours. The reaction was cooled to 0ºC and ice (~30 g) was added. The mixture was allowed to warm to 20ºC, then was filtered, and the solid was washed with water (10 mL). The solid was dried under high vacuum, to give 3-chloro-6-nitro-phthalic acid (SB-16-50) (1.40 g, 26%) as a white solid. ^{1}H NMR (400MHz, dmso) δ = 8.13 (d, *J*=9.0 Hz, 1H), 7.98 - 7.77 (m, 1H).

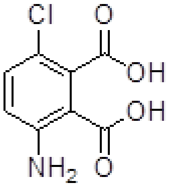

SB-16-54

3-Amino-6-chloro-phthalic acid (SB-16-54). A mixture of 3-chloro-6-nitro-phthalic acid (1.40 g, 5.70 mmol) in ethanol (180 mL) was treated with a slurry of Raney nickel in water (4.5 g). The reaction was placed under an atmosphere of hydrogen, and was stirred for 3 days. The reaction was filtered through Celite, and the filtrate was concentrated, to give 3-amino-6-chloro-phthalic acid (SB-16-54) (1.05 g, 85%) as a dark solid. ^{1}H NMR (400MHz, dmso) δ = 8.09 - 7.81 (m, 1H), 7.49 (br. s., 1H), 7.24 (br. s., 1H), 6.99 - 6.34 (m, 2H).

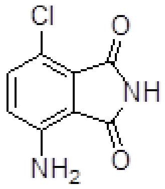

SB-16-75

4-Amino-7-chloro-isoindole-1,3-dione (SB-16-75). To a mixture of 3-amino-6-chloro-phthalic acid (1.05 g, 4.87 mmol) in NMP (16 mL) was added urea (0.59 g, 9.74 mmol). The reaction was heated at 140ºC for 2 hours, then at 160ºC for 4 hours. The reaction was poured in to 10% methanol in ethyl acetate (200 mL), then the mixture was treated with water (50 mL). The mixture was shaken, and was filtered through Celite. The filtrate was separated, then the organic layer was washed with water (2 x 30 mL) and brine (25 mL), dried (Na_2SO_4), and concentrated. The crude material was purified by column on silica (1-10% (5% ammonium hydroxide: methanol): dichloromethane). The product was treated with ethyl acetate (50 mL), then the mixture was washed with water (3 x 20 mL), and brine (20 mL), dried (Na_2SO_4), and concentrated, to give 4-amino-7-chloro-isoindole-1,3-dione (SB-16-75) (90% purity by ^{1}H NMR) (280 mg, 29%) as a yellow solid. ^{1}H NMR (400MHz, dmso) δ = 11.05 (br. s., 1H), 7.36 (d, *J*=9.0 Hz, 1H), 6.94 (d, *J*=9.0 Hz, 1H), 6.51 (br. s., 2H).

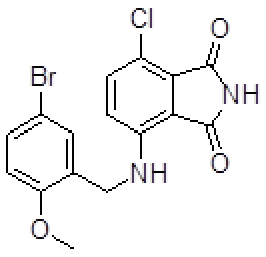

4-(5-Bromo-2-methoxy-benzylamino)-7-chloro-isoindole-1,3-dione (SB-16-84). A mixture of 4-amino-7-chloro-isoindole-1,3-dione (280 mg, 1.42 mmol) and 4-bromo-2-bromomethyl-1-methoxy-benzene (398 mg, 1.42 mmol) in DMF (2.8 mL) was heated at 100°C for 20 hours. The reaction was poured in to a mixture of water (23 mL) and saturated aqueous $NaHCO_3$ (23 mL). The mixture was extracted with 10% methanol in ethyl acetate (2 x 50 mL). The combined organic extracts were washed with water (2 x 20 mL), and brine (15 mL), dried (Na_2SO_4), and concentrated. The crude material was purified by column on silica (1-10% (5% ammonium hydroxide: methanol): dichloromethane. The impure product was treated with dichloromethane (8 mL) and methanol (2 mL), then was triturated, and was filtered. The solid was washed with dichloromethane (5 mL), to give 4-(5-bromo-2-methoxy-benzylamino)-7-chloro-isoindole-1,3-dione (SB-16-84) (185 mg, 33%) as a yellow solid. ^{1}H NMR (400MHz, dmso) δ = 11.15 (s, 1H), 7.45 - 7.33 (m, 3H), 7.18 (t, *J*=6.4 Hz, 1H), 6.98 (d, *J*=8.6 Hz, 1H), 6.89 (d, *J*=9.0 Hz, 1H), 4.43 (d, *J*=6.2 Hz, 2H), 3.82 (s, 3H).

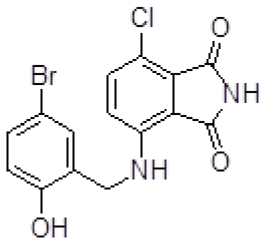

SB-16-124

4-(5-Bromo-2-hydroxy-benzylamino)-7-chloro-isoindole-1,3-dione (SB-16-124). To a -78 °C mixture of 4-(5-bromo-2-methoxy-benzylamino)-7-chloro-isoindole-1,3-dione (135 mg, 0.34 mmol) in dichloromethane (1.7 mL) was added a 1M solution of boron tribromide in dichloromethane (1.00 mL, 1.00 mmol). The reaction was stirred at -78°C for 30 minutes, and at 0°C for 16 hours. The reaction was treated with methanol (1.4 mL), saturated aqueous $NaHCO_3$ (20 mL), and 10% methanol in ethyl acetate (50 mL). The mixture was shaken, then the organic layer was washed with brine (20 mL), dried (Na_2SO_4), and concentrated. The crude material was purified by preparative-HPLC, to give 4-(5-bromo-2-hydroxy-benzylamino)-7-chloro-isoindole-1,3-dione (SB-16-124) as the trifluoroacetic acid salt (26 mg, 15%) as a yellow solid. MS: 381 $M+H^+$. ^{1}H NMR (400MHz, cd_3od) δ = 7.37

(dd, *J*=1.2, 9.0 Hz, 1H), 7.32 (s, 1H), 7.19 (d, *J*=8.6 Hz, 1H), 6.96 (d, *J*=8.6 Hz, 1H), 6.72 (d, *J*=8.6 Hz, 1H), 4.45 (s, 2H). HRMS $[M+H]^+$ calculated for $C_{15}H_{10}BrClN_2O_3$: 380.9642; found: 380.9651, err. = 2.4 ppm.

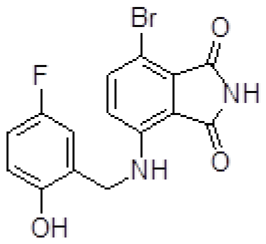

SB-16-67

4-Bromo-7-(5-fluoro-2-hydroxy-benzylamino)-isoindole-1,3-dione (SB-16-67).

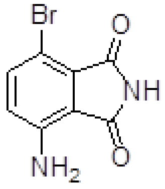

SB-16-45

Synthesis of 4-amino-7-bromo-isoindole-1,3-dione (SB-16-45). To a mixture of 4-amino-isoindole-1,3-dione (1.60 g, 9.87 mmol) in methanol (160 mL) was added N-bromosuccinimide (1.75 g, 9.87 mmol). The reaction was stirred for 5 hours, then was filtered, and the solid was washed with methanol (20 mL), to give 4-amino-7-bromo-isoindole-1,3-dione (SB-16-45) (1.25 g, 53%) as a yellow solid. ^{1}H NMR (300MHz, dmso) δ = 11.08 (br. s., 1H), 7.49 (d, *J*=8.8 Hz, 1H), 6.88 (d, *J*=8.8 Hz, 1H), 6.53 (br. s., 2H).

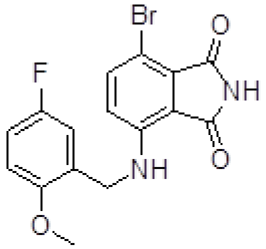

SB-16-58

4-Bromo-7-(5-fluoro-2-methoxy-benzylamino)-isoindole-1,3-dione (SB-16-58). A mixture of 4-amino-7-bromo-isoindole-1,3-dione (241 mg, 1.00 mmol) and 2-bromomethyl-4-fluoro-1-methoxy-benzene (219 mg, 1.00 mmol) in DMF (2 mL) was heated at 100°C for 20 hours. The reaction was poured in to water (30 mL), then the mixture was extracted with 10% methanol in ethyl acetate (2 x 30 mL). The combined organic extracts were

washed with water (2 x 15 mL) and brine (15 mL), dried (Na_2SO_4), and concentrated. The crude material was purified by column on silica (1-10% (5% ammonium hydroxide: methanol): dichloromethane), followed by preparative-HPLC. The product was treated with 10% methanol in ethyl acetate (40 mL), then the mixture was washed with saturated aqueous $NaHCO_3$ (2 x 20 mL), and brine (20 mL), dried (Na_2SO_4), and concentrated, to give 4-bromo-7-(5-fluoro-2-methoxy-benzylamino)-isoindole-1,3-dione (SB-16-58) (47 mg, 12%) as a yellow solid. ^{1}H NMR (400MHz, cdcl$_3$) δ = 7.58 - 7.49 (m, 1H), 7.48 (s, 1H), 7.45 - 7.36 (m, 1H), 7.00 - 6.89 (m, 3H), 6.86 - 6.78 (m, 1H), 6.71 (d, *J*=9.0 Hz, 1H), 4.46 (d, *J*=6.2 Hz, 2H), 3.86 (s, 3H).

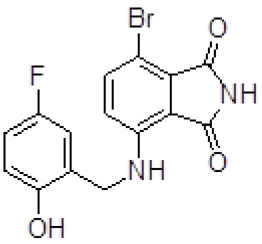

SB-16-67

4-Bromo-7-(5-fluoro-2-hydroxy-benzylamino)-isoindole-1,3-dione (SB-16-67). To a -78ºC mixture of 4-bromo-7-(5-fluoro-2-methoxy-benzylamino)-isoindole-1,3-dione (47 mg, 0.12 mmol) in dichloromethane (1 mL) was added a 1M solution of boron tribromide in dichloromethane (0.36 mL, 0.36 mmol). The reaction was stirred at -78ºC for 30 minutes, and at 0ºC for 20 hours. The reaction was treated with methanol (0.5 mL), saturated aqueous $NaHCO_3$ (20 mL) and 10% methanol in ethyl acetate (30 mL). The organic layer was washed with brine (15 mL), dried (Na_2SO_4), and concentrated. The crude material was purified by preparative-HPLC to give 4-bromo-7-(5-fluoro-2-hydroxy-benzylamino)-isoindole-1,3-dione (SB-16-67) as the trifluoroacetic acid salt (28 mg, 64%) as a yellow solid. ^{1}H NMR (400MHz, dmso) δ = 10.25 (s, 1H), 8.83 (s, 1H), 6.65 (d, *J*=9.0 Hz, 1H), 6.29 (s, 1H), 6.07 (d, *J*=9.4 Hz, 1H), 5.97 (d, *J*=9.0 Hz, 2H), 5.92 - 5.85 (m, 1H), 3.49 (d, *J*=5.9 Hz, 2H). HRMS $[M+H]^+$ calculated for $C_{15}H_{10}BrFN_2O_3$: 364.9937, found: 364.9948, err. = 3.3 ppm.

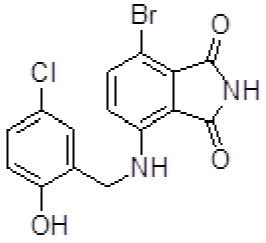

SB-16-51

4-Bromo-7-(5-chloro-2-hydroxy-benzylamino)-isoindole-1,3-dione (SB-16-51).

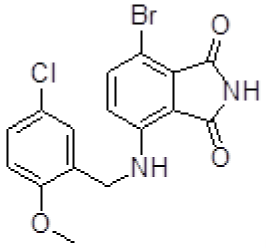

SB-16-48

4-Bromo-7-(5-chloro-2-methoxy-benzylamino)-isoindole-1,3-dione (SB-16-48). A mixture of 4-amino-7-bromo-isoindole-1,3-dione (251 mg, 1.04 mmol) and 2-chloromethyl-4-fluoro-1-methoxy-benzene (245 mg, 1.04 mmol) in DMF (2 mL) was heated at 100ºC for 20 hours. The reaction was poured in to water (30 mL), then the mixture was extracted with 10% methanol in ethyl acetate (2 x 30 mL). The combined organic extracts were washed with water (2 x 20 mL) and brine (20 mL), dried (Na_2SO_4), and concentrated. The crude material was purified by column on silica (1-5% methanol: dichloromethane, followed by preparative-HPLC. The product was treated with 10% methanol in ethyl acetate (50 mL), then the mixture was washed with saturated aqueous $NaHCO_3$ (2 x 20 mL), and brine (20 mL), dried (Na_2SO_4), and concentrated, to give 4-bromo-7-(5-chloro-2-methoxy-benzylamino)-isoindole-1,3-dione (SB-16-48) (48 mg, 12%) as a yellow solid. MS: 396M+H$^+$. ^{1}H NMR (400MHz, dmso) δ = 11.17 (s, 1H), 7.56 (d, *J*=9.0 Hz, 1H), 7.36 - 7.11 (m, 3H), 7.03 (d, *J*=8.6 Hz, 1H), 6.81 (d, *J*=9.0 Hz, 1H), 4.43 (d, *J*=6.6 Hz, 2H), 3.90 - 3.78 (m, 3H).

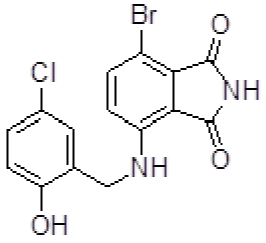

SB-16-51

4-Bromo-7-(5-chloro-2-hydroxy-benzylamino)-isoindole-1,3-dione (SB-16-51). To a -78ºC mixture of 4-bromo-7-(5-chloro-2-methoxy-benzylamino)-isoindole-1,3-dione (SB-16-48) (45 mg, 0.11 mmol) in dichloromethane (0.6 mL) was added a 1M solution of boron tribromide in dichloromethane (0.35 mL, 0.35 mmol). The reaction was stirred at -78ºC for 30 minutes, and at 0ºC for 16 hours. The reaction was treated with methanol (0.5 mL), saturated aqueous $NaHCO_3$ (15 mL), and 10% methanol in ethyl acetate (40 mL). The mixture was shaken, and was filtered through Celite. The filtrate was separated, then the organic layer was washed with brine (20 mL), dried (Na_2SO_4), and concentrated. The crude material was purified by preparative-HPLC to give 4-bromo-7-(5-chloro-2-hydroxy-benzylamino)-isoindole-1,3-dione (SB-16-51) as the trifluoroacetic acid salt (23 mg, 55%) as a yellow solid. ^{1}H NMR (400MHz, dmso) δ = 11.15 (s, 1H), 10.05 (s, 1H), 7.56 (d, *J*=9.0 Hz, 1H), 7.23 - 7.17 (m, 2H), 7.10 (dd, *J*=2.7, 8.6 Hz, 1H), 6.88 (d, *J*=9.0 Hz, 1H), 6.82 (d, *J*=8.6 Hz, 1H), 4.39 (d, *J*=6.2 Hz, 2H). HRMS $[M+H]^+$ calculated for $C_{15}H_{10}BrClN_2O_3$: 380.9642; found: 380.9648, err. = 1.8 ppm.

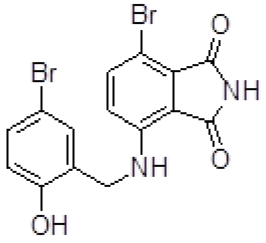

SB-15-167

4-Bromo-7-(5-bromo-2-hydroxy-benzylamino)-isoindole-1,3-dione (SB-15-167).

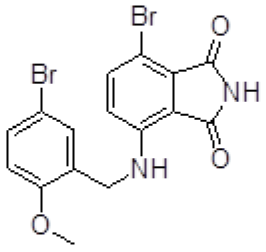

SB-15-157

4-Bromo-7-(5-bromo-2-methoxy-benzylamino)-isoindole-1,3-dione (SB-15-157). A mixture of 4-amino-7-bromo-isoindole-1,3-dione (241 mg, 1.00 mmol) and 4-bromo-2-bromomethyl-1-methoxy-benzene (280 mg, 1.00 mmol) in DMF (2 mL) was heated at 100°C for 20 hours. The reaction was poured in to water (30 mL), then the mixture was extracted with ethyl acetate (60 mL). The organic extract was washed with water (2 x 30 mL) and brine (20 mL), dried (Na_2SO_4), and concentrated. The crude material was purified by column on silica (0-5% methanol: dichloromethane, to give 4-bromo-7-(5-bromo-2-methoxy-benzylamino)-isoindole-1,3-dione (SB-15-157) (273 mg, 62%) as a yellow solid.

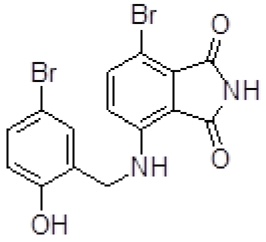

SB-15-167

4-Bromo-7-(5-bromo-2-hydroxy-benzylamino)-isoindole-1,3-dione (SB-15-167). To a -78°C mixture of 4-bromo-7-(5-bromo-2-methoxy-benzylamino)-isoindole-1,3-dione (263 mg, 0.60 mmol) in dichloromethane (3 mL) was added a 1M solution of boron tribromide in dichloromethane (1.80 mL, 1.80 mmol). The reaction was stirred at -78°C for 30 minutes, and at 0°C for 16 hours. The reaction was treated with further dichloromethane (3 mL), and was stirred at 0°C for 13 hours. The reaction was treated with methanol (2.3 mL), saturated aqueous $NaHCO_3$ (33 mL), and ethyl acetate (50 mL). The mixture was shaken, then the organic layer was washed with brine (20 mL), dried (Na_2SO_4), and concentrated. The crude material was purified by column on silica (0-100% ethyl acetate: hexanes), to give 4-bromo-7-(5-bromo-2-hydroxy-benzylamino)-isoindole-1,3-dione (SB-15-167) (91 mg, 36%) as a yellow solid. ^{1}H NMR (300MHz, dmso) δ = 11.17 (br. s., 1H), 10.09 (s, 1H), 7.58 (d, *J*=9.4

Hz, 1H), 7.33 (d, *J*=2.3 Hz, 1H), 7.28 - 7.16 (m, 2H), 6.90 (d, *J*=8.8 Hz, 1H), 6.79 (d, *J*=8.8 Hz, 1H), 4.40 (d, *J*=6.4 Hz, 2H).

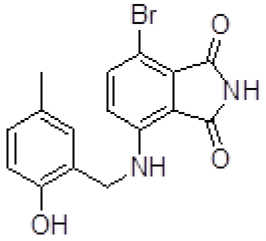

SB-16-122

4-Bromo-7-(2-hydroxy-5-methyl-benzylamino)-isoindole-1,3-dione (SB-16-122).

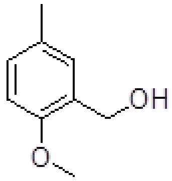

SB-16-74

(2-Methoxy-5-methyl-phenyl)-methanol (SB-16-74). To a 0ºC mixture of 2-methoxy-5-methyl-benzoic acid (2.00 g, 12.04 mmol) in THF (28 mL) was added dropwise a 2M solution of BH_3 . SMe_2 in THF (12.0 mL, 24.00 mmol). The reaction was allowed to warm to 20ºC, and was stirred for 3 days. The reaction was cooled to 0ºC, and water (100 mL) was slowly added. The mixture was treated with brine (50 mL), then was extracted with ethyl acetate (2 x 50 mL). The combined organic extracts were washed with saturate aqueous $NaHCO_3$ (30 mL), and brine (25 mL), dried (Na_2SO_4), and concentrated. The crude material was purified by column on silica (0-40% ethyl acetate: hexanes), to give (2-methoxy-5-methyl-phenyl)-methanol (SB-16-74) (1.64 g, 90%) as a clear liquid. ^{1}H NMR (400MHz, dmso) δ = 7.15 (s, 1H), 6.97 (dd, *J*=1.6, 8.2 Hz, 1H), 6.79 (d, *J*=8.6 Hz, 1H), 4.89 (t, *J*=5.7 Hz, 1H), 4.43 (d, *J*=5.9 Hz, 2H), 3.70 (s, 3H), 2.22 (s, 3H).

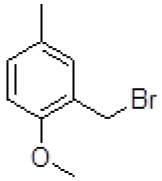

SB-16-82

2-Bromomethyl-1-methoxy-4-methyl-benzene (SB-16-82). To a mixture of (2-methoxy-5-methyl-phenyl)-methanol (238 mg, 1.57 mmol) in diethyl ether (1 mL) was added PBr_3 (82 mL, 0.87 mmol). The reaction was

stirred for 3.5 hours, then water (15 mL) was added. The mixture was extracted with ethyl acetate (2 x 25 mL). The combined organic extracts were washed with brine (15 mL), dried (Na_2SO_4), and concentrated to give 2-bromomethyl-1-methoxy-4-methyl-benzene (SB-16-82) (322 mg, 95%) as a pale orange liquid. ^{1}H NMR (400MHz, cdcl$_3$) δ = 7.26 (s, 1H), 7.14 (d, *J*=2.0 Hz, 1H), 7.09 (dd, *J*=1.8, 8.4 Hz, 1H), 6.78 (d, *J*=8.2 Hz, 1H), 4.63 - 4.45 (m, 2H), 3.95 - 3.81 (m, 3H), 2.30 - 2.23 (m, 3H).

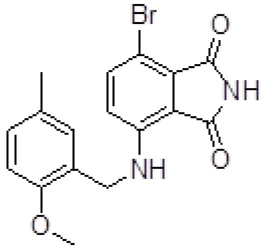

SB-16-83

4-Bromo-7-(2-methoxy-5-methyl-benzylamino)-isoindole-1,3-dione (SB-16-83). A mixture of 4-amino-7-bromo-isoindole-1,3-dione (361 mg, 1.50 mmol) and 2-bromomethyl-1-methoxy-4-methyl-benzene (322 mg, 1.50 mmol) in DMF (3 mL) was heated at 100ºC for 20 hours. The reaction was poured in to a mixture of water (23 mL) and saturated aqueous $NaHCO_3$ (23 mL), then the mixture was extracted with 10% methanol in ethyl acetate (2 x 50 mL). The organic extract was washed with water (2 x 20 mL) and brine (15 mL), dried (Na_2SO_4), and concentrated. The crude material was purified by column on silica (0-5% methanol: dichloromethane, followed by preparative-HPLC. The product was treated with 10% methanol in ethyl acetate (80 mL), then the mixture was washed with saturated aqueous $NaHCO_3$ (2 x 20 mL), and brine (20 mL), dried (Na_2SO_4), and concentrated to give 4-bromo-7-(2-methoxy-5-methyl-benzylamino)-isoindole-1,3-dione (SB-16-83) (183 mg, 33%) as a yellow solid. ^{1}H NMR (400MHz, dmso) δ = 11.14 (br. s., 1H), 7.55 (d, *J*=9.0 Hz, 1H), 7.16 - 6.77 (m, 5H), 4.40 (d, *J*=6.2 Hz, 2H), 3.78 (s, 3H), 2.25 - 2.03 (m, 3H).

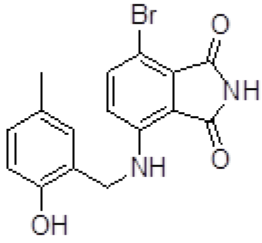

SB-16-122

4-Bromo-7-(2-hydroxy-5-methyl-benzylamino)-isoindole-1,3-dione (SB-16-122). To a -78°C mixture of 4-bromo-7-(2-methoxy-5-methyl-benzylamino)-isoindole-1,3-dione (183 mg, 0.49 mmol) in dichloromethane (2.5 mL) was added a 1M solution of boron tribromide in dichloromethane (1.50 mL, 1.50 mmol). The reaction was stirred at -78°C for 30 minutes, and at 0°C for 16 hours. The reaction was treated with methanol (2.0 mL), saturated aqueous $NaHCO_3$ (30 mL), and 10% methanol in ethyl acetate (60 mL). The mixture was shaken, then the organic layer was washed with brine (20 mL), dried (Na_2SO_4), and concentrated. The crude material was purified by preparative-HPLC, to give 4-bromo-7-(2-hydroxy-5-methyl-benzylamino)-isoindole-1,3-dione (SB-16-122) as the trifluoroacetic acid salt (51 mg, 22%) as a yellow solid. ^1H NMR (400MHz, dmso) δ = 10.73 - 10.44 (m, 1H), 8.95 (br. s., 1H), 7.03 (br. s., 1H), 6.55 (br. s., 1H), 6.48 - 6.26 (m, 3H), 6.17 (br. s., 1H), 3.83 (br. s., 2H), 1.96 (br. s., 5H), 1.69 - 1.45 (m, 3H). HRMS $[M+H]^+$ calculated for $C_{16}H_{13}BrN_2O_3$: 361.0188; found: 261.0190, err. = 0.6 ppm.

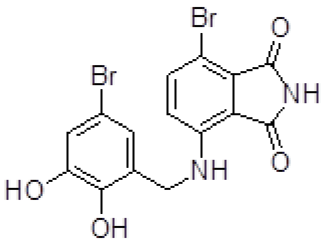

SB-16-250

4-Bromo-7-(5-bromo-2,3-dihydroxy-benzylamino)-isoindole-1,3-dione (SB-16-250).

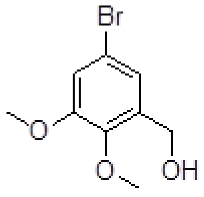

SB-16-212

(5-Bromo-2,3-dimethoxy-phenyl)-methanol (SB-16-212). To a 0°C of 5-bromo-2,3-dimethoxy-benzaldehyde (0.75 g, 3.06 mmol) in DMF (6 mL) was added $NaBH_4$ (163 mg, 4.28 mmol). The reaction was allowed to warm to 20°C, and was stirred for 3 hours. The reaction was poured in to a mixture of 1N HCl (30 mL) and water (60 mL). The mixture was extracted with ethyl acetate (2 x 50 mL). The combined organic extracts were washed with water (2 x 25 mL), and brine (20 mL), dried (Na_2SO_4), and concentrated, to give (5-bromo-2,3-dimethoxy-

phenyl)-methanol (SB-16-212) (0.71 g, 94%) as an off-white solid. ^{1}H NMR (400MHz, cdcl$_3$) δ = 7.08 (d, *J*=2.0 Hz, 1H), 6.97 (d, *J*=2.3 Hz, 1H), 4.63 (s, 2H), 3.91 - 3.72 (m, 6H), 2.36 (s, 1H).

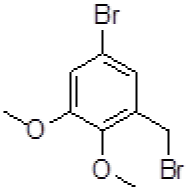

SB-16-234

5-Bromo-1-bromomethyl-2,3-dimethoxy-benzene (SB-16-234). To a 0°C mixture of (5-bromo-2,3-dimethoxy-phenyl)-methanol (0.71 g, 2.87 mmol) in dichloromethane (8.3 mL) was added PBr_3 (1.17 g, 404 mL, 4.31 mmol). The reaction was allowed to warm to 20°C, and was stirred for 3 hours. The reaction was treated with water (14 mL), then the mixture was extracted with dichloromethane (2 x 30 mL). The combined organic extracts were washed with brine (20 mL), dried (Na_2SO_4), and concentrated. The residue was treated with dichloromethane (8.3 mL), then was cooled to 0°C, and PBr_3 (1.17 g, 404 mL, 4.31 mmol) was added. The reaction was allowed to warm to 20°C, and was stirred for 16 hours. The reaction was treated with water (14 mL), then the mixture was extracted with dichloromethane (2 x 30 mL). The combined organic extracts were washed with brine (20 mL), dried (Na_2SO_4), and concentrated, to give 5-bromo-1-bromomethyl-2,3-dimethoxy-benzene (SB-16-234) (0.78 g, 88%) as a white solid. ^{1}H NMR (400MHz, cdcl$_3$) δ = 7.13 - 6.93 (m, 2H), 4.46 (s, 2H), 3.92 (s, 3H), 3.84 (s, 3H).

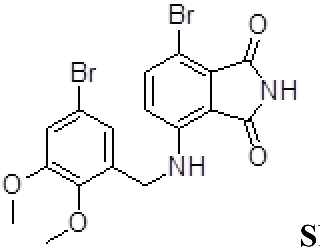

SB-16-238

4-Bromo-7-(5-bromo-2,3-dimethoxy-benzylamino)-isoindole-1,3-dione (SB-16-238). A mixture of 4-amino-7-bromo-isoindole-1,3-dione (362 mg, 1.50 mmol) and 5-bromo-1-bromomethyl-2,3-dimethoxy-benzene (465 mg, 1.50 mmol) in DMF (3 mL) was heated at 100°C for 24 hours. The reaction was poured in to water (45 mL), then the mixture was extracted with 10% methanol in ethyl acetate (2 x 125 mL). The combined organic extracts were treated with water (25 mL0, then the mixture was shaken and was filtered through Celite. The filtrate was separated, then the organic layer was washed with water (25 mL), and brine (20 mL), dried (Na_2SO_4), and

concentrated. The crude material was purified by column on silica (1-10% (5% ammonium hydroxide in methanol)/ dichloromethane), followed by preparative-HPLC. The product was treated with 10% methanol in dichloromethane (100 mL), then was washed with saturate aqueous $NaHCO_3$ (2 x 25 mL), and brine (20 mL), dried (Na_2SO_4), and concentrated, to give 4-bromo-7-(5-bromo-2,3-dimethoxy-benzylamino)-isoindole-1,3-dione (SB-16-238) (65 mg, 9%) as a yellow solid. ^{1}H NMR (400MHz, dmso) δ = 7.58 (d, *J*=9.0 Hz, 1H), 7.25 - 7.11 (m, 2H), 7.02 (d, *J*=2.3 Hz, 1H), 6.86 (d, *J*=9.0 Hz, 1H), 4.47 (d, *J*=6.2 Hz, 2H), 3.81 (s, 3H), 3.76 (s, 3H).

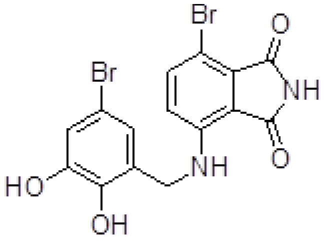

SB-16-250

4-Bromo-7-(5-bromo-2,3-dihydroxy-benzylamino)-isoindole-1,3-dione (SB-16-250). To a -78 ºC mixture of 4-bromo-7-(5-bromo-2,3-dimethoxy-benzylamino)-isoindole-1,3-dione (62 mg, 0.13 mmol) in dichloromethane (0.66 mL) was added a 1M solution of boron tribromide in dichloromethane (0.77 mL, 0.77 mmol). The reaction was stirred at -78ºC for 30 minutes, and at 0ºC for 16 hours. The reaction was treated with methanol (1.2 mL), saturated aqueous $NaHCO_3$ (15 mL), and 10% methanol in ethyl acetate (30 mL). The mixture was shaken, then the organic layer was washed with brine (15 mL), dried (Na_2SO_4), and concentrated. The crude material was purified by preparative-HPLC, to give 4-bromo-7-(5-bromo-2,3-dihydroxy-benzylamino)-isoindole-1,3-dione (SB-16-250) as the trifluoroacetic acid salt (44 mg, 61%) as a yellow solid. ^{1}H NMR (400MHz, dmso) δ = 11.16 (s, 1H), 9.99 - 9.81 (m, 1H), 8.97 (s, 1H), 7.56 (d, *J*=9.0 Hz, 1H), 7.15 (t, *J*=6.4 Hz, 1H), 6.86 (d, *J*=9.0 Hz, 1H), 6.80 (q, *J*=2.3 Hz, 2H), 4.38 (d, *J*=6.2 Hz, 2H). HRMS $[M+H]^+$ calculated for $C_{15}H_{10}Br_2N_2O_4$: 440.9086; found: 440.9081, err. = -1.1 ppm.

Compounds in Table S4.

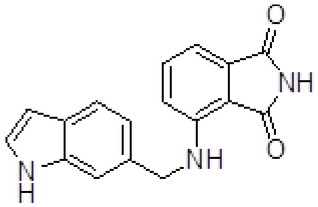

SB-17-75

4-[(1H-Indol-6-ylmethyl)-amino]-isoindole-1,3-dione (SB-17-75). The compound was synthesized following method B, using 1H-indole-6-carbaldehyde (436 mg, 3.00 mmol), 4-amino-isoindole-1,3-dione (243 mg, 1.50 mmol), DMF (4.5 mL), $Ti(O^iPr)_4$ (0.93 g, 0.97 mL, 3.00 mmol), and $NaBH_4$ (117 mg, 3.13 mmol). The reaction was stirred for 3 days. The reaction was poured in to a mixture of water (34 mL) and saturated aqueous $NaHCO_3$ (34 mL). The mixture was treated with 10% methanol in ethyl acetate (200 mL), then was shaken, and was filtered through Celite. The filtrate was separated, then the organic layer was washed with water (2 x 25 mL), and brine (20 mL), dried (Na_2SO_4), and concentrated. The crude material was purified by column on silica (1-15% (5% ammonium hydroxide in methanol)/dichloromethane, followed by preparative-HPLC, to give 4-[(1H-indol-6-ylmethyl)-amino]-isoindole-1,3-dione (SB-17-75) as the bis-trifluoroacetic acid salt (68 mg, 9%) as a yellow solid. ^{1}H NMR (400MHz, dmso) δ = 10.99 (br. s., 1H), 7.51 - 7.39 (m, 2H), 7.35 (s, 1H), 7.31 - 7.25 (m, 1H), 7.06 (t, *J*=5.9 Hz, 1H), 7.00 (d, *J*=8.2 Hz, 1H), 6.95 (d, *J*=8.6 Hz, 1H), 6.89 (d, *J*=7.0 Hz, 1H), 6.45 - 6.29 (m, 1H), 4.58 (d, *J*=5.9 Hz, 2H). HRMS $[M-H]^+$ calculated for $C_{17}H_{13}N_2O_3$ 290.0930; found: 290.0928, err. = -0.7 ppm.

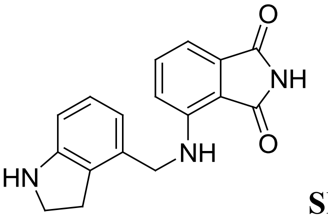

SB-17-140

4-[(2,3-Dihydro-1H-indol-4-ylmethyl)-amino]-isoindole-1,3-dione (SB-17-140).

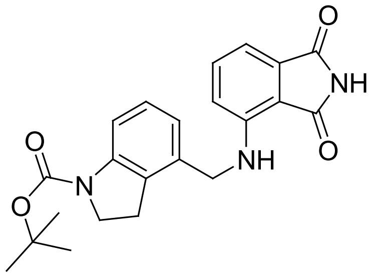

SB-17-128

4-[(1,3-Dioxo-2,3-dihydro-1H-isoindol-4-ylamino)-methyl]-2,3-dihydro-indole-1-carboxylic acid tert-butyl ester (SB-17-128). The compound was synthesized following general procedure C, using 4-formyl-2,3-dihydro-indole-1-carboxylic acid tert-butyl ester (742 mg, 3.00 mmol), 4-amino-isoindole-1,3-dione (243 mg, 1.50 mmol), DMF (4.5 mL), $Ti(O^iPr)_4$ (1.24 g, 1.29 mL, 4.00 mmol) and $NaBH_4$ (117 mg, 3.12 mmol). The reaction was stirred for 16 hours. The reaction was poured in to a mixture of water (34 mL) and saturated aqueous $NaHCO_3$ (34 mL). The mixture was treated with 10% methanol in ethyl acetate (200 mL), then was shaken, and was filtered through Celite. The filtrate was separated, then the organic layer was washed with water (2 x 25 mL), and brine (20 mL), dried (Na_2SO_4), and concentrated. The crude material was purified by column on silica (1-15% (5% ammonium hydroxide in methanol)/dichloromethane, followed by preparative-HPLC. The product was treated with 10% methanol in ethyl acetate (70 mL), then the mixture was washed with saturated aqueous $NaHCO_3$ (2 x 25 mL) and brine (20 mL), to give 4-[(1,3-dioxo-2,3-dihydro-1H-isoindol-4-ylamino)-methyl]-2,3-dihydro-indole-1-carboxylic acid tert-butyl ester (SB-17-128) (144 mg, 24%) as a yellow solid. ^{1}H NMR (400MHz, dmso) δ = 10.96 (s, 1H), 7.44 (dd, *J*=7.2, 8.4 Hz, 1H), 7.12 - 7.06 (m, 1H), 7.03 - 6.95 (m, 1H), 6.91 (d, *J*=7.0 Hz, 1H), 6.87 - 6.81 (m, 2H), 4.44 (d, *J*=6.2 Hz, 2H), 3.92 (t, *J*=8.8 Hz, 2H), 3.07 (t, *J*=8.4 Hz, 2H), 1.48 (s, 9H).

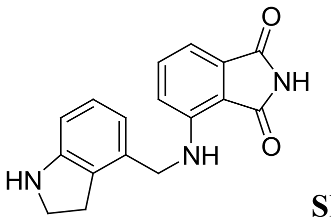

SB-17-140

4-[(2,3-Dihydro-1H-indol-4-ylmethyl)-amino]-isoindole-1,3-dione (SB-17-140). To a mixture of 4-[(1,3-dioxo-2,3-dihydro-1H-isoindol-4-ylamino)-methyl]-2,3-dihydro-indole-1-carboxylic acid tert-butyl ester (139 mg, 0.35 mmol) in dichloromethane (1.8 mL) was added trifluoroacetic acid (1.8 mL). The reaction was stirred for 16 hours, then was concentrated. The product was lyophilized, to give 4-[(2,3-Dihydro-1H-indol-4-ylmethyl)-amino]-isoindole-1,3-dione (SB-17-140) as the bis-trifluoroacetic acid salt (171 mg, 94%) as a yellow solid. ^{1}H NMR (400MHz, dmso) δ = 10.98 (s, 1H), 7.44 (dd, *J*=7.2, 8.4 Hz, 1H), 7.19 - 7.12 (m, 1H), 7.07 - 6.95 (m, 3H), 6.92 (d, *J*=7.0 Hz, 1H), 6.86 (d, *J*=8.2 Hz, 1H), 4.61 - 4.39 (m, 2H), 3.71 - 3.56 (m, 2H), 3.21 - 3.02 (m, 2H). HRMS calculated for $C_{17}H_{15}N_3O_2$: 294.1243; found 294.1248, err. = 1.7 ppm.

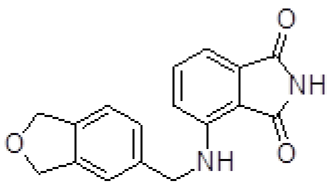

SB-17-80

4-[(1,3-Dihydro-isobenzofuran-5-ylmethyl)-amino]-isoindole-1,3-dione (SB-17-80). The compound was synthesized following method B, using 1,3-dihydro-isobenzofuran-5-carbaldehyde (445 mg, 3.00 mmol), 4-amino-isoindole-1,3-dione (243 mg, 1.50 mmol), DMF (4.5 mL), $Ti(O^iPr)_4$ (0.93 g, 0.97 mL, 3.00 mmol) and $NaBH_4$ (117 mg, 3.13 mmol). The reaction was stirred for 16 hours. The reaction was poured in to a mixture of water (34 mL) and saturated aqueous $NaHCO_3$ (34 mL). The mixture was treated with 10% methanol in ethyl acetate (200 mL), then was shaken, and was filtered through Celite. The filtrate was separated, then the organic layer was washed with water (2 x 25 mL), and brine (20 mL), dried (Na_2SO_4), and concentrated. The crude material was purified by column on silica (1-15% (5% ammonium hydroxide in methanol)/dichloromethane, followed by preparative-HPLC, to give 4-[(1,3-dihydro-isobenzofuran-5-ylmethyl)-amino]-isoindole-1,3-dione (SB-17-80) as the trifluoroacetic acid salt (26 mg, 4%) as a yellow solid. ^{1}H NMR (400MHz, dmso) δ = 10.07 - 9.04 (m, 1H), 7.41 (t, *J*=7.6 Hz, 1H), 7.33 - 7.19 (m, 3H), 7.08 (br. s., 1H), 6.96 - 6.75 (m, 2H), 5.04 - 4.83 (m, 4H), 4.49 (d, *J*=5.5 Hz, 2H). HRMS [M+H]+ calculated for $C_{17}H_{14}N_2O_3$ 295.1083; found: 295.1088, err. = 1.7 ppm.

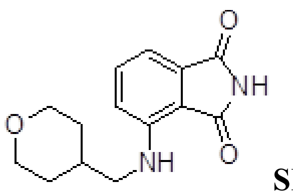

SB-16-237

4-[(Tetrahydro-pyran-4-ylmethyl)-amino]-isoindole-1,3-dione (SB-16-237). The compound was synthesized following general procedure C, using tetrahydro-pyran-4-carbaldehyde (571 mg, 5.00 mmol), 4-amino-isoindole-1,3-dione (405 mg, 2.50 mmol), DMF (7.5 mL), $Ti(O^iPr)_4$ (1.55 g, 1.62 mL, 5.00 mmol), and $NaBH_4$ (195 mg, 5.20 mmol). The reaction was stirred for 16 hours. The reaction was poured in to a mixture of water (55 mL) and saturated aqueous $NaHCO_3$ (55 mL). The mixture was treated with 10% methanol in ethyl acetate (250 mL), then was shaken, and was filtered through Celite. The filtrate was separated, then the organic layer was washed with water (2 x 25 mL), and brine (20 mL), dried (Na_2SO_4), and concentrated. The crude material was purified by column on silica (1-20% (5% ammonium hydroxide in methanol)/dichloromethane, followed by preparative-HPLC, to give 4-[(tetrahydro-pyran-4-ylmethyl)-amino]-isoindole-1,3-dione (SB-16-237) as the trifluoroacetic acid salt (124 mg, 13%) as a yellow solid. ^1H NMR (400MHz, dmso) δ = 11.14 - 10.79 (m, 1H), 7.51 (dd, *J*=7.2, 8.4 Hz, 1H), 7.05 (d, *J*=8.6 Hz, 1H), 6.91 (d, *J*=7.0 Hz, 1H), 6.54 (t, *J*=6.2 Hz, 1H), 3.83 (dd, *J*=2.7, 11.3 Hz, 3H), 3.32 - 3.13 (m, 7H), 1.92 - 1.75 (m, 1H), 1.59 (dd, *J*=1.8, 12.7 Hz, 3H), 1.35 - 1.02 (m, 3H). HRMS $[M+H]^+$ calculated for $C_{14}H_{16}N_2O_3$: 261.1239; found: 261.1241, err. = 0.8 ppm.

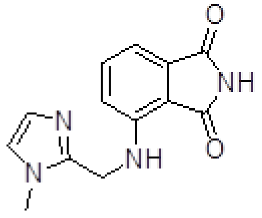

SB-17-95

4-[(1-Methyl-1H-imidazol-2-ylmethyl)-amino]-isoindole-1,3-dione (SB-17-95). The compound was synthesized following general procedure C, using 1-methyl-1H-imidazole-2-carbaldehyde (330 mg, 3.00 mmol), 4-amino -isoindole-1,3-dione (243 mg, 1.50 mmol), DMF (4.5 mL), $Ti(O^iPr)_4$ (0.93 g, 0.97 mL, 3.00 mmol), and

$NaBH_4$ (117 mg, 3.12 mmol). The reaction was stirred for 16 hours. The reaction was poured in to a mixture of water (34 mL) and saturated aqueous $NaHCO_3$ (34 mL). The mixture was treated with 10% methanol in ethyl acetate (200 mL), then was shaken, and was filtered through Celite. The filtrate was separated, then the organic layer was washed with water (2 x 25 mL), and brine (20 mL), dried (Na_2SO_4), and concentrated. The crude material was purified by column on silica (1-20% (5% ammonium hydroxide in methanol)/dichloromethane, followed by preparative-HPLC, to give 4-[(1-methyl-1H-imidazol-2-ylmethyl)-amino]-isoindole-1,3-dione (SB-17-95) as the bis-trifluoroacetic acid salt (102 mg, 14%) as a light yellow solid. ^{1}H NMR (400MHz, dmso) δ = 11.18 - 10.85 (m, 1H), 7.60 - 7.55 (m, 1H), 7.50 - 7.41 (m, 2H), 7.22 - 7.13 (m, 1H), 7.02 - 6.91 (m, 2H), 4.83 (d, *J*=6.2 Hz, 2H). HRMS $[M+H]^+$ calculated for $C_{13}H_{12}N_4O_2$:257.1039; found: 257.1036, err. = -1.2 ppm.

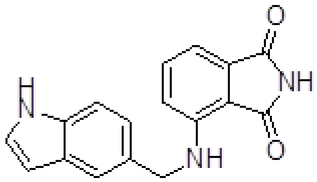

SB-17-16

4-[(1H-Indol-5-ylmethyl)-amino]-isoindole-1,3-dione (SB-17-16). The compound was synthesized following method B, using 1H-indole-5-carbaldehyde (581 mg, 4.00 mmol), 4-amino-isoindole-1,3-dione (324 mg, 2.00 mmol), DMF (6 mL), $Ti(O^iPr)_4$ (1.24 g, 1.29 mL, 4.00 mmol), and $NaBH_4$ (156 mg, 4.16 mmol). The reaction was stirred for 12 days. The reaction was poured in to a mixture of water (45 mL) and saturated aqueous $NaHCO_3$ (45 mL). The mixture was treated with 10% methanol in ethyl acetate (250 mL), then was shaken, and was filtered through Celite. The filtrate was separated, then the organic layer was washed with water (2 x 25 mL), and brine (20 mL), dried (Na_2SO_4), and concentrated. The crude material was purified by column on silica (1-20% (5% ammonium hydroxide in methanol)/dichloromethane, followed by preparative-HPLC, to give 4-[(1H-indol-5-ylmethyl)-amino]-isoindole-1,3-dione (SB-17-16) as the bis-trifluoroacetic acid salt (255 mg, 25%) as a yellow solid. ^{1}H NMR (400MHz, dmso) δ = 11.04 (br. s., 1H), 10.93 (s, 1H), 7.50 (s, 1H), 7.43 (dd, *J*=7.2, 8.4 Hz, 1H), 7.33 (d, *J*=8.2 Hz, 1H), 7.29 (t, *J*=2.5 Hz, 1H), 7.08 (dd, *J*=1.4, 8.4 Hz, 1H), 7.00 - 6.95 (m, 2H), 6.88 (d, *J*=7.0 Hz, 1H), 6.35 (br. s., 1H), 4.53 (d, *J*=5.5 Hz, 2H).

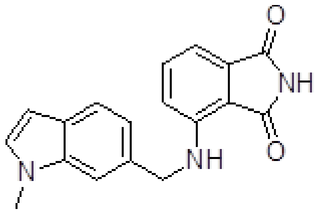

SB-17-114

4-[(1-Methyl-1H-indol-6-ylmethyl)-amino]-isoindole-1,3-dione (SB-17-114). The compound was synthesized following method B, using 1-methyl-1H-indole-6-carbaldehyde (478 mg, 3.00 mmol), 4-amino-isoindole-1,3-dione (243 mg, 1.50 mmol), DMF (4.5 mL), $Ti(O^iPr)_4$ (0.93 g, 0.97 mL, 3.00 mmol), and $NaBH_4$ (117 mg, 3.13 mmol). The reaction was stirred for 3 days. The reaction was poured in to a mixture of water (34 mL) and saturated aqueous $NaHCO_3$ (34 mL). The mixture was treated with 10% methanol in ethyl acetate (200 mL), then was shaken, and was filtered through Celite. The filtrate was separated, then the organic layer was washed with water (2 x 25 mL), and brine (20 mL), dried (Na_2SO_4), and concentrated. The crude material was purified by column on silica (1-15% (5% ammonium hydroxide in methanol)/dichloromethane, followed by preparative-HPLC, to give 4-[(1-methyl-1H-indol-6-ylmethyl)-amino]-isoindole-1,3-dione (SB-17-114) as the bis-trifluoroacetic acid salt (47 mg, 6%) as a yellow solid. ^1H NMR (400MHz, dmso) δ = 11.13 - 10.66 (m, 1H), 7.58 - 7.37 (m, 3H), 7.27 (d, *J*=3.1 Hz, 1H), 7.04 (dd, *J*=1.4, 8.0 Hz, 1H), 7.00 (d, *J*=8.2 Hz, 2H), 6.90 (d, *J*=6.6 Hz, 1H), 6.36 (dd, *J*=0.8, 3.1 Hz, 1H), 4.58 (d, *J*=5.9 Hz, 2H).

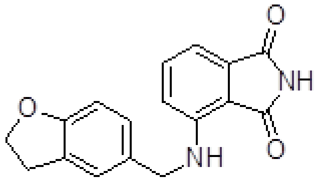

SB-17-98

4-[(2,3-Dihydro-benzofuran-5-ylmethyl)-amino]-isoindole-1,3-dione (SB-17-98). The compound was synthesized following method B, using 2,3-dihydro-benzofuran-5-carbaldehyde (445 mg, 3.00 mmol), 4-amino-isoindole-1,3-dione (243 mg, 1.50 mmol), DMF (4.5 mL), $Ti(O^iPr)_4$ (0.93 g, 0.97 mL, 3.00 mmol) and $NaBH_4$ (117 mg, 3.13 mmol). The reaction was stirred for 16 hours. The reaction was poured in to a mixture of water (34

mL) and saturated aqueous $NaHCO_3$ (34 mL). The mixture was treated with 10% methanol in ethyl acetate (200 mL), then was shaken, and was filtered through Celite. The filtrate was separated, then the organic layer was washed with water (2 x 25 mL), and brine (20 mL), dried (Na_2SO_4), and concentrated. The crude material was purified by column on silica (1-15% (5% ammonium hydroxide in methanol)/dichloromethane, followed by preparative-HPLC, to give 4-[(2,3-dihydro-benzofuran-5-ylmethyl)-amino]-isoindole-1,3-dione (SB-17-98) as the trifluoroacetic acid salt (26 mg, 4%) as a yellow solid. ^{1}H NMR (400MHz, dmso) δ = 10.58 - 9.92 (m, 1H), 7.44 (t, *J*=7.8 Hz, 1H), 7.21 (s, 1H), 7.10 - 7.03 (m, 1H), 6.99 - 6.87 (m, 3H), 6.78 - 6.72 (m, 1H), 6.68 (d, *J*=8.2 Hz, 1H), 4.61 - 4.27 (m, 4H), 3.22 - 3.02 (m, 2H).

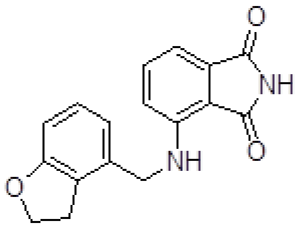

SB-17-110

4-[(2,3-Dihydro-benzofuran-4-ylmethyl)-amino]-isoindole-1,3-dione (SB-17-110). The compound was synthesized following general procedure C, using 2,3-dihydro-benzofuran-4-carbaldehyde (445 mg, 3.00 mmol), 4-amino-isoindole-1,3-dione (243 mg, 1.50 mmol), DMF (4.5 mL), $Ti(O^iPr)_4$ (0.93 g, 0.97 mL, 3.00 mmol), and $NaBH_4$ (117 mg, 3.13 mmol). The reaction was stirred for 16 hours. The reaction was poured in to a mixture of water (34 mL) and saturated aqueous $NaHCO_3$ (34 mL). The mixture was treated with 10% methanol in ethyl acetate (200 mL), then was shaken, and was filtered through Celite. The filtrate was separated, then the organic layer was washed with water (2 x 25 mL), and brine (20 mL), dried (Na_2SO_4), and concentrated. The crude material was purified by column on silica (1-15% (5% ammonium hydroxide in methanol)/dichloromethane, followed by preparative-HPLC, to give 4-[(2,3-dihydro-benzofuran-4-ylmethyl)-amino]-isoindole-1,3-dione (SB-17-110) as the trifluoroacetic acid salt (57 mg, 9%) as a yellow solid. ^{1}H NMR (400MHz, dmso) δ = 11.07 - 10.81 (m, 1H), 7.45 (dd, *J*=7.2, 8.4 Hz, 1H), 7.05 - 6.96 (m, 3H), 6.95 - 6.84 (m, 3H), 6.80 - 6.72 (m, 1H), 6.67 - 6.60 (m, 1H), 4.62 - 4.38 (m, 6H), 2.48 (td, *J*=1.9, 3.6 Hz, 4H).

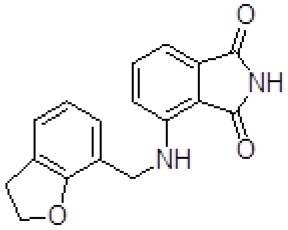

SB-17-112

4-[(2,3-Dihydro-benzofuran-7-ylmethyl)-amino]-isoindole-1,3-dione (SB-17-112). The compound was synthesized following general procedure C, using ,3-dihydro-benzofuran-7-carbaldehyde (445 mg, 3.00 mmol), 4-amino-isoindole-1,3-dione (243 mg, 1.50 mmol), DMF (4.5 mL), $Ti(O^iPr)_4$ (0.93 g, 0.97 mL, 3.00 mmol), and $NaBH_4$ (117 mg, 3.13 mmol). The reaction was stirred for 16 hours. The reaction was poured in to a mixture of water (34 mL) and saturated aqueous $NaHCO_3$ (34 mL). The mixture was treated with 10% methanol in ethyl acetate (200 mL), then was shaken, and was filtered through Celite. The filtrate was separated, then the organic layer was washed with water (2 x 25 mL), and brine (20 mL), dried (Na_2SO_4), and concentrated. The crude material was purified by column on silica (1-15% (5% ammonium hydroxide in methanol)/dichloromethane, followed by preparative-HPLC, to give 4-[(2,3-dihydro-benzofuran-7-ylmethyl)-amino]-isoindole-1,3-dione (SB-17-112) as the trifluoroacetic acid salt (59 mg, 10%) as a yellow solid. ^{1}H NMR (400MHz, dmso) δ = 11.00 - 10.85 (m, 1H), 11.12 - 10.77 (m, 1H), 7.46 (dd, *J*=7.2, 8.4 Hz, 1H), 7.14 - 7.09 (m, 1H), 7.03 (d, *J*=7.8 Hz, 1H), 6.97 - 6.88 (m, 3H), 6.75 (t, *J*=7.4 Hz, 1H), 4.56 (t, *J*=8.8 Hz, 2H), 4.40 (d, *J*=5.9 Hz, 2H), 3.30 (s, 6H), 3.17 (t, *J*=8.8 Hz, 2H).

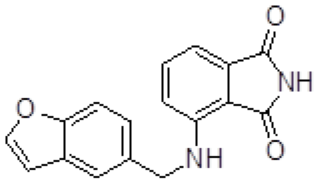

SB-17-109

4-[(Benzofuran-5-ylmethyl)-amino]-isoindole-1,3-dione (SB-17-109). The compound was synthesized following method B, using benzofuran-5-carbaldehyde (438 mg, 3.00 mmol), 4-amino-isoindole-1,3-dione (243 mg, 1.50 mmol), DMF (4.5 mL), $Ti(O^iPr)_4$ (0.93 g, 0.97 mL, 3.00 mmol), and $NaBH_4$ (117 mg, 3.13 mmol). The

reaction was stirred for 16 hours. The reaction was poured in to a mixture of water (34 mL) and saturated aqueous $NaHCO_3$ (34 mL). The mixture was treated with 10% methanol in ethyl acetate (200 mL), then was shaken, and was filtered through Celite. The filtrate was separated, then the organic layer was washed with water (2 x 25 mL), and brine (20 mL), dried (Na_2SO_4), and concentrated. The crude material was purified by column on silica (1-15% (5% ammonium hydroxide in methanol)/dichloromethane, followed by preparative-HPLC, to give 4-[(benzofuran-5-ylmethyl)-amino]-isoindole-1,3-dione (SB-17-109) as the trifluoroacetic acid salt (62 mg, 10%) as a yellow solid. ^{1}H NMR (400MHz, dmso) δ = 11.12 - 10.42 (m, 1H), 10.83 (br. s., 1H), 7.94 (d, *J*=2.3 Hz, 1H), 7.62 (d, *J*=0.8 Hz, 1H), 7.54 (d, *J*=8.2 Hz, 1H), 7.43 (dd, *J*=7.2, 8.4 Hz, 1H), 7.31 (dd, *J*=1.6, 8.6 Hz, 1H), 7.13 (t, *J*=6.2 Hz, 1H), 6.98 - 6.78 (m, 3H), 4.59 (d, *J*=6.2 Hz, 2H).

^{1}H and ^{13}C spectra for 4-((5-Chloro-2-hydroxybenzyl)amino)isoindoline-1,3-dione (KM-5-25)

^{1}H NMR:

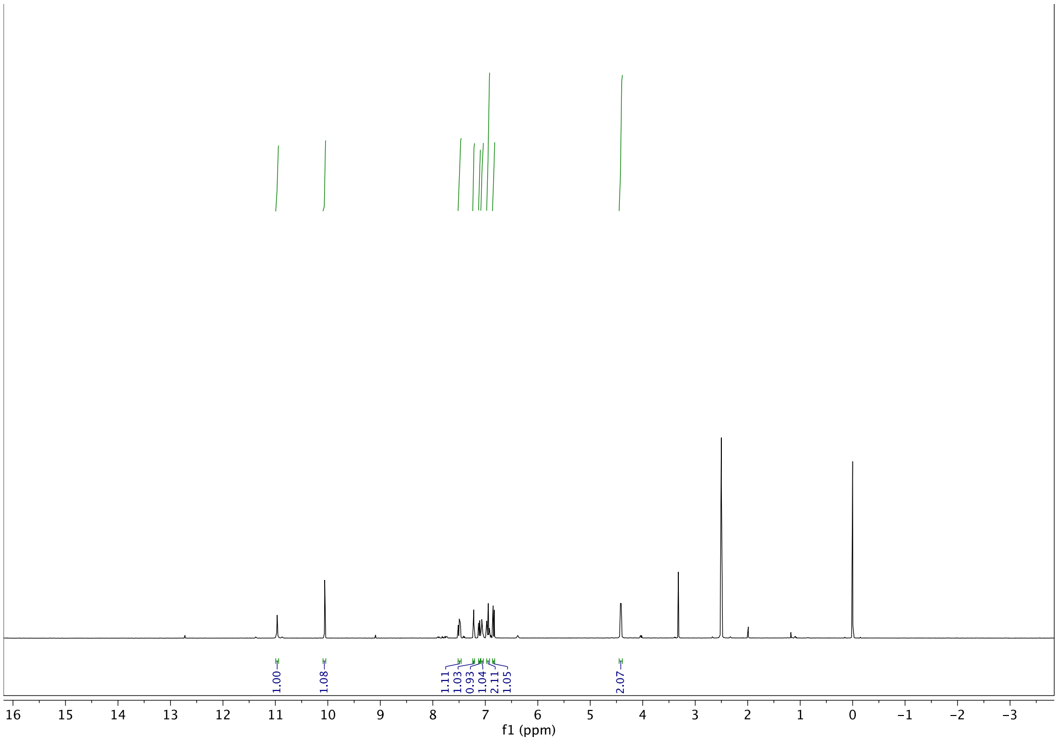

^{13}C NMR:

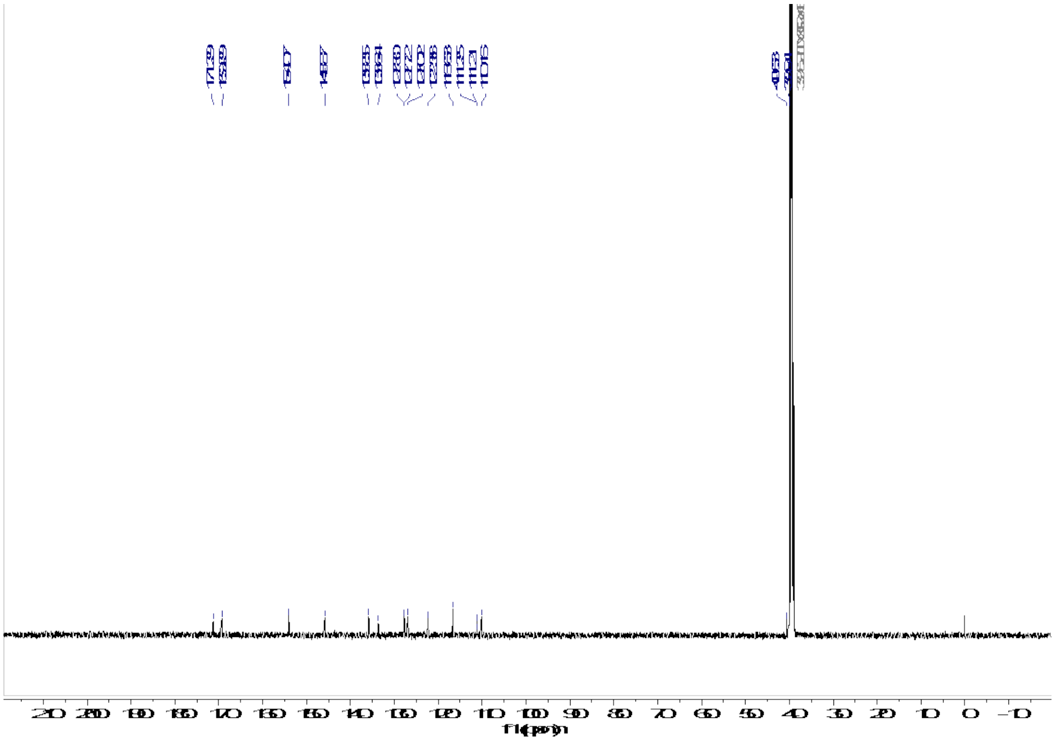

^{1}H and ^{13}C spectra for 4-((5-Bromo-2-hydroxybenzyl)amino)isoindoline-1,3-dione (KM-5-35)

^{1}H NMR:

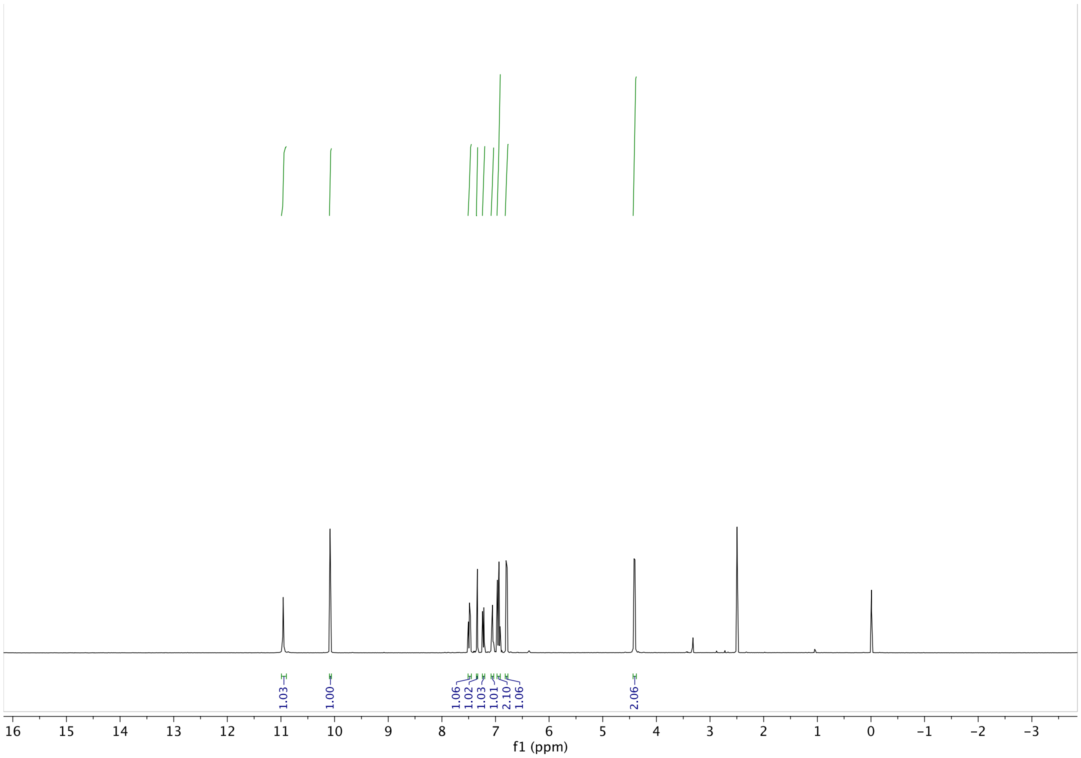

^{13}C NMR:

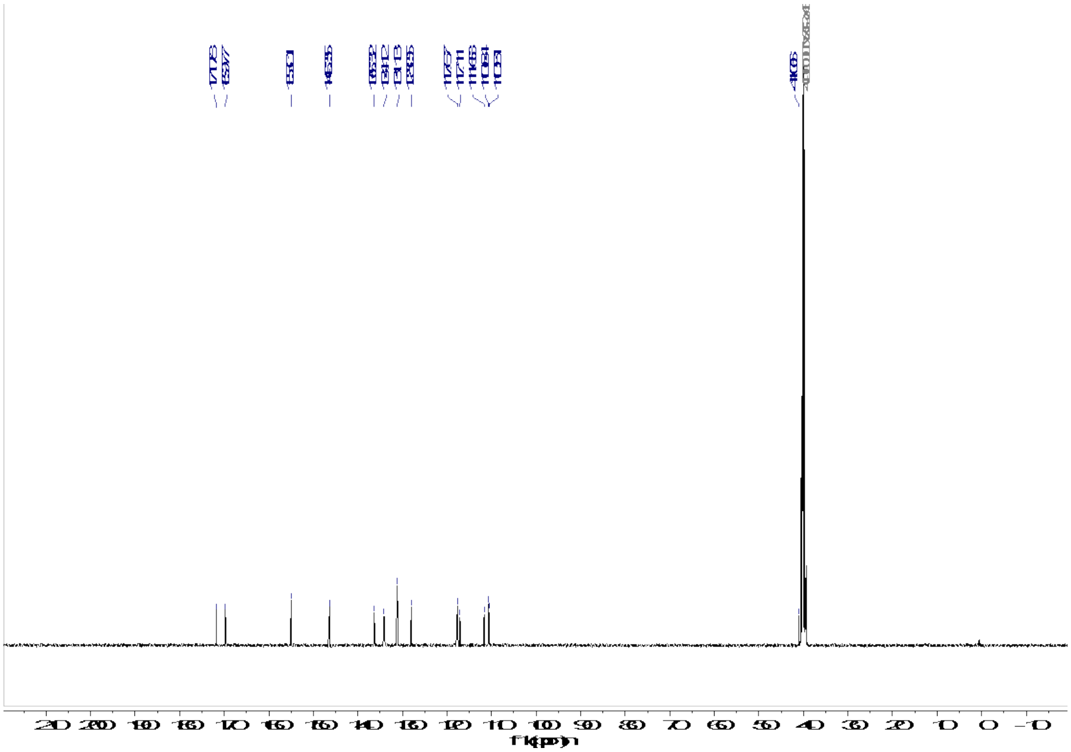

SUPPLEMENTARY FIGURES

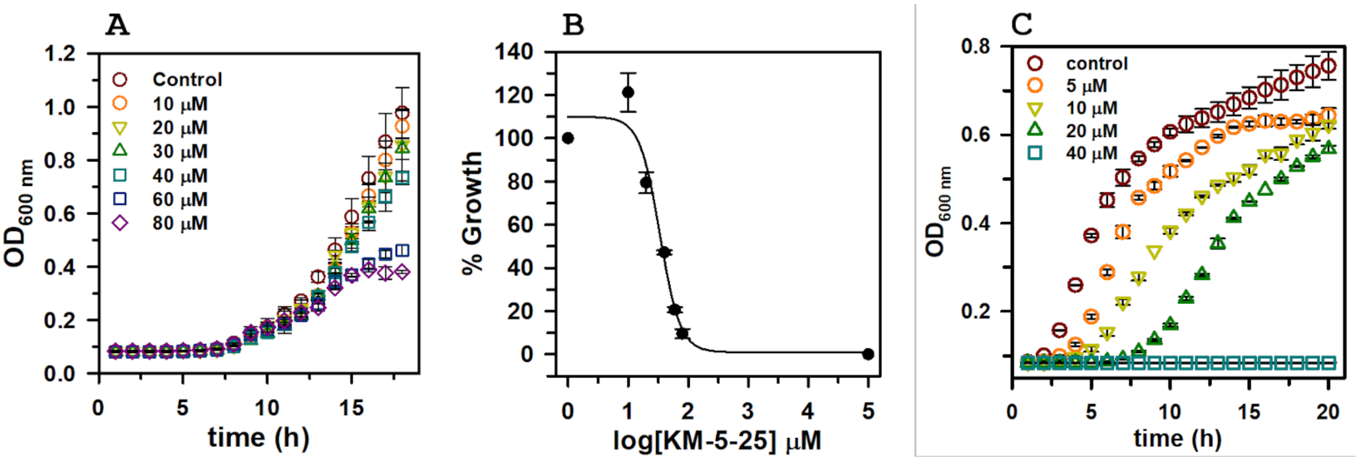

Figure S1. 4-Aminoisoindoline derivatives elicit a growth defect in planktonic *P. aeruginosa* and *A. baumannii*.

(**A**) Concentration-dependent growth retardation of *P. aeruginosa* PAO1 treated with **KM-5-25** and (**B**) associated IC_{50} value obtained from fitting the % growth of *P. aeruginosa* (CFU/mL) as a function of inhibitor concentration to Equation S1; the log [KM-5-25] = 5 value is included to define the minimum asymptote for the fi. (**C**) Concentration-dependent growth retardation of *A. baumannii* 5075 treated with **KM-5-25**. Each of the growth curves was constructed from the average and standard deviation of 3 replicate wells. The IC_{50} and MIC values are the average from three independent experiments.

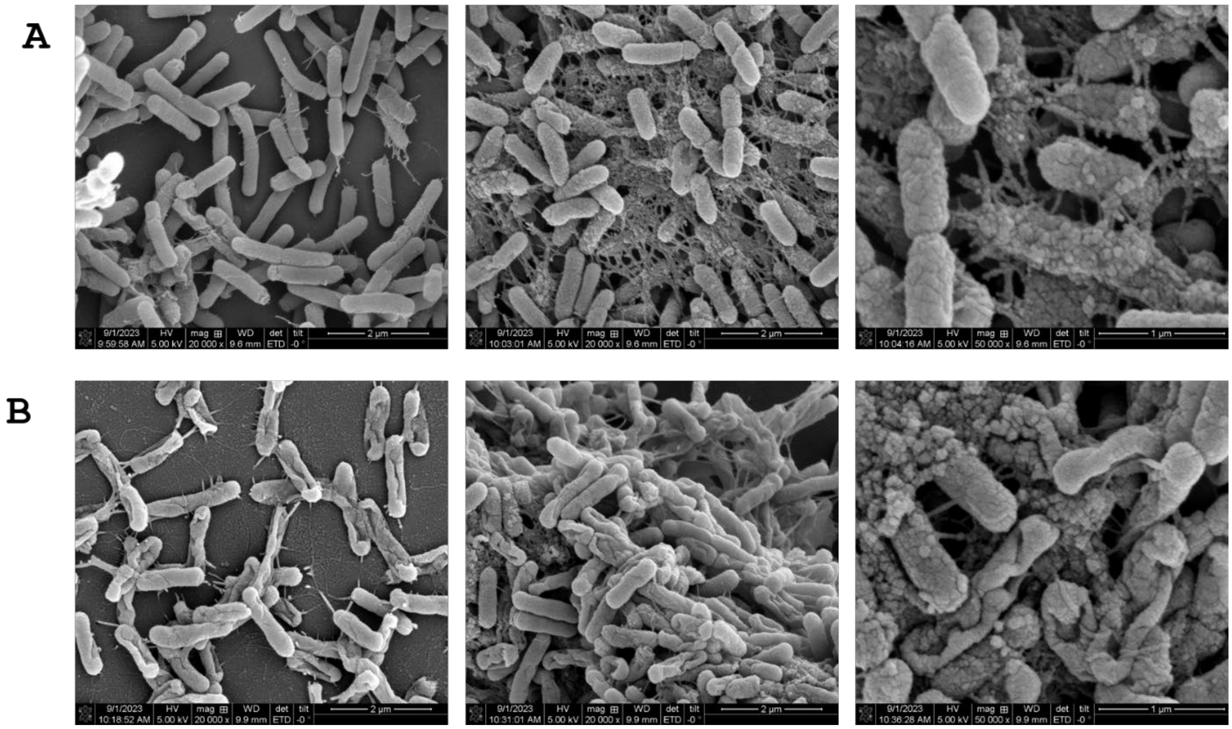

Figure S2. ***P. aeruginosa*** **(PAO1) cells in mature biofilms are susceptible to 4-amino-isoindole-1,3-dione analogues.**

Scanning electron microscopy (SEM) images showing untreated *P. aeruginosa* PAO1 biofilm (A) and biofilm treated with 50 μM **KM-5-25** for 24 h (B). Three representative images are shown for each condition.

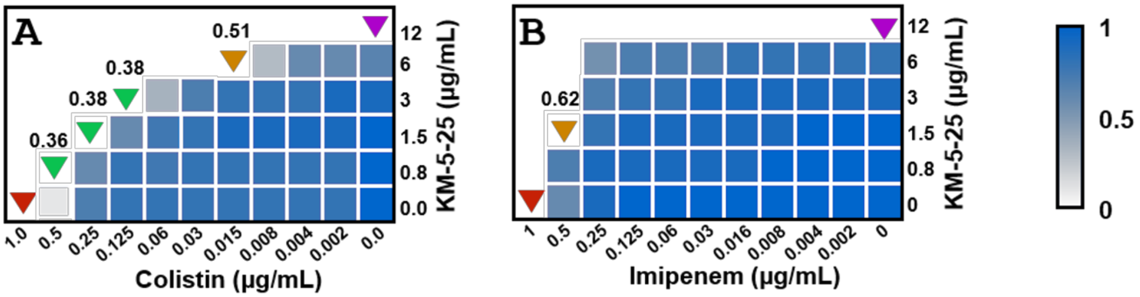

Figure S3. Checkerboard microdilution assay between KM-5-25 and (A) colistin, (B) imipenem against *A. baumannii 5075*.

Bacterial growth monitored by OD_{600} is represented as a heat map from white (no growth) to blue. The red and purple triangles, respectively, indicate MIC values for antibiotic and **KM-5-25**; the green and orange triangles, respectively, indicate concentrations where antibiotic and **KM-5-25** exhibit synergistic and additive interaction. FIC values are noted on top of the green and orange triangles. The data are representative of three biological replicates.

SUPPLEMENTARY TABLES

Table S1. Analogues with an invariant 2-OH and one additional substituent in the aryl ring of the pharmacophore

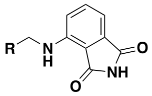

Compound	R	Solubility[1]	K_d (μM)[2]	*P. aeruginosa* PAO1 Intracell. Accum[3]	*P. aeruginosa* PAO1 IC_{50} (μM)	*P. aeruginosa* PAO1 % Survival Biofilm[4]	*A. baumannii* 5075 Intracell. Accum[3]	*A. baumannii* 5075 MIC (μM)	*A. baumannii* 17978 MIC (μM)	*A. baumannii* 19606 MIC (μM)
**Analog 11**	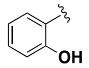	200	33 ± 5	Not detected	178 ± 14	87 ± 6	55 ± 15	80	120	80
**EB-5-80**	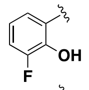	100	49 ± 7	11 ± 3	Not active	106 ± 23	44 ± 6	>100	>100	>100
**KM-5-30**	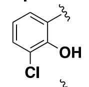	80	11 ± 3	42 ± 1	57 ± 2	72 ± 11	169 ± 62	>80	>80	>80
**EB-5-73**	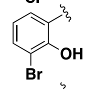	40	7 ± 2	34 ± 9	Not active	78 ± 14 (30 μM)	201 ± 30	>40	40	>40
**EB-5-70**	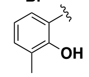	100	17 ± 2	Not detected	99 ± 15	Not active	105 ± 2	100	100	100
**BN-4-74**	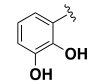	200	69 ± 22	Not detected	Not active	Not active	31 ± 11	200	200	200
**EB-5-62**	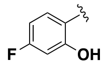	150	24 ± 4	25 ± 3	101 ± 6	61 ± 16	248 ± 86	150	150	75
**EB-5-63**	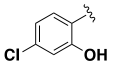	60	24 ± 2	78 ± 3	33 ± 4	12 ± 6	1579 ± 339	50	50	50
**EB-5-61**	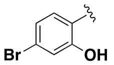	60	13 ± 3	197 ± 17	24 ± 2	8 ± 2	2908 ± 618	50	50	50
**EB-5-69**	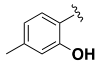	80	45 ± 6	52 ± 2	Not active	64 ± 1	193 ± 33	80	80	80
**BN-16-69**	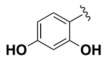	Unstable	–	–	–	–	–	–	–	–
**JAG-5-7**	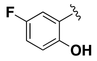	180	24 ± 6	28 ± 10	83 ± 19	85 ± 16	157 ± 54	60	60	60
**KM-5-25**	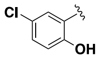	80	10 ± 2	270 ± 48	31 ± 7	17 ± 6	2089 ± 275	40	40	30
**KM-5-35**	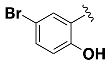	60	9 ± 3	360 ± 22	27 ± 3	10 ± 4	2334 ± 272	40	30	30
**EB-5-1**	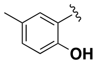	120	38 ± 13	87 ± 15	107 ± 12	63 ± 3	148 ± 44	100	100	80
**EB-5-93**	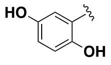	Unstable	–	–	–	–	–	–	–	–
**SB-16-53**	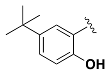	30	1 ± 0.2	263 ± 40‡	12 ± 6	48 ± 10 (30 μM)	327 ± 17‡	30	30	30
**EB-5-71**	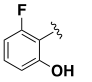	150	56 ± 10	NRV	80 ± 10	52 ± 10	495 ± 35	75	75	75
**KM-5-29**	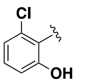	60	18 ± 3	351 ± 25	36 ± 5	50 ± 7	2614 ±134	>60	>60	>60

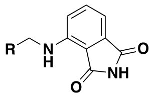

				PAO1			5075	5075	17978	19606
**Compound**	**R**	**Solubility[1]**	**K_d (μM)[2]**	**Intracell. Accum[3]**	**IC_{50} (μM)**	**% Survival Biofilm[4]**	**Intracell. Accum[2]**	**MIC (μM)**	**MIC (μM)**	**MIC (μM)**
**EB-5-72**	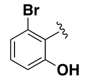	60	15 ± 3	NRV	32 ± 5	45 ± 0.4	2507 ± 355	>60	>60	>60
**SB-15-171**	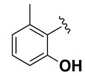	Unstable	–	–	–	–	–	–	–	–
**BN-16-70**	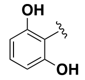	Unstable	–	–	–	–	–	–	–	–

[1]Solubility (μM) in M63 media at 35 ºC; [2]K_d was measured using recombinant Pa BfrB; [3]intracellular accumulation (nmol/10^{12} CFU) when cells are treated with 30 μM compound; ‡treatment with 15 μM compound; [4]biofilm challenged with 50 μM compound unless otherwise stated; NRV = non-reproducible values.

Table S2. Analogues with an invariant 2-OH and two additional substituents in the phenyl ring of the pharmacophore

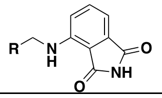

				P. aeruginosa			*A. baumannii*			
				PAO1			5075	5075	17978	19606
**Compound**	**R**	**Solubility[1]**	**K_d (μM)[2]**	**Intracell. Accum[3]**	**IC_{50} (μM)**	**% Survival Biofilm[4]**	**Intracell. Accum[3]**	**MIC (μM)**	**MIC (μM)**	**MIC (μM)**
**EB-5-14**	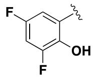	100	14 ± 3	23 ± 8	71 ± 8	84 ± 11	117 ± 21	50	100	50
**EB-5-15**	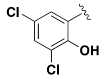	40	8 ± 2	110 ± 20‡	26 ± 3	41 ± 4 (30 μM)	358 ± 92‡	40	40	40
**SB-16-165**	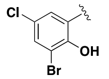	Not soluble	–	–	–	–	–	–	–	–
**SB-16-156**	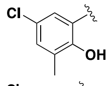	40	7 ± 2	165 ± 18‡	21 ± 5	44 ± 18 (30 μM)	671 ± 89‡	>40	>40	>40
**SB-16-168**	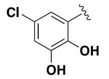	40	12 ± 2	LF	15 ± 7	Not active	LF	40	>40	>40
**SB-16-159**	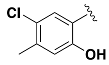	Not soluble	–	–	–	–	–	–	–	–
**SB-16-160**	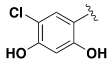	80	77 ± 10	LF	45 ± 6	Not active	LF	>80	>80	>80
**SB-16-152**	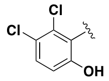	Not soluble	–	–	–	–	–	–	–	–
**SB-16-118**	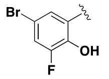	40	4 ± 0.3	126 ± 40	25 ± 8	13 ± 2 (40 μM)	1246 ± 138	20	30	30
**EB-5-13**	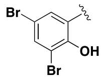	Not soluble	–	–	–	–	–	–	–	–
**SB-16-106**	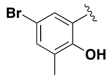	30	3 ± 1	415 ± 141‡	14 ± 4	42 ± 9 (30 μM)	782 ± 11‡	20	15	30
**SB-16-136**	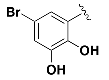	60	7 ± 1	LF	20 ± 5	63 ± 2	LF	40	30	30
**SB-16-112**	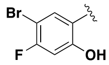	30	2 ± 0.6	457 ± 22‡	14 ± 3	15 ± 0.7 (30 μM)	1073 ± 170‡	20	20	20
**SB-16-137**	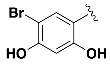	80	9 ± 0.4	33 ± 1	37 ± 4	78 ± 21	86 ± 23	>80	>80	>80
**SB-16-145**	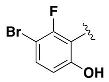	30	6 ± 2	NRV[5]	13 ± 4	54.5 ± 0.7 (30 μM)	4688 ± 991	30	30	30
**SB-16-183**	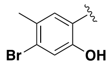	20	3 ± 0.6	379 ± 48‡	13 ± 1	43 ± 3 (20 μM)	1067 ± 211‡	20	20	>20
**SB-16-186**	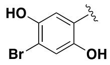	Unstable	–	–	–	–	–	–	–	–

[1]Solubility (μM) in M63 media at 35 ºC; [2]K_d was measured using recombinant Pa BfrB; [3]intracellular accumulation (nmol/10^{12} CFU) when cells are treated with 30 μM compound; ‡treatment with 15 μM compound; [4]biofilm challenged with 50 μM compound, unless otherwise stated; [5]inconsistent values; [LF] = low fluorescence intensity.

Table S3. Analogues with modification in the phthalimide bicycle of the pharmacophore

				P. aeruginosa			*A. baumannii*			
				PAO1			5075	5075	17978	19606
**Compound**		**Solubility**[1]	**K_d (µM)**[2]	**Intracell. Accum**[3]	**IC_{50} (µM)**	**% Survival Biofilm**[4]	**Intracell. Accum**[3]	**MIC (µM)**	**MIC (µM)**	**MIC (µM)**
**SB-16-124**	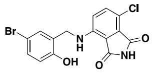	20	11 ± 1	558 ± 154‡	17 ± 2	Not active (20 µM)	2626 ± 389‡	20	20	20
**SB-16-67**	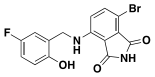	40	7 ± 1	187 ± 59	>40	44 ± 13 (30 µM)	1173 ± 53	40	20	30
**SB-16-51**	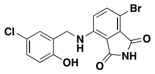	20	13 ± 2	425 ± 79‡	18 ± 3	53 ± 9 (20 µM)	2137 ± 311‡	10	15	15
**SB-15-167**	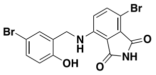	Not soluble	–	–	–	–	–	–	–	–
**SB-16-122**	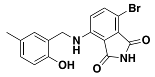	40	5 ± 1	LF	19 ± 4	52 ± 5 (30 µM)	LF	20	20	20
**SB-16-250**	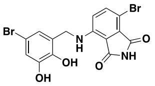	20	15 ± 3	LF	5 ± 2	Not active (20 µM)	LF	>20	>20	>20

[1]Solubility (µM) in M63 media at 35 ºC; [2]K_d was measured using recombinant Pa BfrB; [3]intracellular accumulation (nmol/10^{12} CFU) when cells are treated with 30 µM compound; ‡treatment with 15 µM compound; [4]biofilm challenged with 50 µM compound, unless otherwise stated; LF = low fluorescence intensity.

Table S4. Analogues with a heterocycle in place of the phenyl ring of the pharmacophore

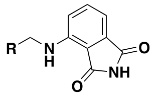

				P. aeruginosa			*A. baumannii*			
				PAO1			5075	5075	17978	19606
**Compound**	**R**	**Solubility**[1]	**K_d (μM)**[2]	**Intracell. Accum**[3]	**IC_{50} (μM)**	**% Survival Biofilm**[4]	**Intracell. Accum**[3]	**MIC (μM)**	**MIC (μM)**	**MIC (μM)**
**SB-17-75**	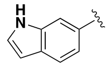	60	15 ± 2	LF	32 ± 13	19 ± 2	LF	30	60	30
**SB-17-140**	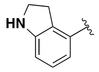	100	37 ± 1	Not detected	Not active	Not active	Not detected	>100	>100	>100
**SB-17-80**	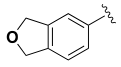	20	11 ± 3	Not detected‡	Not active	Not active (20)	Not detected‡	Not active	Not active	Not active
**SB-16-237**	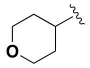	100	42 ± 10	Not detected	Not active	–	Not detected	>100	>100	>100
**SB-17-95**	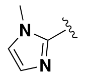	100	57 ± 6	Not detected	Not active	–	Not detected	Not active	Not active	Not active
**SB-17-16**	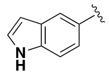	Not soluble	–	–	–	–	–	–	–	–
**SB-17-114**	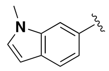	Not soluble	–	–	–	–	–	–	–	–
**SB-17-98**	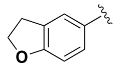	Not soluble	–	–	–	–	–	–	–	–
**SB-17-110**	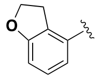	Not soluble	–	–	–	–	–	–	–	–
**SB-17-112**	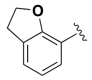	Nots soluble	–	–	–	–	–	–	–	–
**SB-17-109**	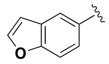	Not soluble	–	–	–	–	–	–	–	–

[1]Solubility (μM) in M63 media at 35 ºC; [2]K_d was measured using recombinant Pa BfrB; [3]intracellular accumulation (nmol/10^{12} CFU) when cells are treated with 30 μM compound; ‡treatment with 15 μM compound; [4]biofilm challenged with 50 μM compound, unless otherwise stated; [LF] = low fluorescence intensity

Table S5. Crystallographic data for the Pa Bfr inhibitor complexes

	KM-5-35	KM-5-25
**Data Collection**		
Unit-cell parameters (Å, °)	*a*=129.97	*a*=129.57
	b=194.96	*b*=194.36
	c=203.57	*c*=203.03
Space group	$C222_1$	$C222_1$
Resolution (Å)[7]	48.74-1.85 (1.88-1.85)	48.59-1.95 (1.98-1.95)
Wavelength (Å)	0.9795	1.0000
Temperature (K)	100	100
Observed reflections	3,021,813	1,284,414
Unique reflections	218,546	185,210
$\langle I/\sigma(I) \rangle$[7]	13.3 (1.6)	12.5 (1.9)
Completeness (%)[7]	100 (100)	100 (100)
Multiplicity[7]	13.8 (13.3)	6.9 (6.7)
R_{merge} (%)[7, 21]	14.9 (189.9)	9.3 (104.3)
R_{meas} (%)[7, 22]	15.5 (197.4)	10.0 (113.1)
R_{pim} (%)[7, 22]	4.2 (53.8)	3.8 (43.5)
$CC_{1/2}$ [7, 23]	0.999 (0.661)	0.999 (0.746)
**Refinement**		
Resolution (Å)	24.13-1.85	38.00-1.95
Reflections (working/test)	207,340/10,998	175,830/9,277
R_{factor} / R_{free} (%)[24]	15.9/18.5	18.0/22.1
No. of atoms (Protein/EB-5-2/Water)	15,398/273/1134	15,270/189/695
**Model Quality**		
R.m.s deviations		
Bond lengths (Å)	0.009	0.010
Bond angles (°)	0.902	0.926
Average *B*-factor ($Å^2$)		
All Atoms	28.1	34.5
Protein	26.6	33.7
EB-5-2	54.0	61.6
Water	37.6	41.6
Coordinate error (maximum likelihood) (Å)	0.18	0.21
Ramachandran Plot		
Most favored (%)	99.6	99.4
Additionally allowed (%)	0.4	0.6

1) Values in parenthesis are for the highest resolution shell.
2) $R_{merge} = \Sigma_{hkl}\Sigma_i |I_i(hkl) - \langle I(hkl)\rangle| / \Sigma_{hkl}\Sigma_i I_i(hkl)$, where $I_i(hkl)$ is the intensity measured for the *i*th reflection and $\langle I(hkl)\rangle$ is the average intensity of all reflections with indices hkl.
3) $R_{factor} = \Sigma_{hkl} ||F_{obs}(hkl)| - |F_{calc}(hkl)|| / \Sigma_{hkl} |F_{obs}(hkl)|$; Rfree is calculated in an identical manner using 5% of randomly selected reflections that were not included in the refinement.
4) R_{meas} = redundancy-independent (multiplicity-weighted) R_{merge}.[7, 21] R_{pim} = precision-indicating (multiplicity-weighted) R_{merge}.[22, 24]
5) $CC_{1/2}$ is the correlation coefficient of the mean intensities between two random half-sets of data.[23, 25]

REFERENCES

1. Stover, C. K.; Pham, X. Q.; Erwin, A. L.; Mizoguchi, S. D.; Warrener, P.; Hickey, M. J.; Brinkman, F. S. L.; Hufnagle, W. O.; Kowalik, D. J.; Lagrou, M.; Garber, R. L.; Goltry, L.; Tolentino, E.; Westbrock-Wadman, S.; Yuan, Y.; Brody, L. L.; Coulter, S. N.; Folger, K. R.; Kas, A.; Larbig, K.; Lim, R.; Simith, K.; Spencer, D.; Wong, G. K. S.; Wu, Z.; Paulsen, I. T.; Reizer, J.; Saler, M. H.; Hancock, R. E. W.; Lory, S.; Olson, M. V. (2000) Complete Genome Sequence of *Pseudomonas aeruginosa* PA01, an Opportunistic Pathogen. *Nature* 406, 959-964. 10.1038/35023079
2. Soldano, A.; Yao, H.; Chandler, J. R.; Rivera, M. (2020) Inhibiting Iron Mobilization from Bacterioferritin in *Pseudomonas aeruginosa* Impairs Biofilm Formation Irrespective of Environmental Iron Availability. *ACS Infect Dis* 6, 447-458. 10.1021/acsinfecdis.9b00398
3. Clark, J.; Maaløe, O. (1967) DNA Replication and the Division Cycle in *Escherichia coli*. *J. Mol. Biol.* 23, 99-112. 10.1016/S0022-2836(67)80070-6
4. Ciccone, L.; Vera, L.; Tepshi, L.; Rosalia, L.; Rossello, A.; Stura, E. A. (2015) Multicomponent mixtures for cryoprotection and ligand solubilization. *Biotechnol Rep (Amst)* 7, 120-127. 10.1016/j.btre.2015.05.008
5. Kabsch, W. (1988) Automatic Indexing of Rotation Diffraction Patterns. *J. Appl. Cryst.* 21, 67-72. 10.1107/S002188988700937
6. Vonrhein, C.; Flensburg, C.; Keller, P.; Sharff, A.; Smart, O.; Paciorek, W.; Womack, T.; Bricogne, G. (2011) Data Processing and Analysis with the AutoPROC Toolbox. *Acta Crystallogr D Biol Crystallogr* D67, 293-302. 10.1107/S0907444911007773
7. Evans, P. R. (2011) An Introduction to Data Reduction: Space-Group Determination, scaling and intentisy statistics. *Acta Cryst.* D67, 282-292. 10.1107/S090744491003982X
8. Wang, Y.; Yao, H.; Cheng, Y.; Lovell, S.; Battaile, K. P.; Middaugh, C. R.; Rivera, M. (2015) Characterization of the Bacterioferritin/Bacterioferritin Associated Ferredoxin Potein-Protein Interactions in Solution and Determination of Binding Energy Hot Spots. *Biochemistry* 54, 6162-6175. 10.1021/acs.biochem.5b00937
9. McCoy, A. J.; Grosse-Kunstleve, R. W.; Adams, P. D.; Winn, M. D.; Storoni, L. C.; Read, R. J. (2007) *Phaser* crystallographic software. *J. Appl. Cryst.* 40, 658-674. 10.1107/S0021889807021206
10. Adams, P. D.; Afonine, P. V.; Brunkózci, G.; Chen, V. B.; Davis, I. W.; Echols, N.; Headd, J. J.; Hung, L.-W.; Kapral, G. J.; Grosse-Kunstleve, R. W.; McCoy, A. J.; Moriarty, N. W.; Oeffner, R.; Read, R. J.; Richardson, D. C.; Richardson, J. S.; Terwilliger, T. C.; Zwart, P. H. (2010) PHENIX: A Comprehensive Python-Based System for Macromolecular Structure Solution. *Acta Cryst.* D66, 213-221. 10.1107/S0907444909052925
11. Emsley, P.; Lohkamp, B.; Scott, W. G.; Cowan, K. (2010) Features and Development of Coot. *Acta Cryst.* D66, 486-501. 10.1107/S0907444910007493
12. Chen, V. B.; Arendall, W. B. r.; Headd, J. J.; Keedy, D. A.; Immormino, R. M.; Kapral, G. J.; Murray, L. W.; Richardson, J. S.; Richardson, D. C. (2010) MolProbity: All-Atom Structure Validation for Macromolecular Crystallography. *Acta Cryst. D* 66, 12-21. 10.1107/S0907444909042073
13. Soldano, A.; Yao, H.; Punchi Hewage, A. N. D.; Meraz, K.; Annor-Gyamfi, J. K.; Bunce, R. A.; Battaile, K. P.; Lovell, S.; Rivera, M. (2021) Small Molecule Inhibitors of the Bacterioferritin (BfrB)-Ferredoxin (Bfd) Complex Kill Biofilm-Embedded *Pseudomonas aeruginosa* Cells. *ACS Infect Dis* 7, 123-140. 10.1021/acsinfecdis.0c00669
14. Sebaugh, J. L. (2011) Guidelines for accurate EC50/IC50 estimation. *Pharm Stat* 10, 128-134. 10.1002/pst.426
15. Andrews, J. M. (2001) Determination of Minimum Inhibitory Concentrations. *J. Antimicrob. Chemother.* 48 Suppl. 1, 5-16. 10.1093/jac/48.suppl_1.5
16. Richter, M. F.; Drown, B. S.; Riley, A. P.; Garcia, A.; Shirai, T.; Svec, R. L.; Hergenrother, P. J. (2017) Predictive compound accumulation rules yield a broad-spectrum antibiotic. *Nature* 545, 299-304. 10.1038/nature22308
17. Ellison, S. L.; Thompson, M. (2008) Standard additions: myth and reality. *Analyst* 133, 992-7. 10.1039/b717660k
18. Louden, B. C.; Haarmann, D.; Lynne, A. M. (2011) Use of Blue Agar CAS Assay for Siderophore Detection. *J Microbiol Biol Educ* 12, 51-3. 10.1128/jmbe.v12i1.249

19. Yao, H.; Rui, H.; Kumar, R.; Eshelman, K.; Lovell, S.; Battaile, K. P.; Im, W.; Rivera, M. (2015) Concerted motions networking pores and distant ferroxidase centers enable bacterioferritin function and iron traffic. *Biochemistry* 54, 1611-27. 10.1021/bi501255r
20. Salmi, C.; Letourneux, Y.; Brunel, J. M. (2006) Efficient synthesis of various secondary amines through a titanium(IV) isopropoxide-mediated reductive amination of ketones. *Lett Org Chem* 3, 396-401. Doi 10.2174/157017806776611845
21. Evans, P. (2006) Scaling and assessment of data quality. *Acta Crystallogr D Biol Crystallogr* 62, 72-82. 10.1107/S0907444905036693
22. Weiss, M. S. (2001) Global indicators of X-ray data quality. *Journal of applied crystallography* 34, 130-135. 10.1107/S0021889800018227
23. Karplus, P. A.; Diederichs, K. (2012) Linking crystallographic model and data quality. *Science* 336, 1030-3. 10.1126/science.1218231
24. Diederichs, K.; Karplus, P. A. (1997) Improved R-factors for Diffraction Data Analysis in Macromolecular Crystallography. *Nat. Struct. Biol.* 4, 269-275. doi.org/10.1038/nsb0497-269
25. Evans, P. (2012) Biochemistry. Resolving some old problems in protein crystallography. *Science* 336, 986-987. 10.1126/science.1222162
